# Design, synthesis, and anticancer evaluation of novel pyrazole–thiophene hybrid derivatives as multitarget inhibitors of wild EGFR, mutant (T790M) EGFR, and VEGFR-2

**DOI:** 10.1039/d5ra06852e

**Published:** 2025-10-22

**Authors:** Mohammed N. Sallam, Ahmed A. Al-Karmalawy, Eslam M. Abbass, Samia S. Hawas, Abeer M. El-Naggar, A. M. A. Hassan

**Affiliations:** a Egypt Otsuka Pharmaceutical Tenth of Ramadan Egypt; b Department of Pharmaceutical Chemistry, College of Pharmacy, The University of Mashreq Baghdad 10023 Iraq akarmalawy@horus.edu.eg; c Department of Pharmaceutical Chemistry, Faculty of Pharmacy, Horus University-Egypt New Damietta 34518 Egypt; d Department of Chemistry, Faculty of Science, Ain Shams University Abbassia 11566 Cairo Egypt ayman.mohammed@sci.asu.edu.eg

## Abstract

A new series of pyrazole–thiophene hybrid derivatives was rationally designed, synthesized, and biologically evaluated for anticancer potential. Cytotoxicity screening toward MCF-7 and HepG2 cell lines identified 2 as the most potent analogue (IC_50_ = 6.57 μM, MCF-7; 8.86 μM, HepG2), with activity comparable to the reference drugs doxorubicin, erlotinib, and sorafenib. Selectivity toward MCF-7 was observed for 8 (IC_50_ = 8.08 μM), while 14 displayed moderate dual activity (IC_50_ = 12.94 μM, MCF-7; 19.59 μM, HepG2). Enzyme inhibition assays revealed that 2 effectively suppressed wild-type EGFR (IC_50_ = 16.25 μg mL^−1^) and the clinically relevant T790M mutant (17.8 μg mL^−1^), whereas 14 showed balanced dual inhibition (16.33 and 16.6 μg mL^−1^, respectively). Notably, 8 emerged as the most active VEGFR-2 inhibitor (35.85 μg mL^−1^), significantly outperforming 14 (112.36 μg mL^−1^) and 2 (242.94 μg mL^−1^). Mechanistic studies demonstrated that 14 brought MCF-7 cells in the G0/G1 phase to cell-cycle arrest (74.16% *vs.* 55.31% in control), increased the apoptotic population to 26.32%, and caused minimal necrosis (4.2%). Molecular docking supported these findings, revealing strong binding affinities and favorable interactions of 2, 14, and 8 with EGFR (wild-type and T790M mutant) and VEGFR-2, respectively. Taken together, these results highlight 2, 8, and 14 as promising pyrazole–thiophene multitargeted anticancer leads, offering potential for further optimization to overcome kinase-driven resistance in cancer therapy.

## Abstract 2

Novel pyrazole–thiophene hybrid derivatives as multitarget inhibitors of wild EGFR, mutant (T790M) EGFR, and VEGFR-2.

## Graphical abstract

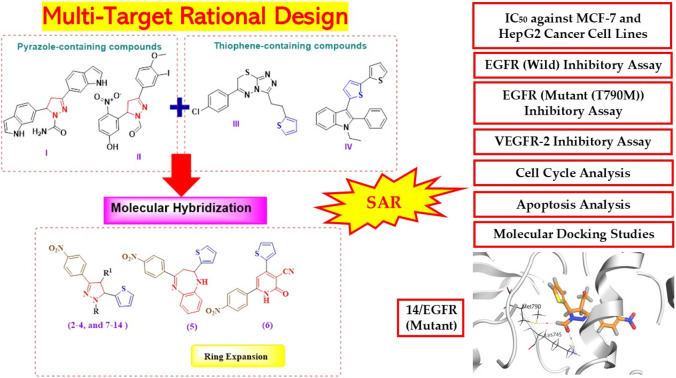

## 1. Introduction

Cancer continues to be a major cause of death globally, driven by complex genetic and molecular abnormalities that disrupt normal cell signaling and proliferation.^1,2^ Among the critical targets in cancer treatment, the epidermal growth factor receptor (EGFR) has become a key regulator, with both wild-type and mutant versions influencing the growth, resistance, and spreading of tumors in a variety of malignancies, including as non-small cell lung carcinoma and breast cancer.^3–5^ Mutations in EGFR often lead to aberrant, ligand-independent activation, promoting unchecked cellular growth and survival.^6–9^

Parallel to EGFR, vascular endothelial growth factor receptor-2 (VEGFR-2) plays a pivotal role in tumor angiogenesis by promoting the development of new blood vessels that supply nutrients to tumors and support their growth.^10,11^ Inhibiting VEGFR-2 not only disrupts this vascular support but also enhances the efficacy of anti-proliferative therapies.^12^

A complementary strategy in anticancer drug development involves targeting the cell cycle, which is a tightly regulated process essential for cell division.^13–15^ Disrupting specific checkpoints can halt cancer cell proliferation and trigger apoptosis, particularly when combined with targeted inhibition of dysregulated receptors like EGFR and VEGFR-2.^16–18^

Multitarget rational design in anticancer is an advanced strategy that seeks to develop single molecules capable of modulating multiple oncogenic pathways, overcoming the limitations of single-target drugs, such as resistance, limited efficacy, and tumor heterogeneity.^19–21^ By integrating pharmacophoric features from different ligands or designing hybrid scaffolds, multitarget agents can achieve synergistic effects, enhance therapeutic outcomes, and reduce the need for combination therapy.^22–24^ A prominent example is the design of dual EGFR/VEGFR inhibitors, where EGFR blockade suppresses tumor proliferation while VEGFR inhibition prevents angiogenesis, together offering a comprehensive anticancer effect.^25–27^

**Fig. 1:**
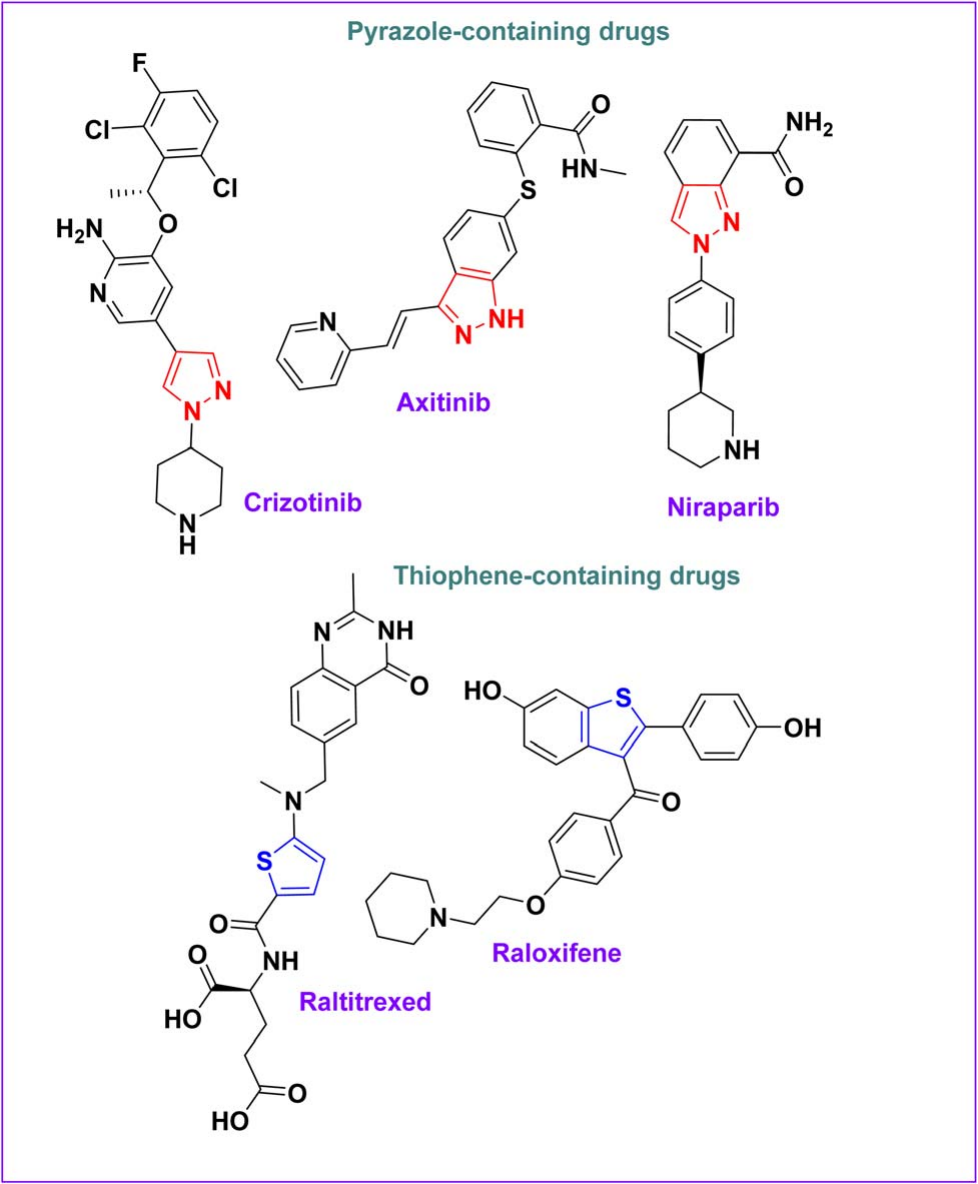
Examples of pyrazole and thiophene-containing drugs.

Pyrazole has garnered significant interest in medicinal chemistry because of its many biological actions, including its anticancer potential.^28–30^ Its electron-rich, flexible structure allows various substitutions to optimize pharmacokinetic and pharmacodynamic properties.^31,32^ Several approved anticancer drugs contain a pyrazole ring, including crizotinib (non-small cell lung cancer),^33^axitinib (VEGFR inhibitor),^34^ and niraparib (poly(ADP-ribose) polymerase inhibitor)^35^ (Fig. 1).

Thiophene is another privileged scaffold with anticancer potential due to its lipophilicity, strong receptor interactions, and metabolic stability.^36–38^ It is found in drugs like raltitrexed (colorectal cancer)^39^ and raloxifene (breast cancer prevention)^40^ (Fig. 1).

**Fig. 2:**
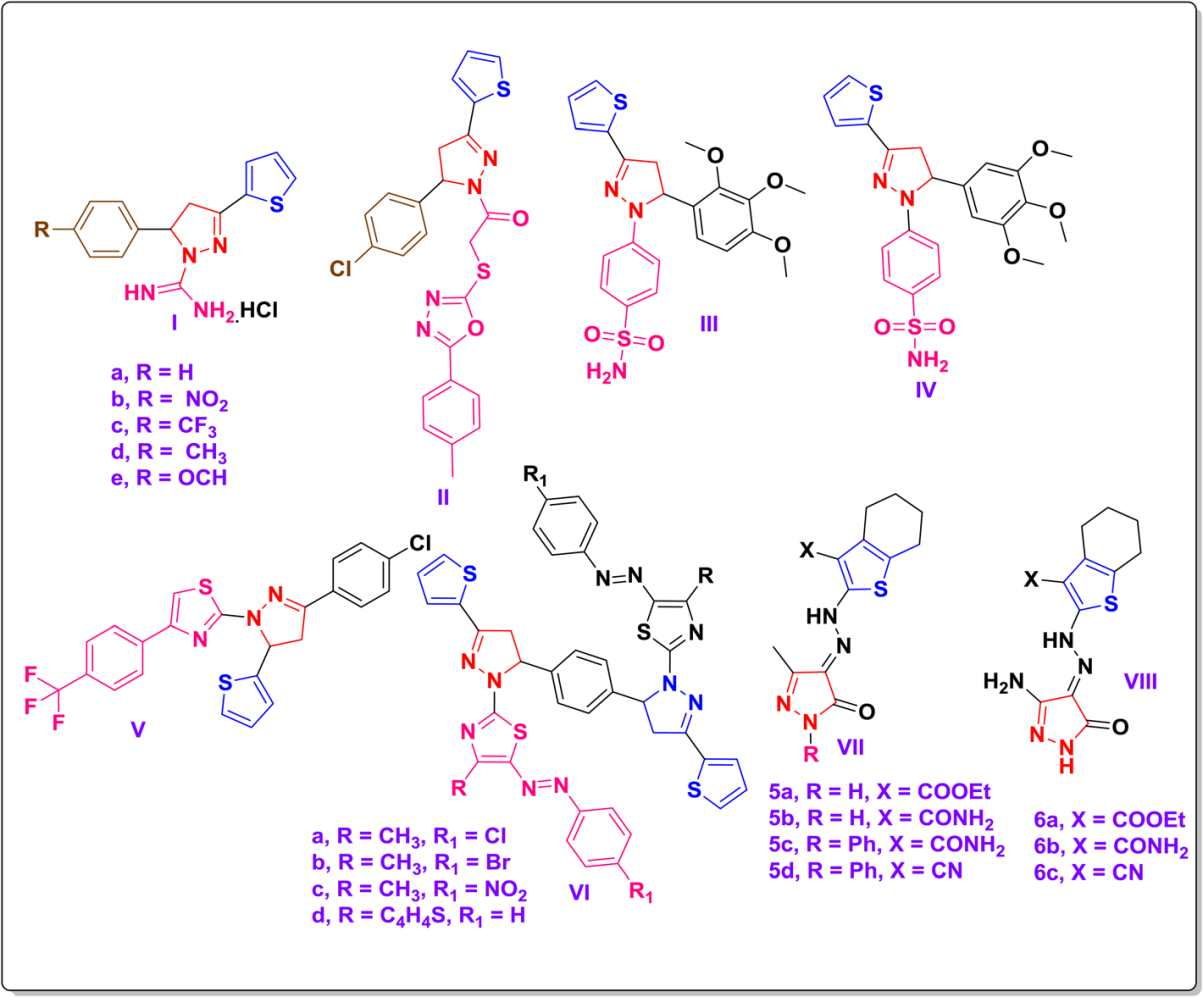
Examples of reported compounds containing both pyrazole and thiophene skeletons.

Combining both moieties has yielded promising anticancer agents.^41^ Examples of compounds containing both pyrazole and thiophene skeletons include: compounds Ia–e (leukemia cytotoxicity, half-maximal inhibitory concentration (IC_50_) = 15–40 μM),^30^ compound II (active against human pancreatic tumor (AsPC-1) and human glioblastoma (U251), IC_50_ ≈ 12–17 μM),^42^ compounds III, and IV (high percentage selectivity index (PSE) values; inhibit human carbonic anhydrase (hCA) XII),^43^ and compound V (nanomolar cytotoxicity; BRAFV600E inhibitor).^44^ In HepG2 cells, compounds VIa–d showed potent cytotoxicity (IC_50_ = 1.37–2.09 μM).^45^ Compounds VIIa–d and VIIIa–c, synthesized *via* diazo coupling, were active against Ehrlich ascites carcinoma^46^ illustrated in Fig. 2.

This work concentrates on the design and assessment of new small molecules containing both pyrazole and thiophene moieties capable of modulating EGFR (both wild-type and mutant (T790M) forms), VEGFR-2, and cell cycle progression, aiming to develop multifunctional agents with enhanced anticancer potential.

### 1.1. Rationale design

Pyrazole and thiophene scaffolds are well established for their anticancer potential, exemplified by pyrazole derivatives such as compound IX (interaction or regulatory relationship between the protein PTPN13 and the Wnt signaling pathway (PTPN13/Wnt) inhibitor, low toxicity)^35^ and compound X (xanthine oxidase inhibitor, pro-apoptotic),^47^ and thiophene-based agents such as compound XI (aromatase and DNA synthesis inhibitor)^48^ and compound XII (glutathione S-transferases (GST) inhibitor, cytotoxic across multiple cell lines).^49^

To utilize these pharmacophores, a molecular hybridization strategy was applied to combine pyrazole and thiophene moieties into a single scaffold, aiming to improve potency, selectivity, and pharmacokinetic characteristics.^50–52^

Structural optimization focused on N1 substitution of pyrazoles with aryl or bulky groups to improve lipophilicity and stability,^53–55^ C3/C5 alkyl/aryl substitutions to modulate binding affinity,^56^ and C4 electron-withdrawing substituents (–CF_3_, –NO_2_, –OCH_3_, –SO_2_NH_2_) to fine-tune electronic properties.^57^

Furthermore, ring-expansion approaches introduced additional heterocycles, including pyridines (increasing planarity and hydrogen bonding)^58^ and benzodiazepines (tricyclic systems with broad bioactivity).^59^

**Fig. 3:**
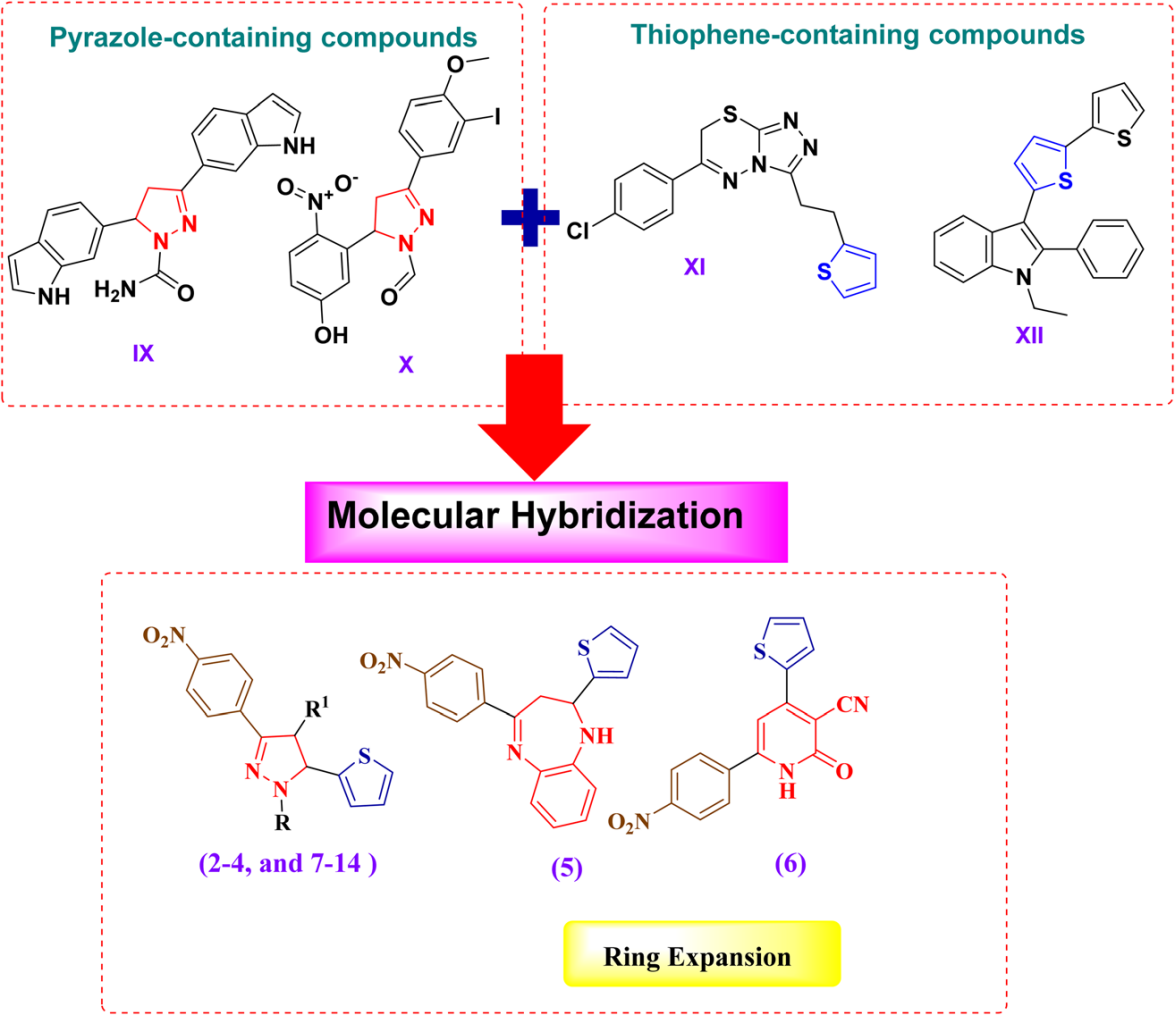
Rationale design of the newly synthesized analogues.

Collectively, these strategies provide a rational design platform for generating novel pyrazole–thiophene hybrids with optimized anticancer activity and favorable drug-like characteristics (Fig. 3).

## 2. Results and discussion

### 2.1. Chemistry

**Scheme 1:**
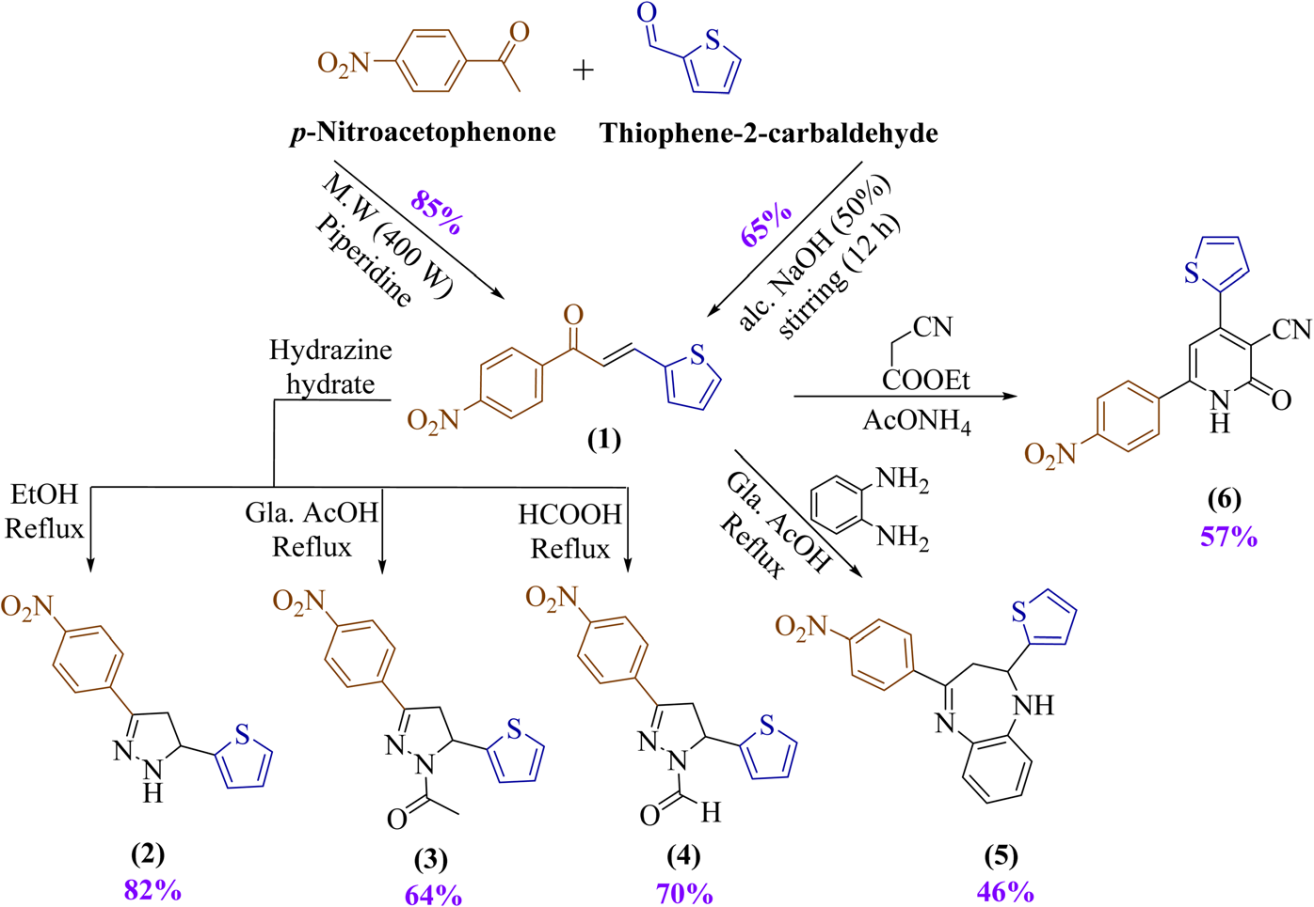
Synthesis of chalcone (1) and utilization to design several novel heterocycles.

α,β-Unsaturated carbonyl compounds, known as chalcone derivatives, are created from the alkenyl moiety of 1,3-diaryl-2-propen-1-one, a reactive ketone. In line with our previous work and as part of our ongoing efforts in synthesizing and characterizing pharmaceutically active heterocycles,^60–63^ encouraged to design 1-(4-nitrophenyl)-3-(thiophen-2-yl)prop-2-en-1-one (1) “chalcone” that bears nitrophenyl and thiophene moieties in its chemical structure.^64–68^ Then utilized compound 1 as a starting material to design novel heterocycles. In the beginning, 1-(4-nitrophenyl)-3-(thiophen-2-yl)prop-2-en-1-one (1) was produced through the reacting thiophene-2-carbaldehyde with *p*-nitroacetophenone using an aqueous potassium hydroxide solution (20%) under a stirring system at room temperature. Notably, the yield improved from 65% to 85% when the reaction was performed under microwave irradiation, as illustrated in Scheme 1. Compound 1 structure was confirmed by FTIR spectroscopy, which displayed absorption bands at *ν* 1654 and 1581 cm^−1^, supportive to the C=O and C=C groups, respectively. Also, by measuring the melting point and mixed melting points, which were detected at m.p. 160–163 (lit., 148–151 °C).^69^

Therefore, under thermal conditions, compound 1 underwent interaction with hydrazine hydrate as a binucleophile agent. This reaction was investigated under different media such as ethanol, glacial acetic acid, and formic acid to afford pyrazole derivatives 2–4, respectively, as illustrated in Scheme 1. Hydrazine was added to chalcone 1 by aza-Michael, leading to the reaction, then 1,5-*exo*-trig cyclization, which is followed by dehydration.

For derivative 2, absorption bands appeared at *ν* 3327 and 1593 cm^−1^ confirming NH and C=N groups, correspondingly. Its spectrum showed a singlet at *δ* 8.35 ppm assigned to the NH proton (disappearing on deuteration) and a triplet at *δ* 5.23 ppm corresponding to the pyrazole CH.

In compound 3, a strong band at *ν* 1666 cm^−1^ confirmed the C=O group, with doublet-of-doublet peaks at *δ* 5.94 and 5.91 ppm for the pyrazole CH, and additional dd signals at *δ* 3.93, 3.89, 3.47, and 3.42 ppm for CH_2_ protons. A singlet at *δ* 2.31 ppm matched the CH_3_ group, and the molecular ion was observed at *m*/*z* 315.

For derivative 4, an absorption band at *ν* 1651 cm^−1^ indicated the C=O group, with a singlet at *δ* 8.93 ppm corresponding to the CHO proton, dd peaks at *δ* 5.94 and 5.91 ppm for the pyrazole CH, and dd peaks at *δ* 4.01, 3.96, 3.52, and 3.48 ppm for CH_2_ protons.

Another binucleophile compound, such as *o*-phenylenediamine, was utilized to interact with chalcone 1 to construct a seven-membered ring heterocycle. Benzodiazepines are bicyclic substances with two nitrogens in the seven-membered ring that belong to a significant class of heterocycles.^70–73^

Condensation of chalcone 1 with *o*-phenylenediamine in ethanol/acetic acid gave benzodiazepine 5, showing characteristic bands at *ν* 3111 and 1676 cm^−1^ for NH and C=N groups. Its spectrum revealed a singlet at *δ* 8.92 ppm (NH, disappearing on deuteration), doublet of doublet peaks at *δ* 6.18 and 6.14 ppm for the pyrazole CH, doublet of doublet peaks at *δ* 4.02, 3.96, 3.52, and 3.46 ppm for CH_2_, and a molecular ion at *m*/*z* 349.

Michael addition between chalcone 1 and ethyl cyanoacetate occurred when there was an excess of ammonium acetate acting as a basic catalyst. The electrophilic β-carbon of the α,β-unsaturated ketone moiety in compound 1 is attacked by the nucleophilic methylene group of ethyl cyanoacetate. A cyclization step usually follows, which eventually results in the creation of 2-pyridone derivative 6, showing bands at *ν* 3363, 2214, and 1650 cm^−1^ for NH, C≡N, and C=O groups. Its spectrum displayed a singlet at *δ* 12.35 ppm (NH, disappearing with deuteration), another singlet at *δ* 6.35 ppm (pyridine CH), and a molecular ion at *m*/*z* 323 (Scheme 1).

Following the verification of the chemical structure of pyrazole derivative 2, an examination of its nucleophilicity was conducted by exploring its interactions with various electrophilic carbonyl compounds. In the beginning, the nucleophilicity of 2 was investigated through its interaction with chloroacetyl chloride using a catalytic amount of triethylamine, which yielded compound 7, exhibiting a band at *ν* 1689 cm^−1^ (C=O). Its spectrum showed a doublet at *δ* 4.15 ppm for CH_2_Cl, a multiplet at *δ* 5.85–5.23 ppm for the pyrazole CH, dd peaks at *δ* 3.94, 3.90, 3.44, and 3.40 ppm for CH_2_ protons, and a carbonyl carbon at *δ* 164.19 in ^13^C-NMR.

**Scheme 2:**
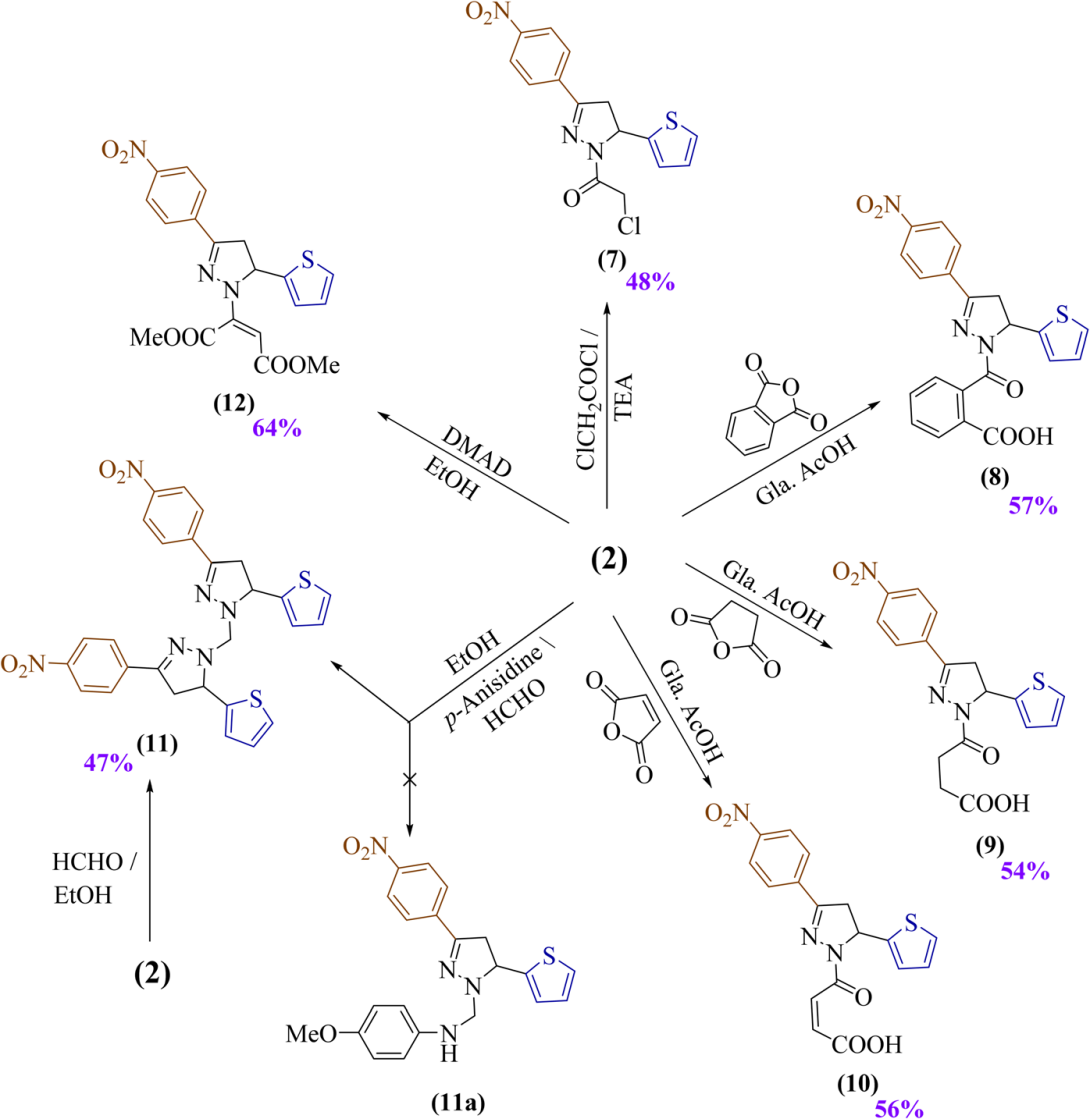
Using pyrazole derivative 2 as a building block to create novel heterocycles.

After that, the treatment of pyrazole derivative 2 with aromatic and aliphatic anhydride derivatives was performed to investigate its nucleophilicity. Phthalic anhydride, succinic anhydride, and malic anhydride were mixed with compound 2 using glacial acetic acid as a solvent to afford acid derivatives 8–10, respectively (Scheme 2). Their spectra showed OH absorption bands near *ν* 3418–3438 cm^−1^, C=O bands for acids at *ν* 1694–1729 cm^−1^, and amide C=O bands at *ν* 1665–1622 cm^−1^.

In 8, a singlet at *δ* 13.14 ppm (COOH, disappearing on deuteration), multiplets at *δ* 7.94–7.22 ppm for aromatic protons, and a molecular ion at *m*/*z* 421 were observed.

In 9, a singlet at *δ* 12.19 ppm (COOH, disappearing on deuteration), triplets at *δ* 2.96 ppm for CH_2_ groups, ^13^C-NMR signals at *δ* 174.16 and 169.94 for acid and amide carbonyls, and a molecular ion at *m*/*z* 373 were detected.

For 10, a singlet at *δ* 12.78 ppm (COOH, disappearing on deuteration) and doublets at *δ* 6.91 and 6.35 ppm for –CH=CH– groups were noted.

Compound 2 was also investigated as a secondary amine compound to undergo a Mannich reaction through its treatment with *p*-anisidine and formaldehyde. This interaction ascertained an unexpected product 11 as a bis-thiazole derivative rather than the expected pyrazole product 11a. The chemical evidence for the existence of the synthesized product 11 was also prepared through the treatment of compound 2 with formaldehyde, only in the absence of *p*-anisidine. Evidence its formation was supported by an absorption band at *ν* 1595 cm^−1^ (C=N), absence of NH signals, a multiplet at *δ* 8.28–6.95 ppm for fourteen aromatic protons, and a molecular ion at *m*/*z* 558.

A regioselective nucleophilic addition of compound 2 to an electron-deficient alkyne group of dimethyl acetylenedicarboxylate (DMAD) was investigated. This reaction afforded compound 12 as a stable *N*-vinylated product in good yield (64%), with absorption bands at *ν* 1746 and 1687 cm^−1^ for ester carbonyls, a singlet at *δ* 4.88 ppm for the olefinic proton, singlets at *δ* 3.86 and 3.52 ppm for two methyl groups, and a molecular ion at *m*/*z* 415 as in Scheme 2.

**Scheme 3:**
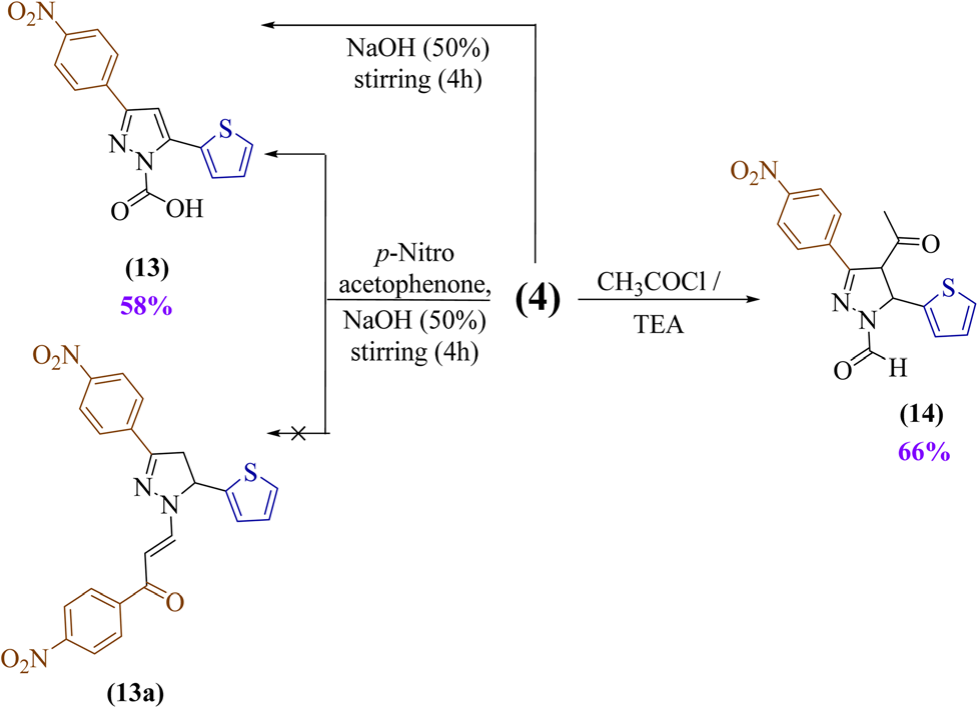
Synthesis of novel pyrazole derivatives (13 and 14).

The attempt to synthesize a unique chalcone 13a by condensation of a synthesized *N*-formyl derivative 4 with *p*-nitroacetophenone in an alkaline condition was unsuccessful. However, this approach resulted in a novel pyrazole derivative with full aromatization bearing a carboxylic acid group (13), indicating that the Cannizzaro reaction occurred mechanically. To confirm this suggestion, this reaction took place again, but without *p*-nitroacetophenone, which afforded the same product 13, as shown in Scheme 3. Its spectra showed absorption bands at *ν* 3210 and 1682 cm^−1^ (OH and C=O), a singlet at *δ* 13.69 ppm (COOH), a singlet at *δ* 7.16 ppm (pyrazole CH), a ^13^C signal at *δ* 147.03 ppm (C=O carbon), and a molecular ion at *m*/*z* 315.

Finally, the treatment of an ethanolic solution of the synthesized *N*-formyl derivative 4 with acetyl chloride in the presence of triethylamine (TEA) produced the acetylated pyrazole derivative 14, displaying a band at *ν* 1654 cm^−1^ (C=O), singlets at *δ* 8.93 ppm (CHO) and 1.67 ppm (CH_3_), and a molecular ion at *m*/*z* 343.

### 2.2. Biological assessment

#### 2.2.1. Analysis of cytotoxic inhibitory concentration 50 (IC_50_) with respect to HepG2 and MCF-7

**Fig. 4:**
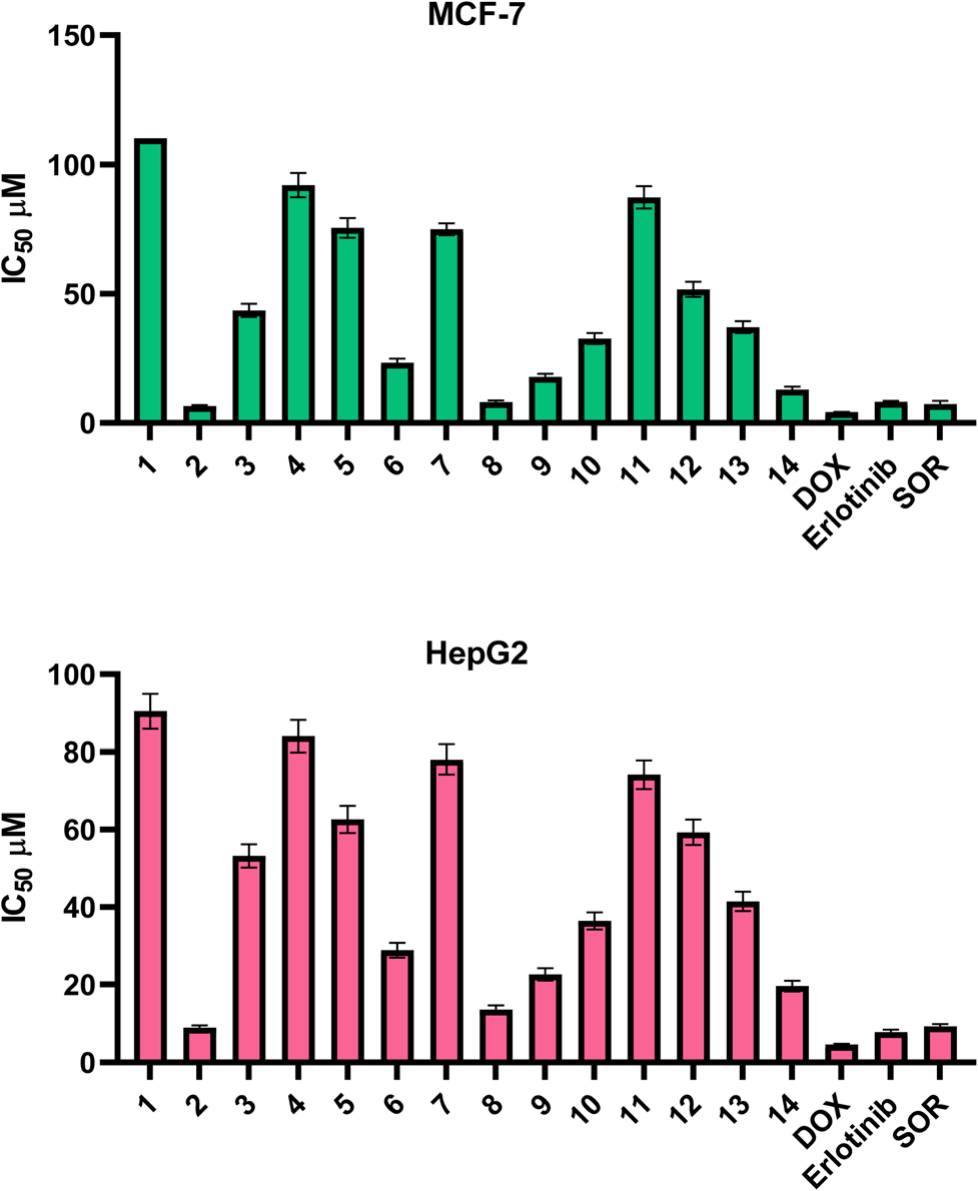
IC_50_ assessment of the compounds (1–14) towards MCF-7 and HepG2 cells.

Two human cancer cell lines, HepG2 (human hepatocellular carcinoma) and MCF-7 (human breast adenocarcinoma), were used to test the produced compounds' cytotoxic activities. MCF-7 cells, derived from breast adenocarcinoma, are reported to exhibit aberrant EGFR signaling that contributes to proliferation and resistance.^74,75^ HepG2 cells, a hepatocellular carcinoma model, are widely used for studying VEGF/VEGFR-2-mediated angiogenesis and tumor progression.^76,77^ Thus, these models provided a relevant system for assessing the potential EGFR/VEGFR inhibitory effects of our synthesized compounds. Accordingly, provide complementary systems for assessing the multitarget inhibitory potential of the synthesized pyrazole–thiophene hybrids against EGFR- and VEGFR-2-mediated oncogenic pathways. Greater cytotoxic potency is shown by lower IC_50_ values, which show the concentration needed to inhibit 50% of cell viability. Compound 2 exhibited the highest cytotoxic activity against both cancer cell lines out of all the compounds that were examined. It displayed an IC_50_ value of 8.86 μM against HepG2 and 6.57 μM against MCF-7, placing it in close range of the reference drugs doxorubicin (4.17 μM for MCF-7, 4.50 μM for HepG2), erlotinib (8.20 μM for MCF-7, 7.73 μM for HepG2),^78^ and sorafenib (7.26 μM for MCF-7, 9.18 μM for HepG2). This indicates that 2 holds promising potential as a dual-active anticancer agent. Following that, 8 demonstrated considerable activity, particularly against MCF-7 (8.08 μM), although it showed moderate efficacy against HepG2 (13.51 μM). 14 ranked next in potency, with an IC_50_ of 12.94 μM against MCF-7 and 19.59 μM against HepG2, suggesting slightly higher selectivity toward the breast cancer cell line (Fig. 4).

Compound 9 displayed weaker cytotoxicity in comparison, with IC_50_ values of 17.80 μM for MCF-7 and 22.62 μM for HepG2. A similar trend was seen with 6 (23.29 μM for MCF-7, 28.87 μM for HepG2) and 10 (32.65 μM for MCF-7, 36.44 μM for HepG2), both of which showed only modest activity. Compounds with further reduced potency included 13 (37.10 μM, MCF-7; 41.42 μM, HepG2), 3 (43.60 μM, MCF-7; 53.18 μM, HepG2), and 12 (51.74 μM, MCF-7; 59.31 μM, HepG2). 5 showed minimal inhibition, with MCF-7 IC_50_ values of 75.54 μM. and 62.63 μM against HepG2. Likewise, 7 was weakly active, showing IC_50_ values of 75.01 μM and 78.08 μM for MCF-7 and HepG2, respectively. Among the least effective were 11 (87.36 μM, MCF-7; 74.18 μM, HepG2) and 4 (92.12 μM, MCF-7; 84.04 μM, HepG2). 1 demonstrated negligible cytotoxicity with IC_50_ values exceeding 100 μM against MCF-7 and 90.48 μM against HepG2 (Fig. 4 and SI Table S1).

##### 2.2.1.1. Structure–activity relationship (SAR)

**Fig. 5:**
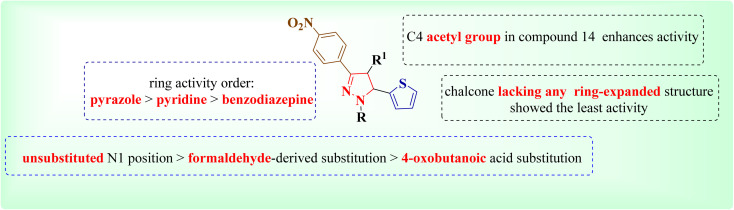
Summary of the SAR of the newly synthesized derivatives.

The structure–activity relationship analysis reveals several key insights regarding the influence of N1 substitution and ring modifications on cytotoxic activity (Fig. 5).

(a) Compound 2 showed the strongest cytotoxic action against MCF-7 and HepG2 cells and has an unsubstituted N1 position on the pyrazole ring.

(b) This highlights the potential benefit of preserving the free N1 position for enhanced biological interaction.

(c) Introducing a benzoic acid-derived acetyl group at N1, as seen in compound 8, retained moderate activity, suggesting that aromatic substitution at this position can still support reasonable potency.

(d) Interestingly, compound 14, which also contains a formaldehyde-derived substitution at N1 along with an additional acetyl group at C4 of the pyrazole ring, showed slightly lower but still notable activity, indicating that dual substitution may modulate the activity profile rather than abolish it.

(e) A comparison between compound 14 and compound 4 (which shares the N1 formaldehyde group but lacks substitution at the pyrazole C4) suggests that the C4 acetyl group in compound 14 may enhance activity within this structural series.

(f) On the other hand, compound 9, which carries a 4-oxobutanoic acid moiety at N1, showed reduced efficacy compared to compound 8, suggesting that increasing the length or polarity of the substituent can affect interaction with the biological target.

(g) Compounds with ring-expanded systems displayed varied activity depending on the nature of the fused or modified heterocycle. For instance, compound 6, featuring a pyridine ring instead of a pyrazole, retained measurable cytotoxicity, though lower than its pyrazole analogs. In contrast, compound 5, where the pyrazole scaffold was expanded to a benzodiazepine ring, demonstrated relatively diminished potency, supporting the trend: pyrazole > pyridine > benzodiazepine in terms of retained cytotoxic activity.

(h) Other N1 substitutions, such as in compound 10 (oxobut-2-enoic acid), compound 13 (formic acid), compound 3 (acetyl), and compound 12 (dimethyl maleate), resulted in reduced activity compared to compound 2, but still provided valuable information regarding steric and electronic tolerance at this position. Compound 7, with an acetyl chloride group at N1, and compound 11, bearing a bulkier pyrazole-based N1 substituent, also demonstrated lower activity, possibly due to steric hindrance or reduced cell permeability.

(i) Lastly, compound 1, a chalcone lacking any pyrazole or ring-expanded structure, exhibited the weakest cytotoxic effect across both cell lines, reinforcing the importance of the pyrazole core—or its closely related analogs—in maintaining biological activity.

Overall, these observations emphasize that an unsubstituted N1 position favors activity, while certain N1 modifications, especially aromatic or appropriately balanced substituents, can be tolerated or even beneficial. The trend in heterocyclic core preference (pyrazole > pyridine > benzodiazepine) further underscores the significance of scaffold selection in future optimization efforts.

#### 2.2.2. EGFR enzyme inhibition assay (wild and mutant (T790M) types) and VEGFR-2 enzyme inhibition assay

**Fig. 6:**
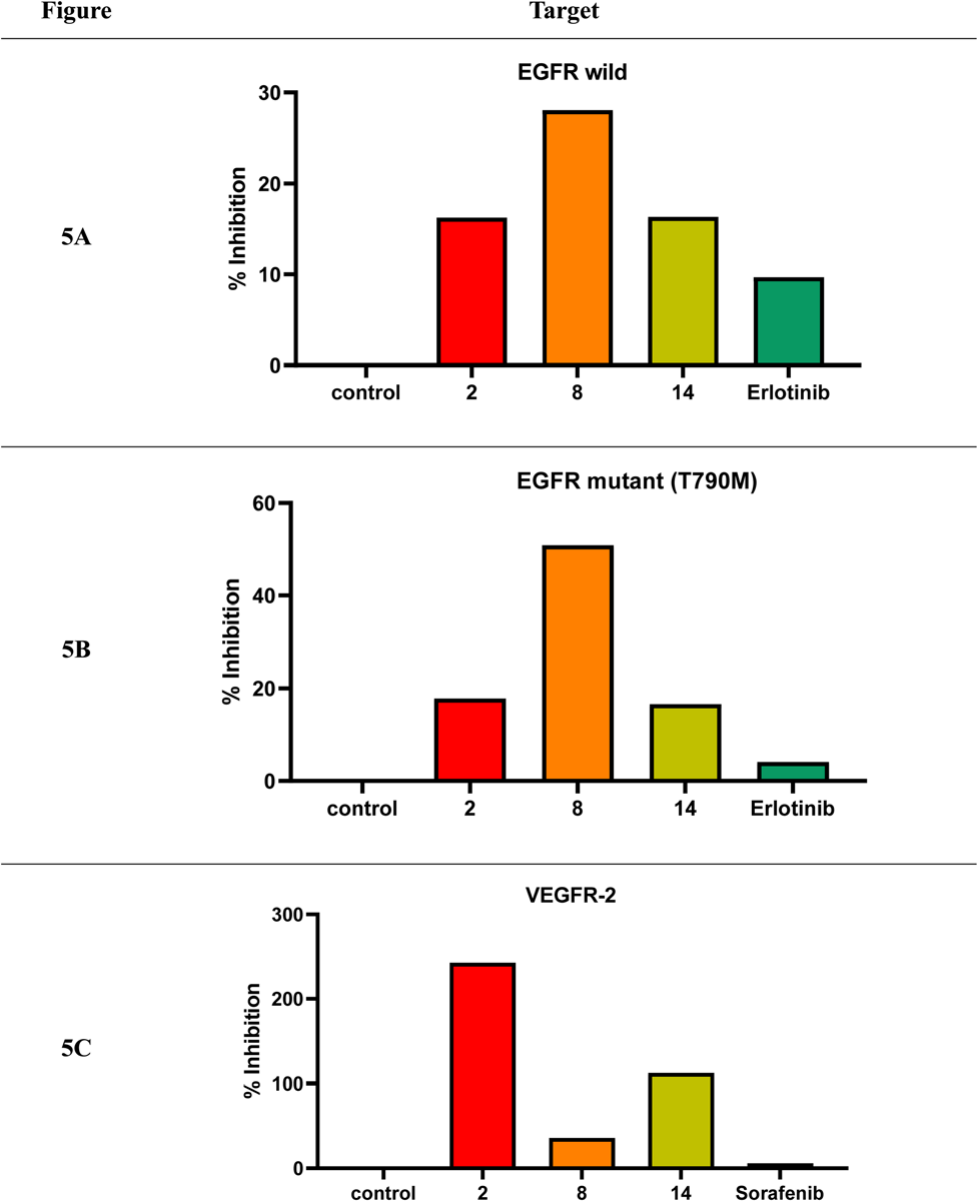
Multitarget enzyme inhibition of compounds 2, 8, and 14 against wild-type EGFR, mutant (T790M) EGFR, and VEGFR-2, compared with reference drugs.

With an IC_50_ of 16.25 μg mL^−1^, compound 2 demonstrated the greatest inhibitory effect against wild-type EGFR, followed closely by 14 (IC_50_ = 16.33 μg mL^−1^). In contrast, 8 showed a weaker EGFR inhibition profile, with an IC_50_ of 28.06 μg mL^−1^ (Fig. 6A).

A similar pattern was observed in the EGFR mutant (T790M) inhibition assay, where 14 again demonstrated superior activity (IC_50_ = 16.6 μg mL^−1^), followed by 2 (IC_50_ = 17.8 μg mL^−1^). 8 remained the least potent in this context, having a 50.9 μg per mL IC_50_. For comparison, the reference drug erlotinib showed IC_50_ values of 9.69 μg mL^−1^ (wild-type EGFR) and 4.13 μg mL^−1^ (mutant EGFR) (Fig. 6B).

Regarding VEGFR-2 inhibition, compound 8 emerged as the most effective among the tested candidates (IC_50_ = 35.85 μg mL^−1^), although it was still significantly less potent than sorafenib (IC_50_ = 5.86 μg mL^−1^). Compound 14 displayed lower activity (IC_50_ = 112.36 μg mL^−1^), while compound 2 was the weakest VEGFR-2 inhibitor (IC_50_ = 242.94 μg mL^−1^), Fig. 6C.

Consequently, compounds 2, 8, and 14 demonstrated promising multitarget inhibitory profiles against wild EGFR, mutant (T790M) EGFR, and VEGFR-2, making them worthy for further optimization (Fig. 6 and SI Table S2).

#### 2.2.3. Assessment of the impact of compound 14 on cell cycle progression and apoptosis in MCF-7 cells

**Fig. 7:**
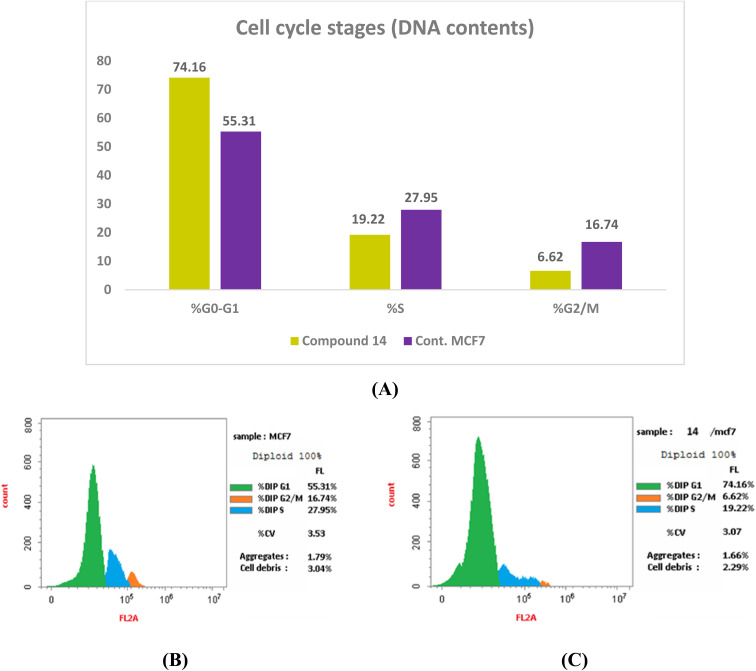
Assessment of the cell cycle: (A) distribution graph of compound 14's cell cycle. (B) and (C) The untreated and 14-treated MCF-7 cells' relative flow cytometry profiles, correspondingly.

The distribution of cell cycle stages in MCF-7 cells treated with compound 14 differed significantly from the untreated control, according to flow cytometry analysis.^79,80^ The G0/G1 phase population rose from 55.31% (control) to 74.16% after treatment with compound 14, suggesting a significant cell cycle arrest at the G1 phase. Concurrently, there was a noticeable decrease in the S phase (from 27.95% to 19.22%) and G2/M phase (from 16.74% to 6.62%), confirming that G1 arrest is a primary mechanism of action (Fig. 7 and SI Table S3).

In addition to cell cycle arrest, treatment with compound 14 markedly enhanced apoptosis. Early and late apoptotic cell populations rose from 0.69% and 0.14% in control cells to 15.02% and 7.1%, respectively, culminating in a total apoptosis rate of 26.32%. Necrosis remained low (4.2%), indicating that the compound predominantly induces programmed cell death rather than nonspecific cytotoxicity.

Collectively, these results suggest that compound 14 primarily causes G1 phase cell cycle arrest and promotes apoptosis in MCF-7 cells to produce its antiproliferative effect.

**Fig. 8:**
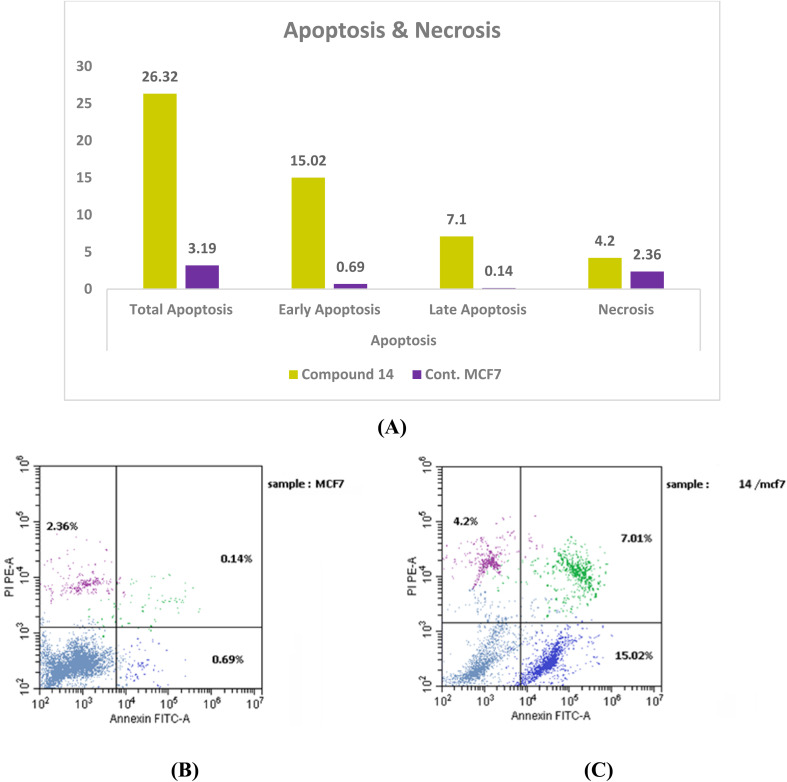
Assessment of the cell cycle: (A) compound 14 apoptosis phases and necrosis, (B) and (C) Dot plots of MCF-7 control and MCF-7 treated with 14, respectively.

The minimal necrotic response further supports its potential safety profile, as it does not appear to cause significant damage to surrounding healthy cells (Fig. 8 and SI Table S4).

### 2.3. Molecular docking

Molecular docking for the frontier analogues 2, 14, and 8 against wild EGFR, mutant (T790M) EGFR, and VEGFR-2 receptors, respectively, was performed to investigate their molecular interactions.

The docking scores of analogues 2, 14, and 8 were recorded at −5.37, −6.96, and −7.14 kcal mol^−1^, compared to −8.77, −8.19, and −10.10 kcal mol^−1^ of the co-crystal inhibitors of wild EGFR, mutant (T790M) EGFR, and VEGFR-2 receptors, respectively. On the other side, analogues 2, 14, and 8 showed acceptable RMSD (root mean square deviation) values of 1.30, 1.51, and 1.73 Å, respectively. Also, the RMSD values of the co-crystal inhibitors of wild EGFR, mutant (T790M) EGFR, and VEGFR-2 receptors were found to be 1.52, 1.71, and 1.49 Å, respectively.

**Fig. 9:**
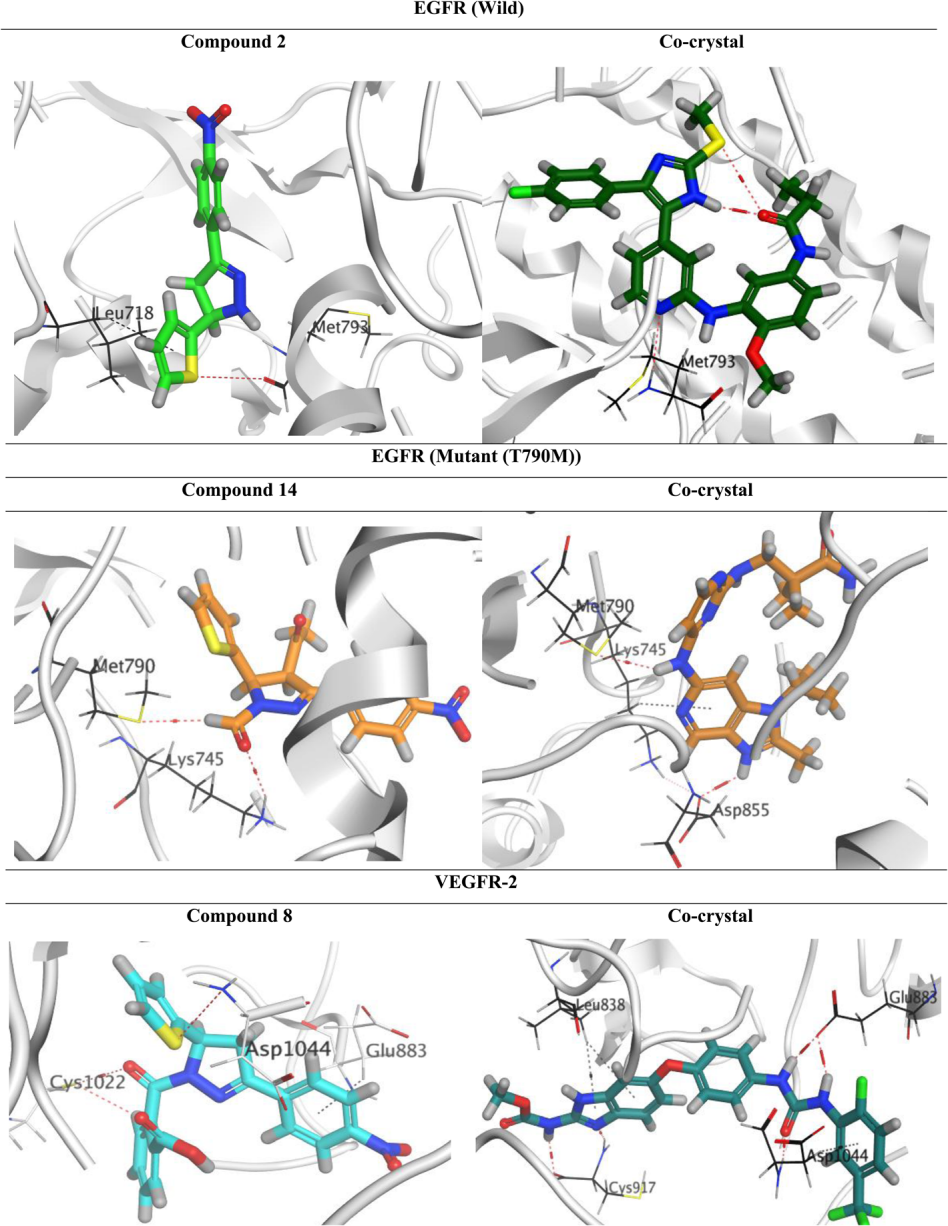
3D Binding interactions for the frontier analogues 2, 14, and 8 against wild EGFR, mutant (T790M) EGFR, and VEGFR-2 receptors (PDB IDs: 6VHN, 5CAL, and 2OH_4_), respectively.

Compound 2 established one hydrogen bond with Met793 and one pi–hydrogen bond with Leu718 at distances of 3.42 and 4.29 Å, respectively, inside the active pocket of wild EGFR. The co-crystal inhibitor of wild EGFR showed one hydrogen bond with Met793 at a distance of 3.44 Å. Additionally, compound 14 established two hydrogen bonds with Lys745 and Met790 at distances of 3.52 and 3.79 Å, respectively, in the mutant (T790M) EGFR's active region. The co-crystal inhibitor of mutant (T790M) EGFR described two hydrogen bonds with Met790 and Asp855 at distances of 3.65 and 3.00 Å, respectively, in addition to a pi–hydrogen bond with Lys745 at a distance of 4.74 Å. Furthermore, compound 8 represented one hydrogen bond with Cys1022 and three pi–hydrogen bonds with Cys1022, Glu883, and Asp1044 of the VEGFR-2 receptor at distances of 3.59, 3.54, 3.90, and 3.43 Å, respectively. The co-crystal inhibitor of VEGFR-2 got five hydrogen bonds with Cys917 (2), Glu883 (2), and Asp1044, at distances of 2.90, 3.20, 2.94, 2.99, and 3.02 Å, respectively, besides three pi–hydrogen bonds with Asp1044 and Leu838 (2) at distances of 3.84, 4.34, and 4.65 Å, respectively (Fig. 9).

## 3. Conclusion

In conclusion, the effective synthesis and evaluation of a novel series of pyrazole–thiophene hybrids produced several candidates with noteworthy anticancer potential. Among them, 2, 8, and 14 emerged as the most promising leads. Compound 2 showed substantial suppression of both wild-type and T790M-mutant EGFR and the strongest dual cytotoxic action against the MCF-7 and HepG2 cell lines, highlighting its potential as a broad-spectrum kinase inhibitor. 14 showed balanced EGFR inhibition and was further validated through mechanistic studies, where it induced G0/G1 cell-cycle arrest, enhanced apoptosis, and maintained a low necrotic effect, suggesting a selective and safer mode of action. In contrast, 8 displayed pronounced VEGFR-2 inhibition, highlighting scaffold-dependent kinase selectivity within the series. Molecular docking studies provided further support for the experimental findings, revealing favorable binding affinities and critical interactions of 2, 14, and 8 with EGFR (wild-type and T790M) and VEGFR-2 active sites. These complementary computational and biological results reinforce the concept of pyrazole–thiophene hybrids as multitargeted anticancer scaffolds with the capacity to address kinase-driven resistance mechanisms. Collectively, this work identifies 2, 8, and 14 as compelling leads for further optimization and paves the way for their advancement into preclinical studies as potential multitargeted anticancer agents.

## 4. Methods & materials

### 4.1. Chemistry

A Gallen Kamp melting point device was used to determine the melting points, which were then reported as uncorrected. A Pye-Unicam SP-3-300 infrared spectrophotometer was used to record the FTIR spectra, which were then converted to wavenumbers (cm^−1^). A Varian Gemini spectrometer was used to obtain ^1^H-NMR spectra at 400 MHz and ^13^C-NMR spectra at 100 MHz in deuterated dimethyl sulfoxide (DMSO-d_6_), with TMS serving as an internal standard. All coupling constants (*J*) are given in hertz; however, chemical shifts (*δ*) are reported in ppm and represent solvent effects. A Shimadzu GC-MS-QP 1000X spectrometer running at 70 eV was used to acquire mass spectra. Using Merck 60 F_254_ UV-fluorescent silica gel plates and thin-layer chromatography (TLC), the reaction's progress was observed under a UV lamp. HPLC was conducted using a Shimadzu HPLC system with column Kromasil C18 micron of 150 × 4.6 mm.

#### 4.1.1. Synthesis of 1-(4-nitrophenyl)-3-(thiophen-2-yl)prop-2-en-1-one (1)

##### 4.1.1.1. Conventional method

A mixture of *p*-nitroacetophenone (10 mmol, 1.65 g) and thiophene-2-carbaldehyde (10 mmol, 1.12 g) in absolute ethanol (20 mL) was stirred vigorously, and 20% aqueous potassium hydroxide solution (4 mL) was added. The reaction was allowed to run for 12 hours at room temperature, and TLC was used to track its progress on plates that had already been coated. After it was completed, 1N hydrochloric acid was used to neutralize the reaction mixture. Filtration and recrystallization of the resultant precipitate from 1,4-dioxane produced the pure product in 65% yield as golden yellow crystals.

##### 4.1.1.2. Microwave irradiation technique

Equimolar amounts of *p*-nitroacetophenone (10 mmol, 1.65 g) and thiophene-2-carbaldehyde (10 mmol, 1.12 g) were combined and dissolved in a minimal volume (3 mL) of *N*,*N*-dimethylformamide (DMF). The mixture was then vigorously mixed before 0.5 mL of piperidine, a catalytic quantity, was added dropwise. For around six minutes, the reaction mixture was exposed to 400 W of microwave radiation. The liquid was then poured onto crushed ice and neutralized using a solution of 1N hydrochloric acid. The pure product was achieved in a reasonable yield of 85%, m.p. 160–163 (lit., 148–151 °C).^69^ FT-IR (KBr, *ν*/cm^−1^): 1654 (C=O), 1581 (C=C).

#### 4.1.2. Synthesis of 3-(4-nitrophenyl)-5-(thiophen-2-yl)-4,5-dihydro-1*H*-pyrazole (2)

Under TLC monitoring, compound 1 (10 mmol, 2.59 g) and hydrazine hydrate (20 mmol, 1 mL) were refluxed in ethanol (20 mL) for five hours. Diluted hydrochloric acid (1N) was used to acidify the reaction liquid after it had cooled and been poured onto crushed ice. By filtering out the precipitate, rinsing it with water, drying it, and recrystallizing it from ethanol, compound 2 was synthesized. Yield 82%; yellow powder; m.p. 118–120 °C. FT-IR (KBr, *ν*/cm^−1^): 3327 (NH), 3109 (CH_arom_), 2902, 2832 (CH_aliph_), 1593 (C=N). ^1^H-NMR (400 MHz, DMSO-*d*_6_) *δ* (ppm): 8.35 (s, 1H, NH, exchangeable with D_2_O), 8.23–7.82 (m, 4H, 4-nitrophenyl ring), 7.43–6.98 (m, 3H, thienyl ring), 5.23 (t, 1H, (**CH**)pyrazole), 3.56, 3.51, 2.97, 3.03 (2 dd, 2H, (**CH**_2_)pyrazole). HPLC purity (C18, ACN/H_2_O/MeOH 30/65/5, 1.0 mL min^−1^, UV 250 nm, 25 μL; isocratic): RT = 12.3 min, % purity = 99.14%.

#### 4.1.3. Synthesis of 1-(3-(4-nitrophenyl)-5-(thiophen-2-yl)-4,5-dihydro-1*H*-pyrazol-1-yl) ethan-1-one (3)

In glacial acetic acid (10 mL), compound 1 (10 mmol, 2.59 g) and hydrazine hydrate (20 mmol, 1 mL) were refluxed for 6 hours while being monitored by TLC. The reaction mixture was cooled and then poured on top of crushed ice. The resultant precipitate was filtered, washed with water, dried, and recrystallized from 1,4-dioxane to obtain compound 3. Yield 64%; pale brown powder; m.p. 191–193 °C. FT-IR (KBr, *ν*/cm^−1^): 3081 (CH_arom_), 2923 (CH_aliph_), 1666 (C=O). ^1^H-NMR (400 MHz, DMSO-*d*_6_) *δ* (ppm): 8.33–8.05 (m, 4H, 4-nitrophenyl ring), 7.43–6.95 (m, 3H, thienyl ring), 5.94, 5.91 (dd, 1H, (**CH**)pyrazole), 3.93, 3.89, 3.47, 3.42 (2 dd, 2H, (**CH**_2_)pyrazole), 2.31 (s, 3H, CH_3_). MS (*m*/*z*, %): 317 (1.09), 315 (M˙^+^; 34.28).

#### 4.1.4. Synthesis of 3-(4-nitrophenyl)-5-(thiophen-2-yl)-4,5-dihydro-1*H*-pyrazole-1-carbaldehyde (4)

Compound 1 (10 mmol, 2.59 g) and hydrazine hydrate (20 mmol, 1 mL) were refluxed for five hours with formic acid (10 mL) present (TLC monitored). Compound 4 was obtained by cooling the reaction mixture, pouring it onto crushed ice, filtering the precipitate, washing it with water, drying it, and recrystallizing it from 1,4-dioxane. Yield 70%; pale green powder; m.p. 205–207 °C. FT-IR (KBr, *ν*/cm^−1^): 3064 (CH_arom_), 2901 (CH_aliph_), 1651 (C=O). ^1^H-NMR (400 MHz, DMSO-*d*_6_) *δ* (ppm): 8.93 (s, 1H, –CHO), 8.34–8.05 (m, 4H, 4-nitrophenyl ring), 7.47–6.97 (m, 3H, thienyl ring), 5.94, 5.91 (dd, 1H, (**CH**)pyrazole), 4.01, 3.96, 3.52, 3.48 (2 dd, 2H, (**CH**_2_)pyrazole).

#### 4.1.5. Synthesis of 4-(4-nitrophenyl)-2-(thiophen-2-yl)-2,3-dihydro-1*H*-benzo[*b*][1,4] diazepine (5)

Compound 1 (10 mmol, 2.59 g) in glacial acetic acid (10 mL) was mixed with a solution of *o*-phenylenediamine (10 mmol, 1.08 g) in absolute ethanol (5 mL) and refluxed. TLC was used to track the reaction's progress. The process was completed, and the solvent had totally evaporated after nine hours. The precipitate was gathered, cleaned with 10 milliliters of petroleum ether at 40 to 60 degrees Celsius, dried, and then recrystallized from ethanol. Yield 46%; pale yellow crystals; m.p. 151–154 °C. FT-IR (KBr, *ν*/cm^−1^): 3111 (NH), 1676 (C=N). ^1^H-NMR (400 MHz, DMSO-*d*_6_) *δ* (ppm): 8.92 (s, 1H, NH, exchangeable with D_2_O), 8.30–6.13 (m, 11H, Ar–H), 6.18, 6.14 (dd, 1H, (**CH**)pyrazole), 4.02, 3.96, 3.52, 3.46 (2 dd, 2H, (**CH**_2_)pyrazole). MS (*m*/*z*, %): 351 (1.82), 349 (M˙^+^; 41.42).

#### 4.1.6. Synthesis of 6-(4-nitrophenyl)-2-oxo-4-(thiophen-2-yl)-1,2-dihydropyridine-3-carbonitrile (6)

Compound 1 (10 mmol, 2.59 g), ethyl cyanoacetate (10 mmol, 1.1 mL), and ammonium acetate (10 mmol, 0.8 g) were refluxed for 11 hours in ethanol (20 mL). The reaction mixture was cooled and then mixed with 50 milliliters of ice-cold water. The precipitate was filtered and recrystallized from ethanol in order to extract compound 6 as a brown powder. Yield 57%, m.p. 194–197 °C. FT-IR (KBr, *ν*/cm^−1^): 3363 (NH), 2214 (C≡N), 1650 (C=O). ^1^H-NMR (400 MHz, DMSO-*d*_6_) *δ* (ppm): 12.35 (s, 1H, NH, exchangeable with D_2_O), 8.63–7.36 (m, 3H, thienyl ring), 8.42–8.29 (m, 4H, 4-nitrophenyl ring), 6.35 (s, 1H, (**CH**)pyridone). MS (*m*/*z*, %): 325 (1.70), 323 (M˙^+^; 37.03).

#### 4.1.7. Synthesis of 2-chloro-1-(3-(4-nitrophenyl)-5-(thiophen-2-yl)-4,5-dihydro-1*H*-pyrazol-1-yl)ethan-1-one (7)

Compound 2 (10 mmol, 2.73 g) was dissolved in 20 mL of dioxane while a catalytic quantity of triethylamine (TEA) was present. After that, the solution was refluxed for eight hours while dropwise additions of chloroacetyl chloride (10 mmol, 0.8 mL) were made. The reaction mixture was cooled, and then added to 50 milliliters of ice-cold water. Compound 7 was obtained as a yellow powder by filtering, washing, and recrystallizing the resultant precipitate from ethanol in 48% yield, m.p. 138–140 °C. FT-IR (KBr, *ν*/cm^−1^): 3089 (CH_arom_), 2984, 2929 (CH_aliph_), 1689 (C=O). ^1^H-NMR (400 MHz, DMSO-*d*_6_) *δ* (ppm): 8.31–8.01 (m, 4H, 4-nitrophenyl ring), 7.45–6.97 (m, 3H, thienyl ring), 5.85–5.23 (m, 1H, (**CH**)pyrazole), 4.15 (d, 2H, CH_2_Cl), 3.94, 3.90, 3.44, 3.40 (2 dd, 2H, (**CH**_2_)pyrazole). ^13^C-NMR (100 MHz, DMSO-*d*_6_) *δ* (ppm): 164.19 (C=O), 154.58, 148.79, 143.93, 137.07, 128.59, 127.29, 126, 125.64, 124.45, 56.66 ((**CH**_2_)pyrazole), 42.85 (CH_2_Cl), 41.95 (**CH**-pyrazole).

#### 4.1.8. Synthesis of 2-(3-(4-nitrophenyl)-5-(thiophen-2-yl)-4,5-dihydro-1*H*-pyrazole-1-carbonyl)benzoic acid (8)

Pyrazole derivative 2 (10 mmol, 2.73 g) and phthalic anhydride (10 mmol, 1.48 g) were refluxed for nine hours in glacial acetic acid (10 mL). The reaction's development was tracked using TLC. Once the finished mixture had cooled, it was poured into a beaker containing ice-cold water. The precipitated solid was collected by filtration, and the pure product was obtained by washing the solid with water, drying it, and recrystallizing it from ethanol. The powder of the obtained compound 8 was brown in color with a m.p. of 185–188 °C and a 57% yield. FT-IR (KBr, *ν*/cm^−1^): 3418 (OH), 1694 (C=O)acid, 1665 (C=O)amide. ^1^H-NMR (400 MHz, DMSO-*d*_6_) *δ* (ppm): 13.14 (s, 1H, OH, exchangeable with D_2_O), 8.26–7.80 (m, 4H, 4-nitrophenyl ring), 7.94–7.22 (m, 4H, phenyl ring), 7.48–7.00 (m, 3H, thienyl ring), 6.09, 6.06 (dd, 1H, (**CH**)pyrazole), 3.97, 3.93, 3.51, 3.47 (2 dd, 2H, (**CH**_2_)pyrazole). MS (*m*/*z*, %): 423 (1.36), 421 (M˙^+^; 39.73). HPLC purity (C18, ACN/H_2_O/MeOH 30/65/5, 1.0 mL min^−1^, UV 250 nm, 25 μL; isocratic): RT = 8 min, % Purity = 98.16%.

#### 4.1.9. Synthesis of 4-(3-(4-nitrophenyl)-5-(thiophen-2-yl)-4,5-dihydro-1*H*-pyrazol-1-yl)-4-oxobutanoic acid (9)

A mixture of pyrazole derivative 2 (10 mmol, 2.73 g) and succinic anhydride (10 mmol, 1.00 g) in glacial acetic acid (10 mL) was refluxed for eight hours. TLC tracked the course of the reaction. After cooling, the finished solution was moved to a beaker containing ice-cold water. Pure product 9, a pale-yellow powder, was obtained by filtering the precipitated solid, washing it with water, drying it, and then recrystallizing it from ethanol with a yield of 54% and a m.p. of 128–130 °C. FT-IR (KBr, *ν*/cm^−1^): 3438 (OH), 1729 (C=O)acid, 1643 (C=O)amide. ^1^H-NMR (500 MHz, DMSO-*d*_6_) *δ* (ppm): 12.19 (s, 1H, OH, exchangeable with D_2_O), 8.33–8.06 (m, 4H, 4-nitrophenyl ring), 7.42–6.95 (m, 3H, thienyl ring), 5.92, 5.90 (dd, 1H, (**CH**)pyrazole), 3.94, 3.90, 3.45, 3.42 (2 dd, 2H, (**CH**_2_)pyrazole), 2.96 (t, 4H, 2 CH_2_). ^13^C-NMR (100 MHz, DMSO-*d*_6_) *δ* (ppm): 174.16 (C=O)acid, 169.94 (C=O)amide, 153.20, 148.54, 144.82, 137.55, 128.27, 127.21, 125.64, 125.15, 124.46, 56.25 ((**CH**)pyrazole), 41.86 ((**CH**_2_)pyrazole), 28.83 (CH_2_), 29.35 (CH_2_). MS (*m*/*z*, %): 375 (0.52), 373 (M˙^+^; 13.65).

#### 4.1.10. Synthesis of 4-(3-(4-nitrophenyl)-5-(thiophen-2-yl)-4,5-dihydro-1*H*-pyrazol-1-yl)-4-oxobut-2-enoic acid (10)

Glacial acetic acid (10 mL) was used to dissolve maleic anhydride (10 mmol, 0.98 g) and pyrazole derivative 2 (10 mmol, 2.73 g). The reaction was then refluxed for eight hours while TLC tracked its development. The resultant solid was filtered, cleaned with water, dried, and recrystallized from ethanol to obtain the pure product 10 after the mixture had cooled and been placed into ice-cold water. It is a pale-green powder; m.p.: 195–198 °C, yield: 56%. FT-IR (KBr, *ν*/cm^−1^): 3417 (OH), 1715 (C=O)acid, 1622 (C=O)amide. ^1^H-NMR (400 MHz, DMSO-*d*_6_) *δ* (ppm): 12.78 (s, 1H, OH, exchangeable with D_2_O), 8.32–8.02 (m, 4H, 4-nitrophenyl ring), 7.45–6.95 (m, 3H, thienyl ring), 6.91 (d, 1H, =CH), 6.35 (d, 1H, =CH), 5.98, 5.96 (dd, 1H, (**CH**)pyrazole), 3.97, 3.93, 3.50, 3.45 (2 dd, 2H, (**CH**_2_)pyrazole).

#### 4.1.11. Synthesis of bis(3-(4-nitrophenyl)-5-(thiophen-2-yl)-4,5-dihydro-1*H*-pyrazol-1-yl)methane (11)

##### 4.1.11.1. Method (A)

After dissolving pyrazole derivative 2 (10 mmol, 2.73 g) in 10 mL of ethanol, formaldehyde (10 mmol, 0.3 mL) was added and stirred for 30 minutes at room temperature. The reaction mixture was then continuously stirred overnight at room temperature while a dropwise addition of an ethanolic solution of *p*-anisidine (10 mmol, 1.23 g) was made. After filtering, drying, and recrystallizing the resultant product 11 from ethanol, a yellow powder was obtained.

##### 4.1.11.2. Method (B)

Pyrazole derivative 2 (10 mmol, 2.73 g) was dissolved in 10 mL of ethanol without the addition of the ethanolic solution of *p*-anisidine. Formaldehyde (10 mmol, 0.3 mL) was then added to the solution while stirring for 30 minutes at room temperature. The product 11 was filtered, dried, and recrystallized. m.p.: 208–210 °C, yield: 47%. FT-IR (KBr, *ν*/cm^−1^): 3110 (CH_arom_), 2949, 2869 (CH_aliph_), 1595 (C=N). ^1^H-NMR (400 MHz, DMSO-*d*_6_) *δ* (ppm): 8.28–6.95 (m, 14H, Ar–H), 5.30, 5.34 (dd, 2H, 2 (**CH**)pyrazole), 4.64 (s, 1H, CH_2_), 3.76–2.97 (m, 4H, 2 (**CH**_2_)pyrazole). MS (*m*/*z*, %): 560 (1.01), 558 (M˙^+^; 21.97).

#### 4.1.12. Synthesis of dimethyl 2-(3-(4-nitrophenyl)-5-(thiophen-2-yl)-4,5-dihydro-1*H*-pyrazol-1-yl)maleate (12)

Pyrazole derivative 2 (10 mmol, 2.73 g) and dimethyl acetylenedicarboxylate (10 mmol, 1.2 mL) were diluted in 15 mL of ethanol and refluxed for one hour. After filtering the resultant precipitate and recrystallizing the crude solid from ethanol, product 12, yellow crystals, was obtained; m.p.: 201–203 °C, yield: 64%. FT-IR (KBr, *ν*/cm^−1^): 1746, 1687 (C=O)ester, 1589 (C=N). ^1^H-NMR (400 MHz, DMSO-*d*_6_) *δ* (ppm): 8.30–7.91 (m, 4H, 4-nitrophenyl ring), 7.53–7.01 (m, 3H, thienyl ring), 6.01, 5.98 (dd, 1H, (**CH**)pyrazole), 4.88 (s, 1H, =CH), 4.08, 4.03, 3.45, 3.41 (2 dd, 2H, (**CH**_2_)pyrazole), 3.86 (s, 3H, CH_3_), 3.52 (s, 3H, CH_3_). MS (*m*/*z*, %): 417 (1.65), 415 (M˙^+^; 47.25).

#### 4.1.13. Synthesis of 3-(4-nitrophenyl)-5-(thiophen-2-yl)-1*H*-pyrazole-1-carboxylic acid (13)

After adding 50% aqueous NaOH solution (5 mL) to a vigorously stirring solution of pyrazole derivative 4 (10 mmol, 3.01 g) in ethanol (20 mL), with or without *p*-nitroacetophenone (10 mmol, 1.65 g), the mixture was left to stir for 10 hours at room temperature. Precoated TLC plates were used to track the reaction's progress. To obtain the pure product as yellow crystals, the mixture was neutralized with 1N hydrochloric acid, and the precipitate that resulted was filtered and recrystallized from ethanol; m.p.: 250–253 °C, yield 58%. FT-IR (KBr, *ν*/cm^−1^): br. 3210 (OH), 1682 (C=O). ^1^H-NMR (400 MHz, DMSO-*d*_6_) *δ* (ppm): 13.69 (s, 1H, OH, exchangeable with D_2_O), 8.41–7.25 (m, 7H, Ar–H), 7.16 (s, 1H, pyrazole ring). ^13^C-NMR (100 MHz, DMSO-*d*_6_) *δ* (ppm): 147.03 (C=O)acid, 135.67, 129.78, 129.47, 128.44, 126.42, 125.46, 124.72, 123.14. MS (*m*/*z*, %): 317 (0.87), 315 (M˙^+^; 15.42).

#### 4.1.14. 4-Acetyl-3-(4-nitrophenyl)-5-(thiophen-2-yl)-4,5-dihydro-1*H*-pyrazole-1-carbaldehyde (14)

Compound 4 (10 mmol, 3.01 g) and acetyl chloride (10 mmol, 0.7 mL) were dissolved in ethanol (10 mL) with a catalytic quantity of triethylamine, and the mixture was refluxed for six hours. TLC tracked the development of the reaction. The reaction mixture was allowed to cool before being poured into ice-cold water. The solid that resulted from this process was then filtered and recrystallized from ethanol to produce pure product 14, which took the form of yellow crystals; m.p.: 270–272 °C, yield 66%. FT-IR (KBr, *ν*/cm^−1^): br 1654 (C=O). ^1^H-NMR (400 MHz, DMSO-*d*_6_) *δ* (ppm): 8.93 (s, 1H, –CHO), 8.33–8.04 (m, 4H, 4-nitrophenyl ring), 7.47–6.97 (m, 3H, thienyl ring), 5.94 (d, 1H, (**CH**)pyrazole), 3.98 (d, 1H, (**CH**)pyrazole), 1.67 (s, 3H, CH_3_). MS (*m*/*z*, %): 345 (0.38), 343 (M˙^+^; 12.04). HPLC purity (C18, ACN/H_2_O/MeOH 30/65/5, 1.0 mL min^−1^, UV 250 nm, 25 μL; isocratic): RT = 6 min, % purity = 99.91%.

### 4.2. Biological assessments

#### 4.2.1. Analysis of cytotoxic inhibitory concentration 50 (IC_50_) with respect to HepG2 and MCF-7

The cytotoxic activity of the synthesized pyrazole–thiophene derivatives was initially evaluated against two standard human cancer cell lines: MCF-7 and HepG2, which are widely used in cytotoxicity screening using the 3-(4,5-dimethylthiazol-2-yl)-2,5-diphenyltetrazolium bromide (MTT) assay (SI data, S1).^81–84^

#### 4.2.2. EGFR enzyme inhibition assay (wild and mutant (T790M) types) and VEGFR-2 enzyme inhibition assay

The EGFR inhibitory activity of the test compounds was assessed against both wild-type and T790M mutant enzymes using commercial assay kits (Cat. #40321 and #40323).^85^ VEGFR-2 inhibition was tested using the HTScan® Tyrosine Kinase Assay (Cat. #7788, Cell Signaling Technology),^86^ (SI data, S2).

#### 4.2.3. Assessment of the impact of compound 14 on cell cycle progression and apoptosis in MCF-7 cells

Flow cytometric assay and the pro-apoptotic activity of compound 14 were assessed using the Annexin V-FITC Apoptosis Detection Kit (BioVision, USA; Cat. No. K101-25). The assay of compound 14 was performed in accordance with the reported procedure,^87,88^ (SI data, S3).

### 4.3. Molecular docking

The frontier analogues (2, 8, and 14) were docked using Discovery Studio,^89^ and their binding interactions within wild EGFR, mutant (T790M) EGFR, and VEGFR-2 receptors were visualized by PyMol.^90^ ChemDraw was used to generate the chemical structures of the compounds as mentioned earlier, which were energy minimized and optimized for partial charges.^91^ The crystal structures of wild-type EGFR, mutant (T790M) EGFR, and VEGFR-2 receptors were retrieved from the Protein Data Bank (PDB) (IDs: 6VHN, 5CAL, and 2OH4), respectively. They were refined, hydrogenated (3D), and energy minimization was performed.^92^ The docking processes were applied, and the co-crystals were inserted as positive ref. 93. On the other side, the docking software was examined for its validity by redocking each co-crystal inside its active pocket.^94^ The small values of RMSDs (<2 Å) described the process validation.^95^

## Author contributions

Supervision and conceptualization: Ahmed A. Al-Karmalawy and Abeer M. El-Naggar; data curation, visualization, methodology, and writing – review & editing: Mohammed N. Sallam, Ahmed A. Al-Karmalawy, Eslam M. Abbass, Samia S. Hawas, Abeer M. El-Naggar, and A. M. A. Hassan.

## Conflicts of interest

None.

## Supplementary Material

RA-015-D5RA06852E-s001

RA-015-D5RA06852E-s002

RA-015-D5RA06852E-s003

## Data Availability

The data supporting this article have been included inside the main manuscript and the supplementary information file (SI). Supplementary information: spectroscopic characterization (FT-IR, ^1^H NMR, ^13^C NMR), mass analysis, and HPLC of the synthesized compounds (1–14) (Fig. S1–S42), biological results (Tables S1–S4), and biological assessments (S1–S3). Curves for the IC_50_ calculations for all analogues (1–14). See DOI: https://doi.org/10.1039/d5ra06852e.
