# Supplementary figures and images for: Design, synthesis, and anticancer evaluation of novel pyrazole–thiophene hybrid derivatives as multitarget inhibitors of wild EGFR, mutant (T790M) EGFR, and VEGFR-2
Source: RSC Adv. 2025 Oct 22;15(47):40078–92. doi: 10.1039/d5ra06852e (PMC12541710; doi:10.1039/d5ra06852e)

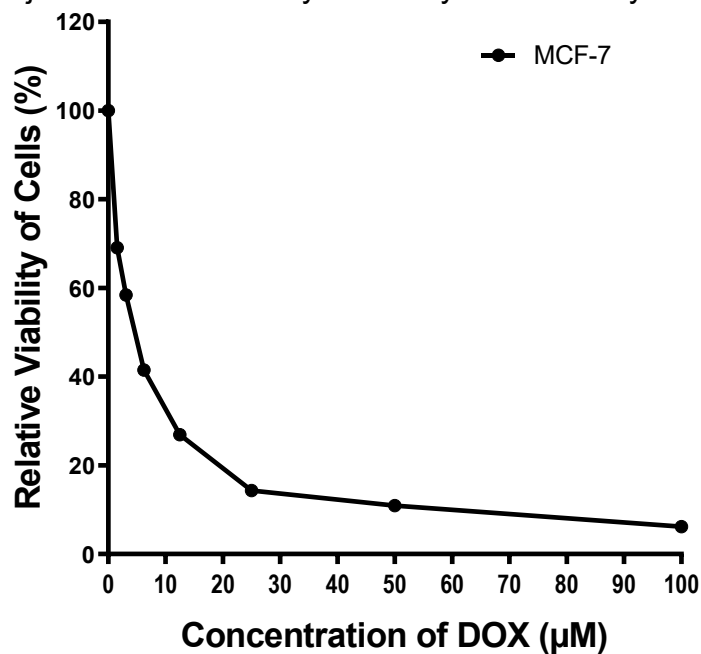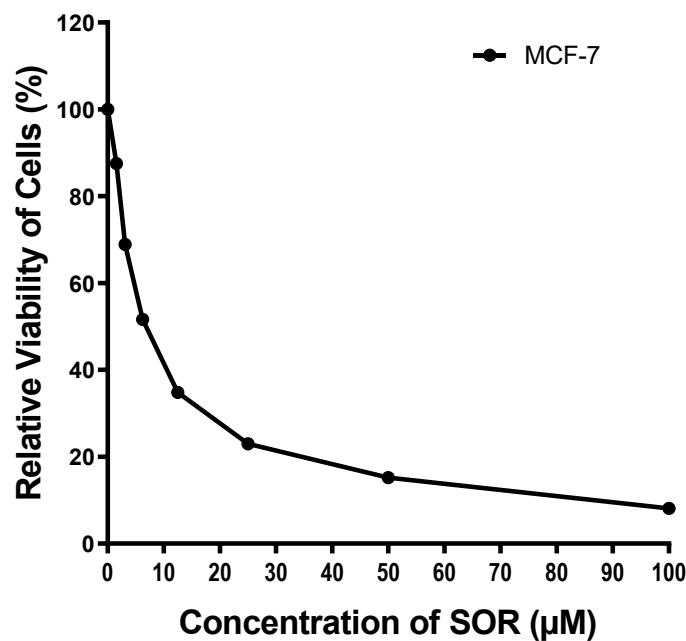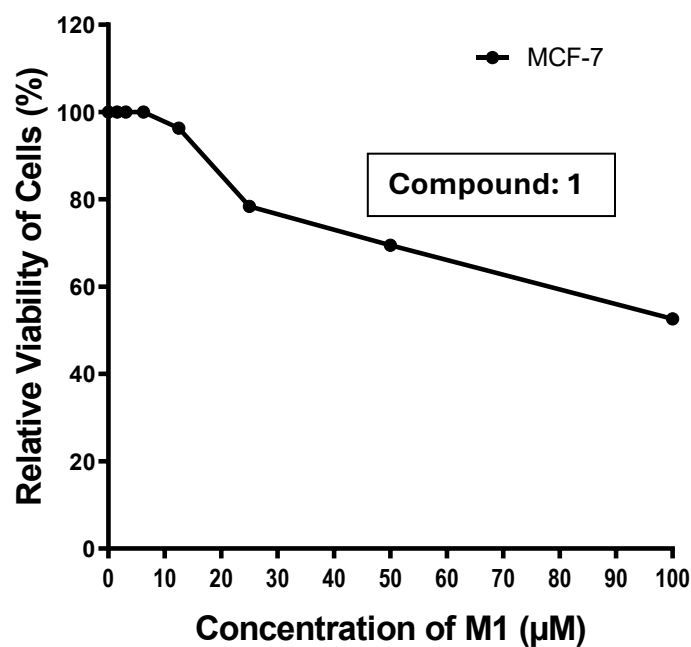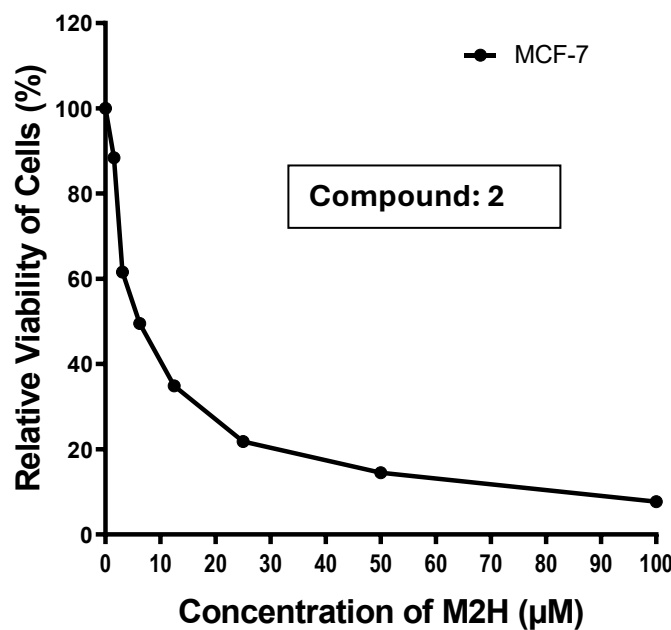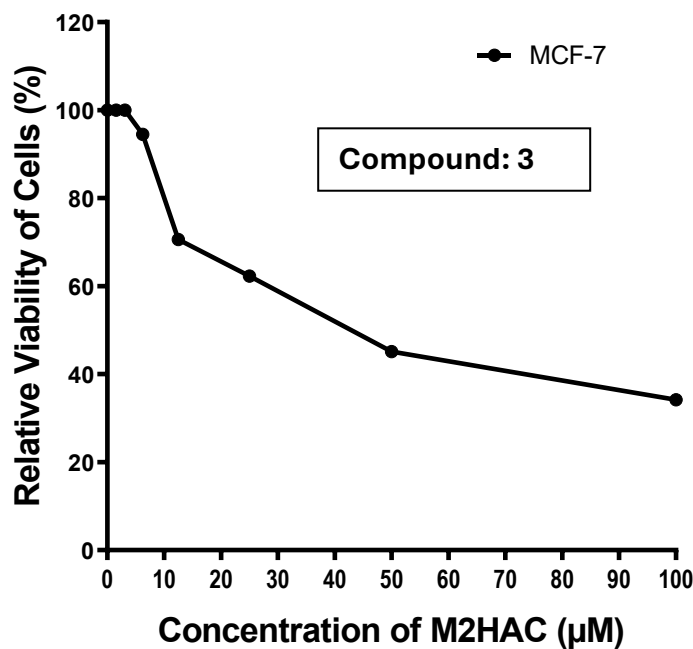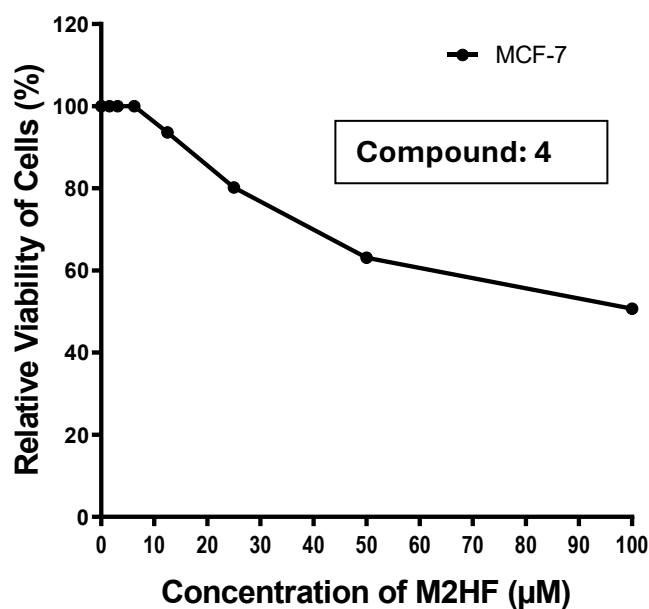

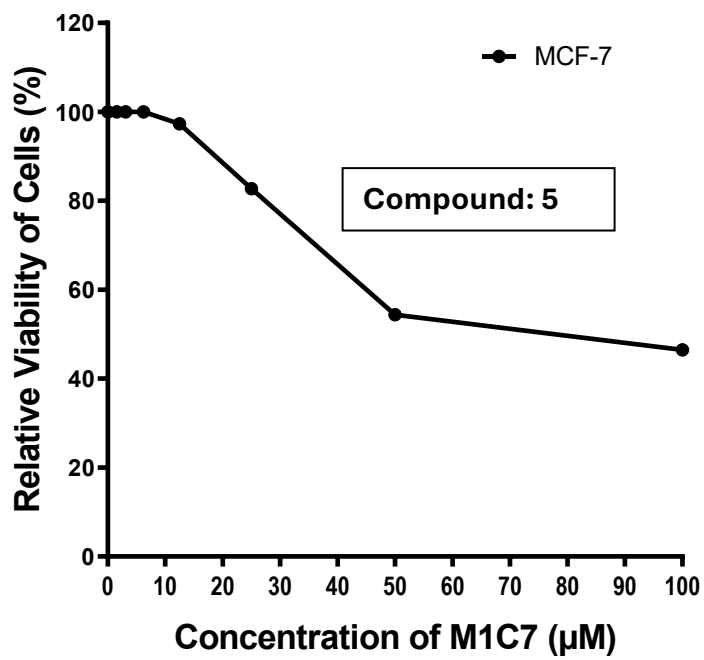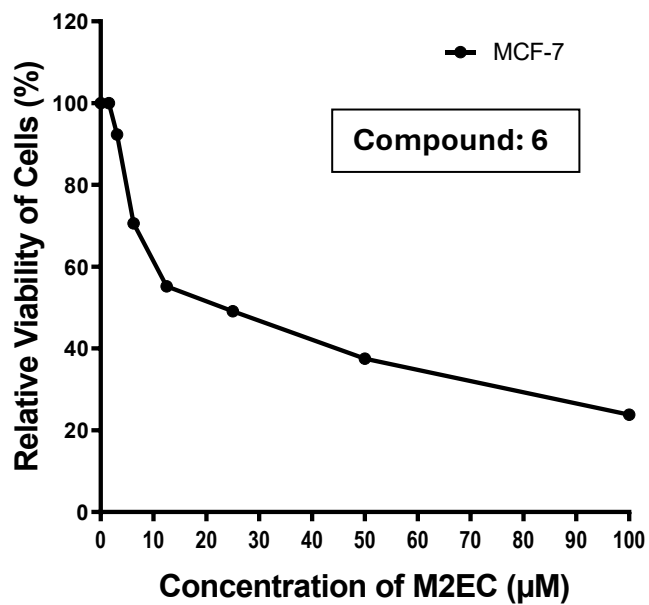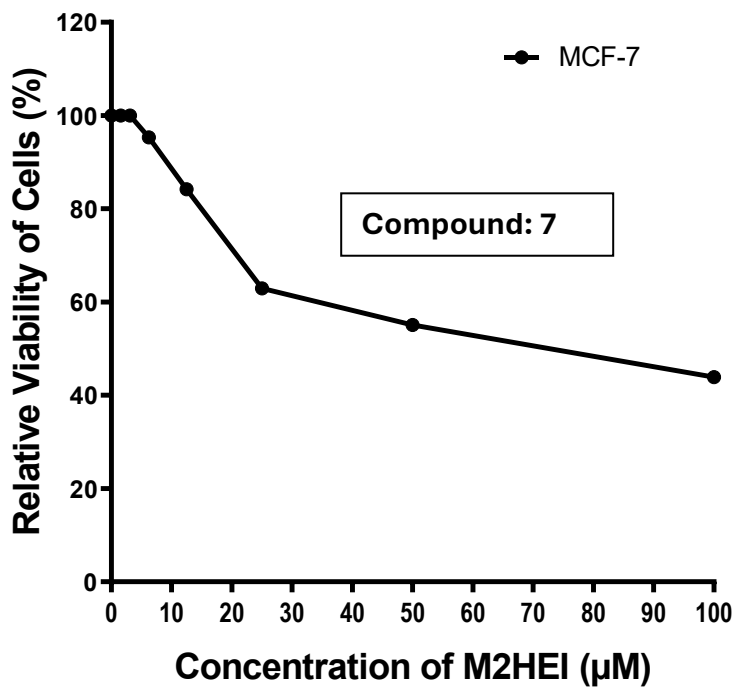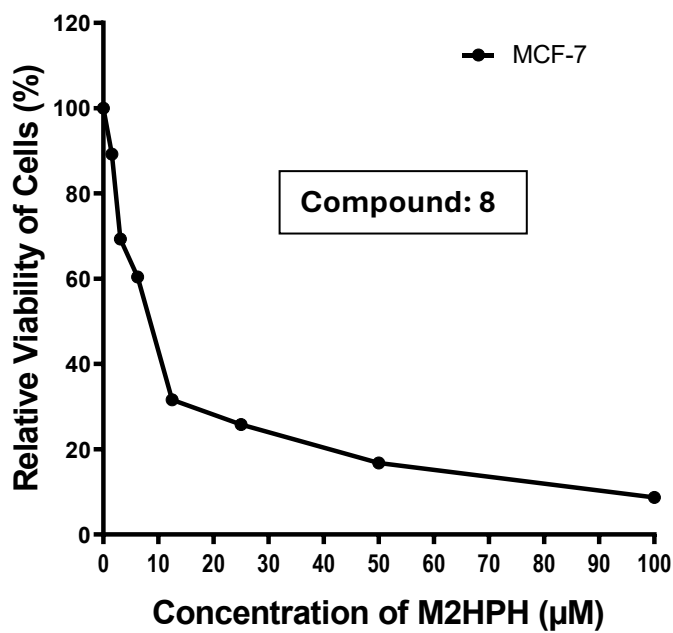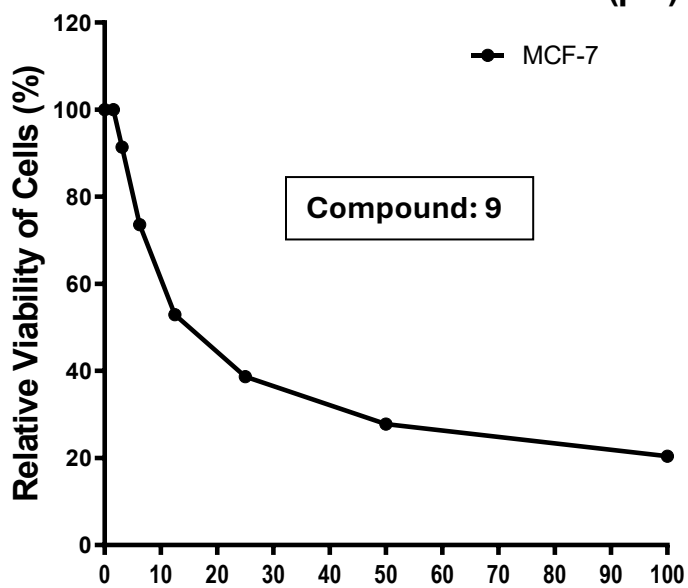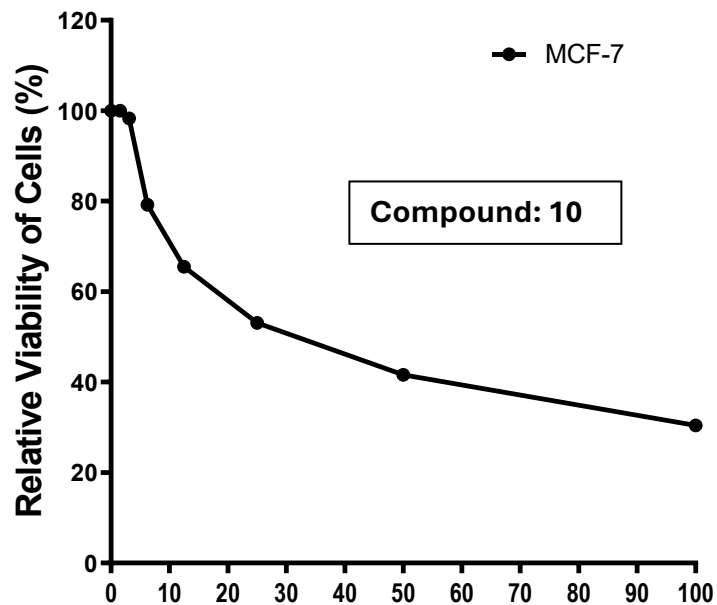

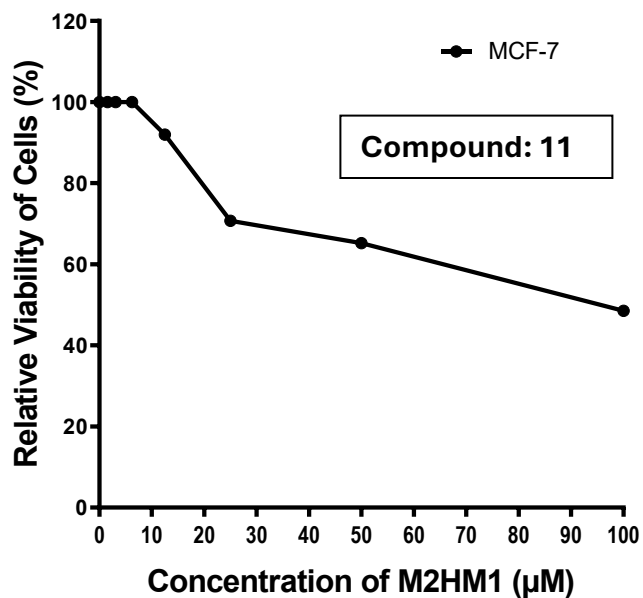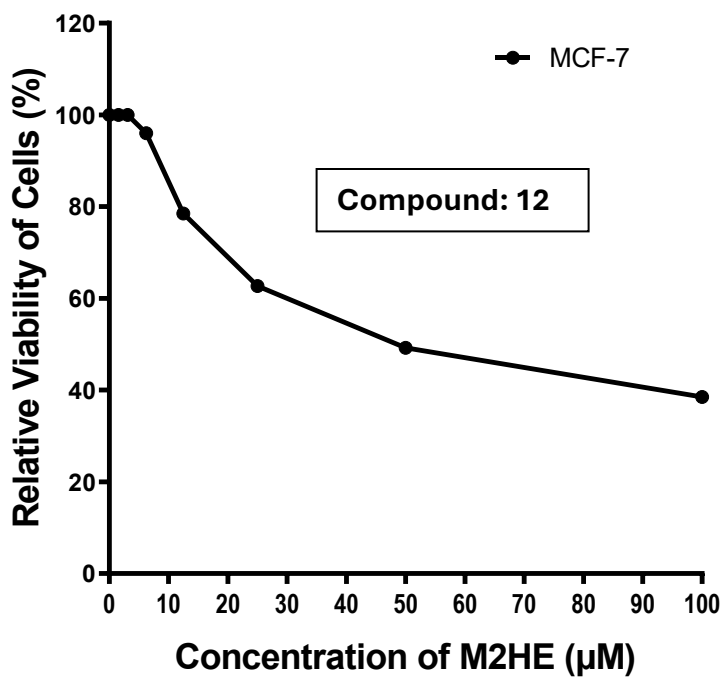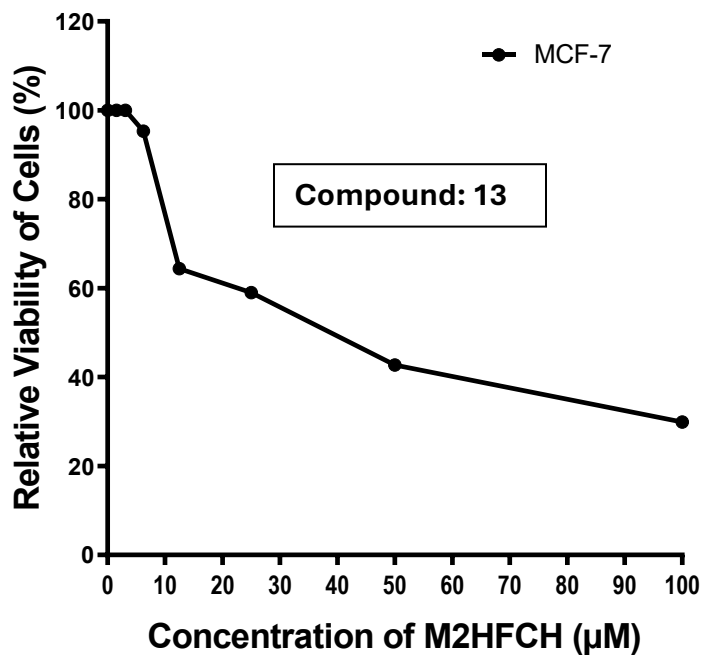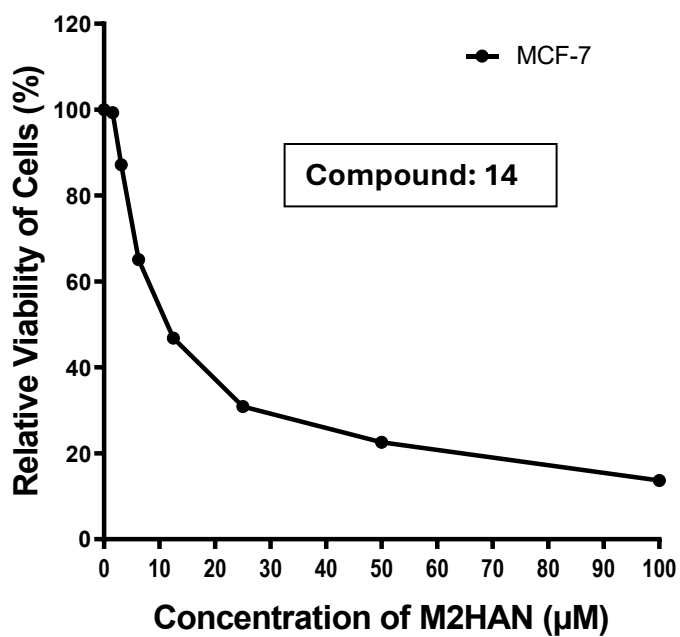

Supplement: RA-015-D5RA06852E-s001 [file RA-015-D5RA06852E-s001.pdf]

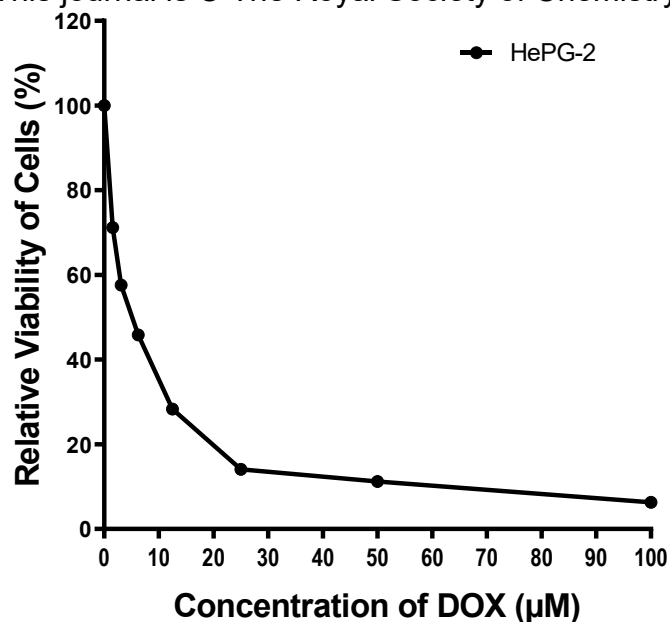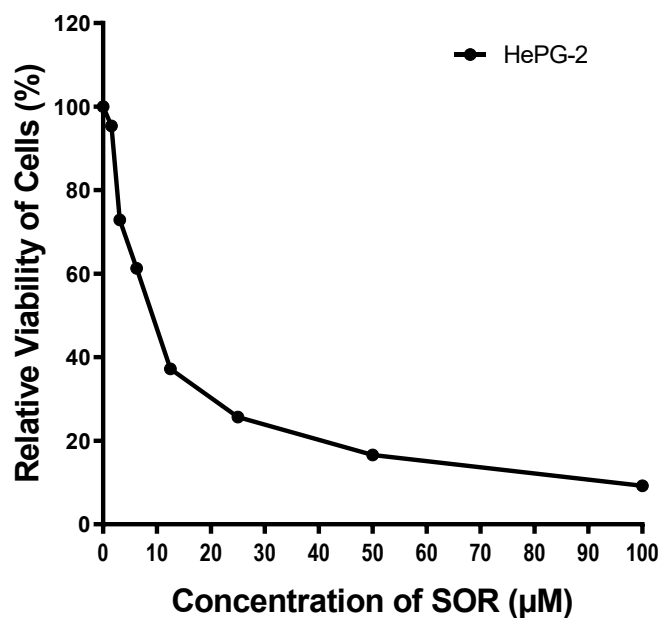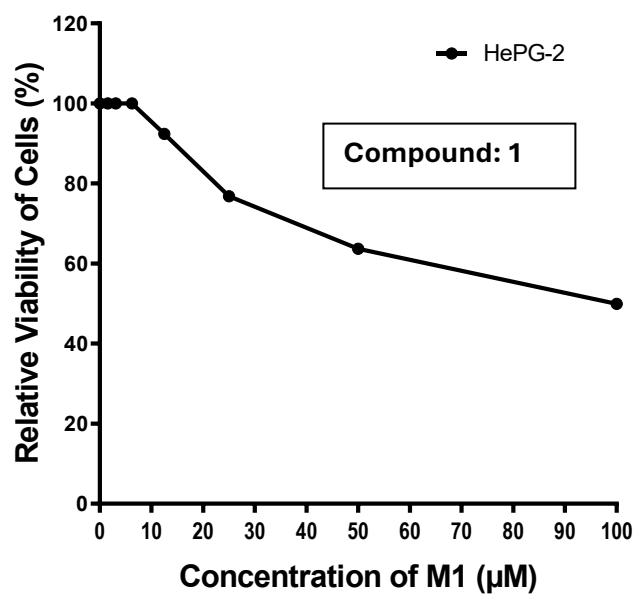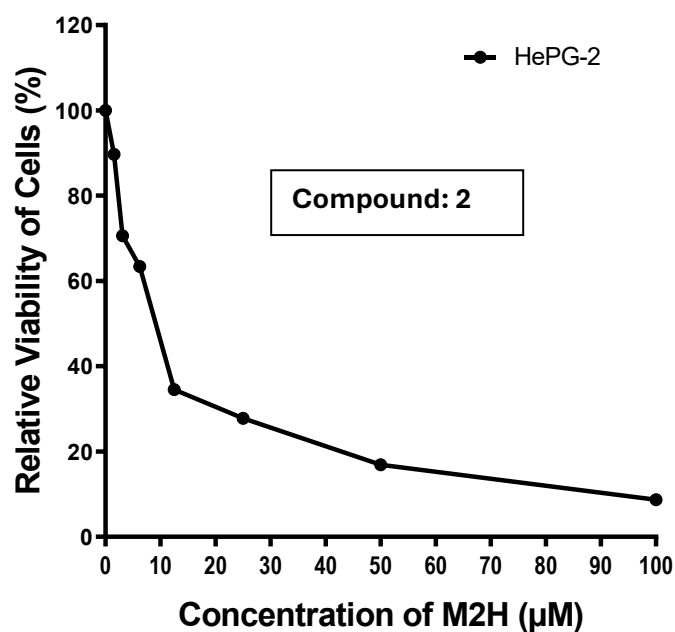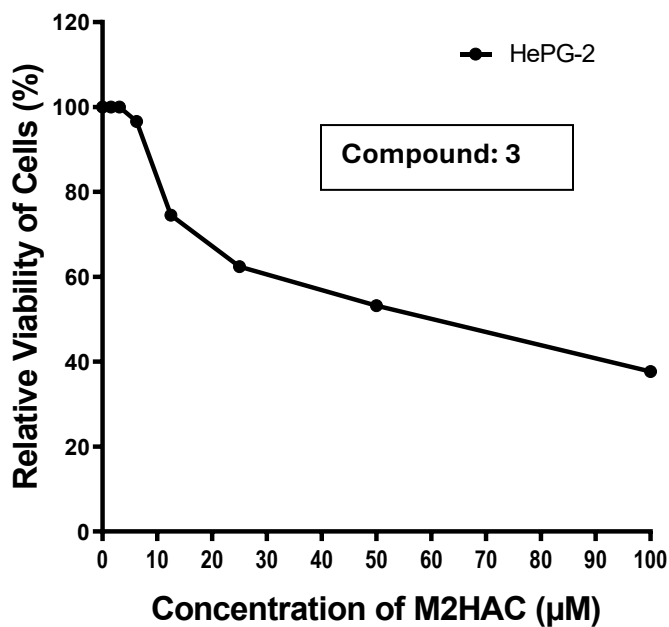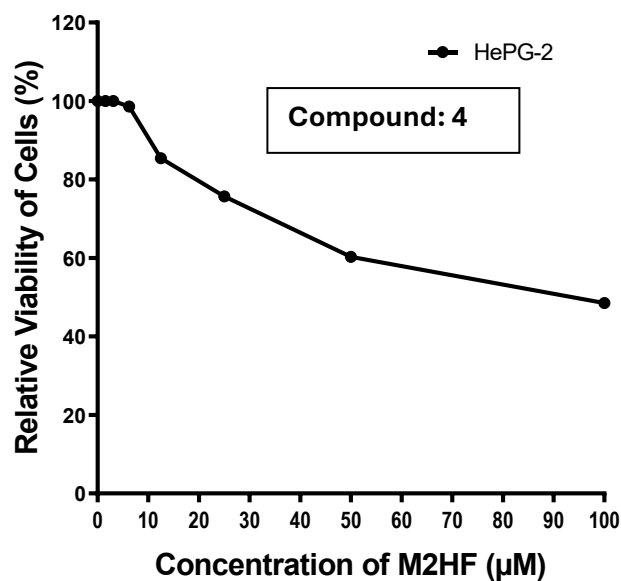

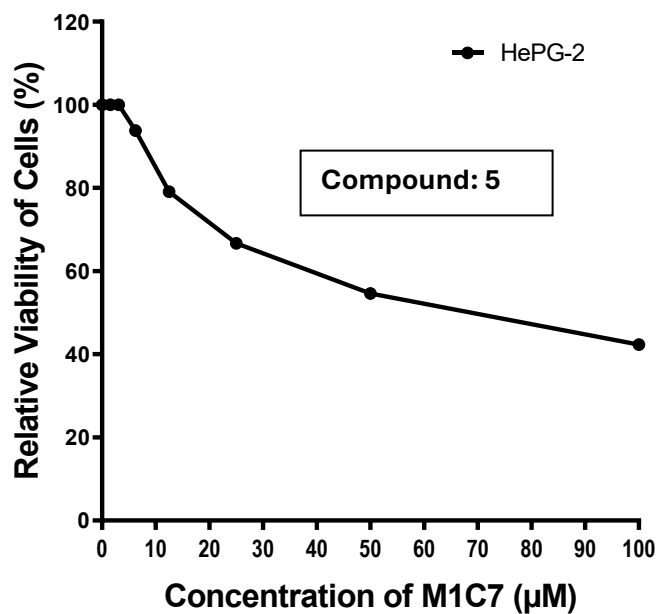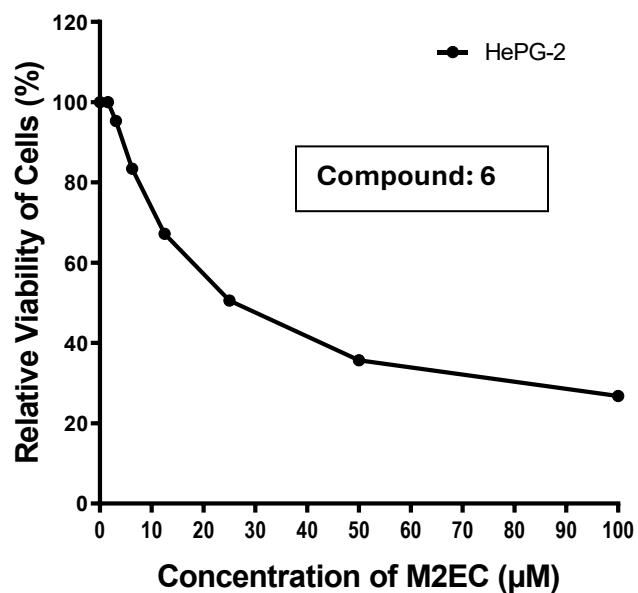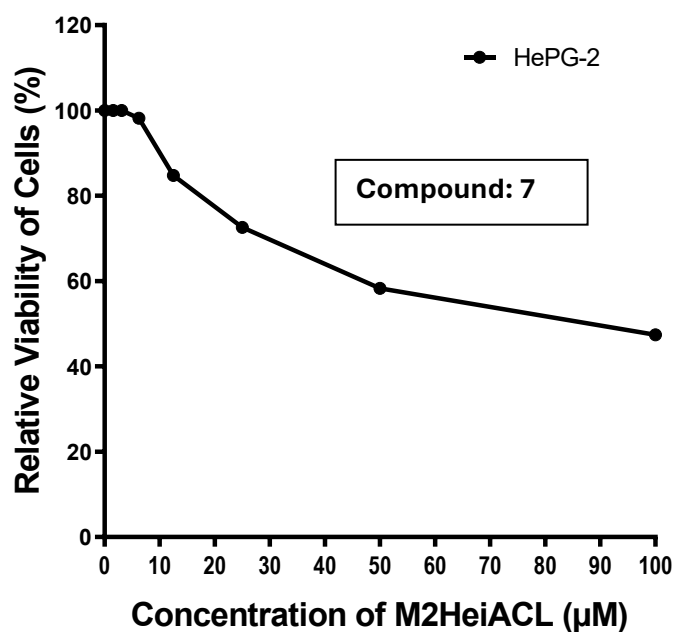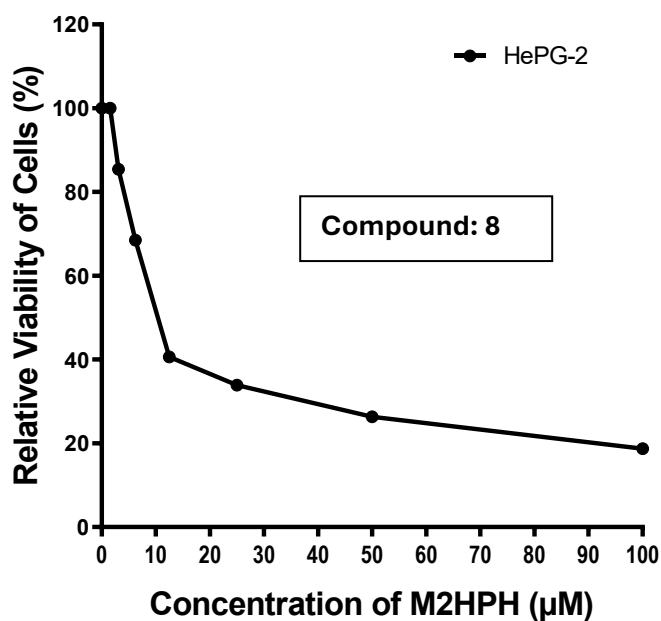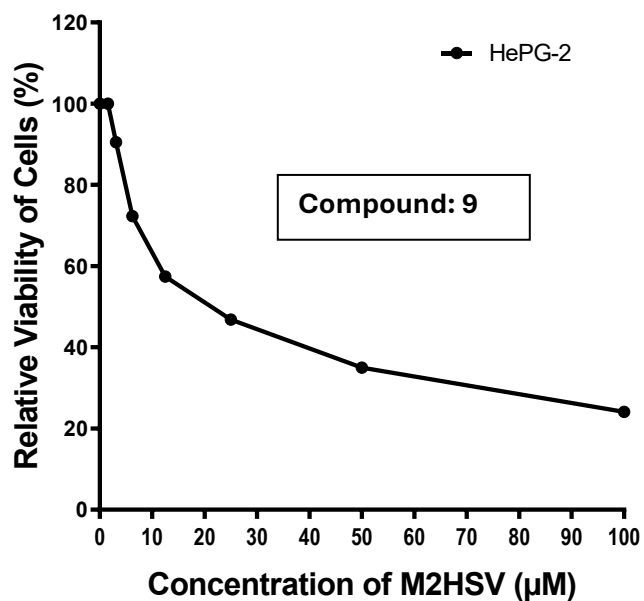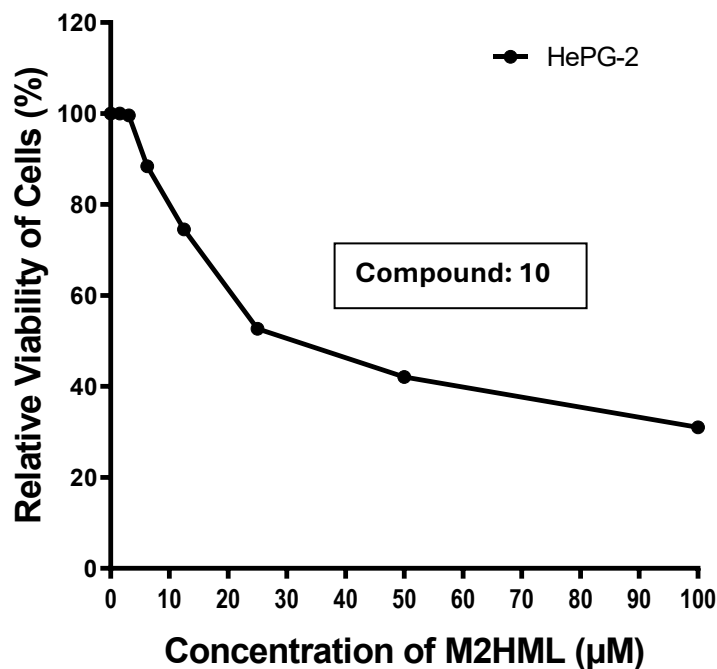

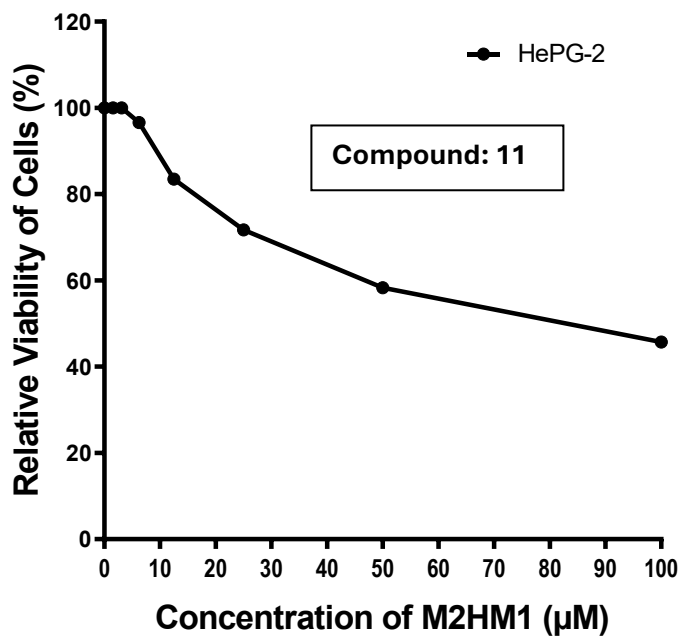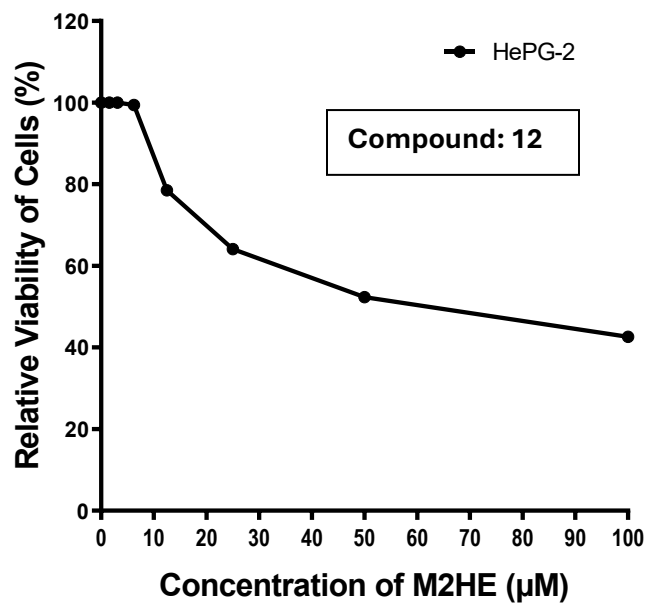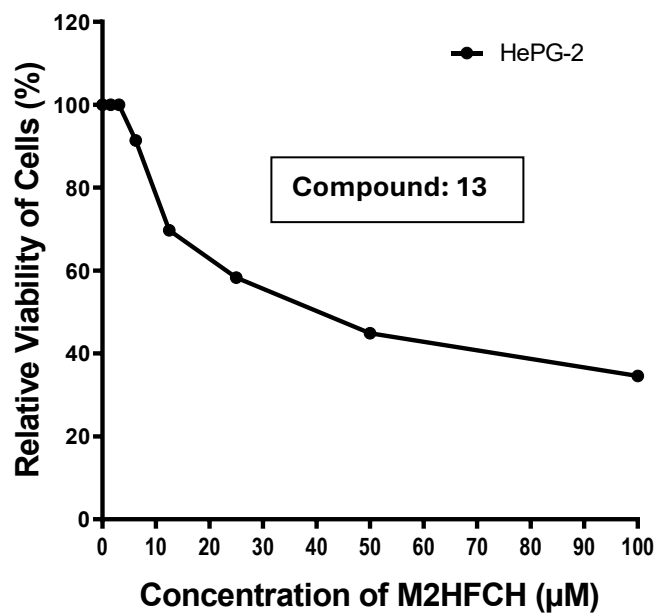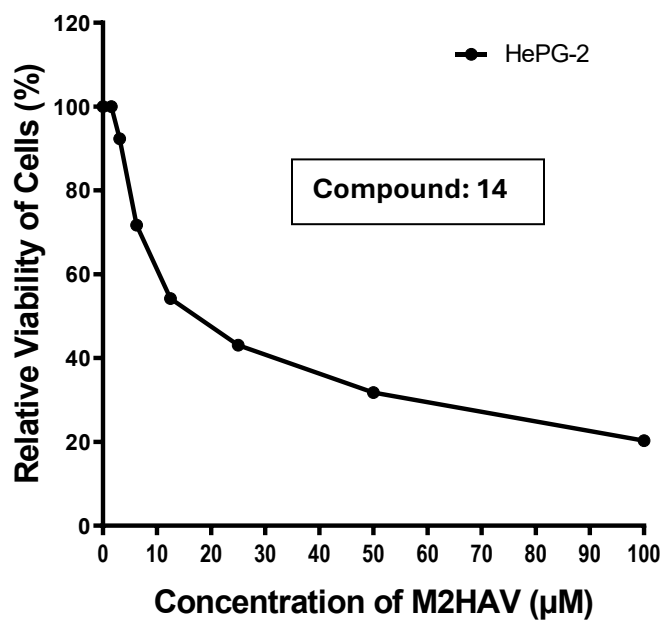

Supplement: RA-015-D5RA06852E-s002 [file RA-015-D5RA06852E-s002.pdf]
