# Supplementary material for: Design, synthesis, and anticancer evaluation of novel pyrazole–thiophene hybrid derivatives as multitarget inhibitors of wild EGFR, mutant (T790M) EGFR, and VEGFR-2
Source: RSC Adv. 2025 Oct 22;15(47):40078–92. doi: 10.1039/d5ra06852e (PMC12541710; doi:10.1039/d5ra06852e)
Supplement: RA-015-D5RA06852E-s003 [file RA-015-D5RA06852E-s003.pdf]

Supplementary Information (SI) for RSC Advances.
This journal is © The Royal Society of Chemistry 2025

Supporting Information (SI)

Design, Synthesis, and Anticancer Evaluation of Novel Pyrazole and Different Heterocyclic Derivatives as Multitarget Inhibitors of Wild EGFR, Mutant (T790M) EGFR, and VEGFR-2

Mohammed N. Sallam[1], Ahmed A. Al-Karmalawy[2,3,*], Eslam M. Abbass[4], Samia S. Hawas[3], Abeer M. El-Naggar[4], A. M. A. Hassan[4]

[1] Egypt Otsuka Pharmaceutical, Tenth of Ramadan, Egypt.

[2] Department of Pharmaceutical Chemistry, College of Pharmacy, The University of Mashreq, Baghdad 10023, Iraq.

[3] Department of Pharmaceutical Chemistry, Faculty of Pharmacy, Horus University-Egypt, New Damietta 34518, Egypt.

[4] Department of Chemistry, Faculty of Science, Ain Shams University, Abbassia, 11566, Cairo, Egypt.

* Correspondence:

Ahmed A. Al-Karmalawy: Email: akarmalawy@horus.edu.eg

Supporting Information (SI)

Table of Contents

Title	Page
**FT-IR, ^{1}H NMR, ^{13}C NMR, and mass spectral data of targets (1-14)**	
**Figure S1.** FTIR spectrum of compound **1**	**S3**
**Figure S2.** FTIR spectrum of compound **2**	**S3**
**Figure S3.** ^{1}H NMR (500 MHz, DMSO-d_6) spectrum of compound **2**	**S4**
**Figure S4.** FTIR spectrum of compound **3**	**S4**
**Figure S5.** ^{1}H NMR (400 MHz, DMSO-d_6) spectrum of compound **3**	**S5**
**Figure S6.** Mass spectrum of compound **3**	**S5**
**Figure S7.** FTIR spectrum of compound **4**	**S6**
**Figure S8.** ^{1}H NMR (400 MHz, DMSO-d_6) spectrum of compound **4**	**S6**
**Figure S9.** FTIR spectrum of compound **5**	**S7**
**Figure S10.** ^{1}H NMR (400 MHz, DMSO-d_6) spectrum of compound **5**	**S7**
**Figure S11.** Mass spectrum of compound **5**	**S8**
**Figure S12.** FTIR spectrum of compound **6**	**S8**
**Figure S13.** ^{1}H NMR (400 MHz, DMSO-d_6) spectrum of compound **6**	**S9**
**Figure S14.** Mass spectrum of compound **6**	**S9**
**Figure S15.** FTIR spectrum of compound **7**	**S10**
**Figure S16.** ^{1}H NMR (400 MHz, DMSO-d_6) spectrum of compound **7**	**S10**
**Figure S17.** ^{13}C NMR (100 MHz, DMSO-d_6) spectrum of compound **7**	**S11**
**Figure S18.** FTIR spectrum of compound **8**	**S11**
**Figure S19.** ^{1}H NMR (400 MHz, DMSO-d_6) spectrum of compound **8**	**S12**
**Figure S20.** Mass spectrum of compound **8**	**S12**
**Figure S21.** FTIR spectrum of compound **9**	**S13**
**Figure S22.** ^{1}H NMR (400 MHz, DMSO-d_6) spectrum of compound **9**	**S13**
**Figure S23.** ^{13}C NMR (100 MHz, DMSO-d_6) spectrum of compound **9**	**S14**
**Figure S24.** Mass spectrum of compound **9**	**S14**
**Figure S25.** FTIR spectrum of compound **10**	**S15**
**Figure S26.** ^{1}H NMR (400 MHz, DMSO-d_6) spectrum of compound **10**	**S15**
**Figure S27.** FTIR spectrum of compound **11**	**S16**
**Figure S28.** ^{1}H NMR (400 MHz, DMSO-d_6) spectrum of compound **11**	**S16**
**Figure S29.** Mass spectrum of compound **11**	**S17**
**Figure S30.** FTIR spectrum of compound **12**	**S17**
**Figure S31.** ^{1}H NMR (400 MHz, DMSO-d_6) spectrum of compound **12**	**S18**
**Figure S32.** Mass spectrum of compound **12**	**S18**
**Figure S33.** FTIR spectrum of compound **13**	**S19**
**Figure S34.** ^{1}H NMR (400 MHz, DMSO-d_6) spectrum of compound **13**	**S19**
**Figure S35.** ^{13}C NMR (100 MHz, DMSO-d_6) spectrum of compound **13**	**S20**
**Figure S36.** Mass spectrum of compound **13**	**S20**
**Figure S37.** FTIR spectrum of compound **14**	**S21**
**Figure S38.** ^{1}H NMR (400 MHz, DMSO-d_6) spectrum of compound **14**	**S21**
**Figure S39.** Mass spectrum of compound **14**	**S22**
**HPLC of the most potent candidates (2, 8, and 14)**	**S23**
**Figure S40.** HPLC of compound **2**	**S23**
**Figure S41.** HPLC of compound **8**	**S24**
**Figure S42.** HPLC of compound **14**	**S25**
**Supplementary Tables**	**S26-S27**
**Biological Assessments**	**S28-S29**

FT-IR, ^{1}H-NMR, ^{13}C-NMR, Mass spectral data of targets (1-14)

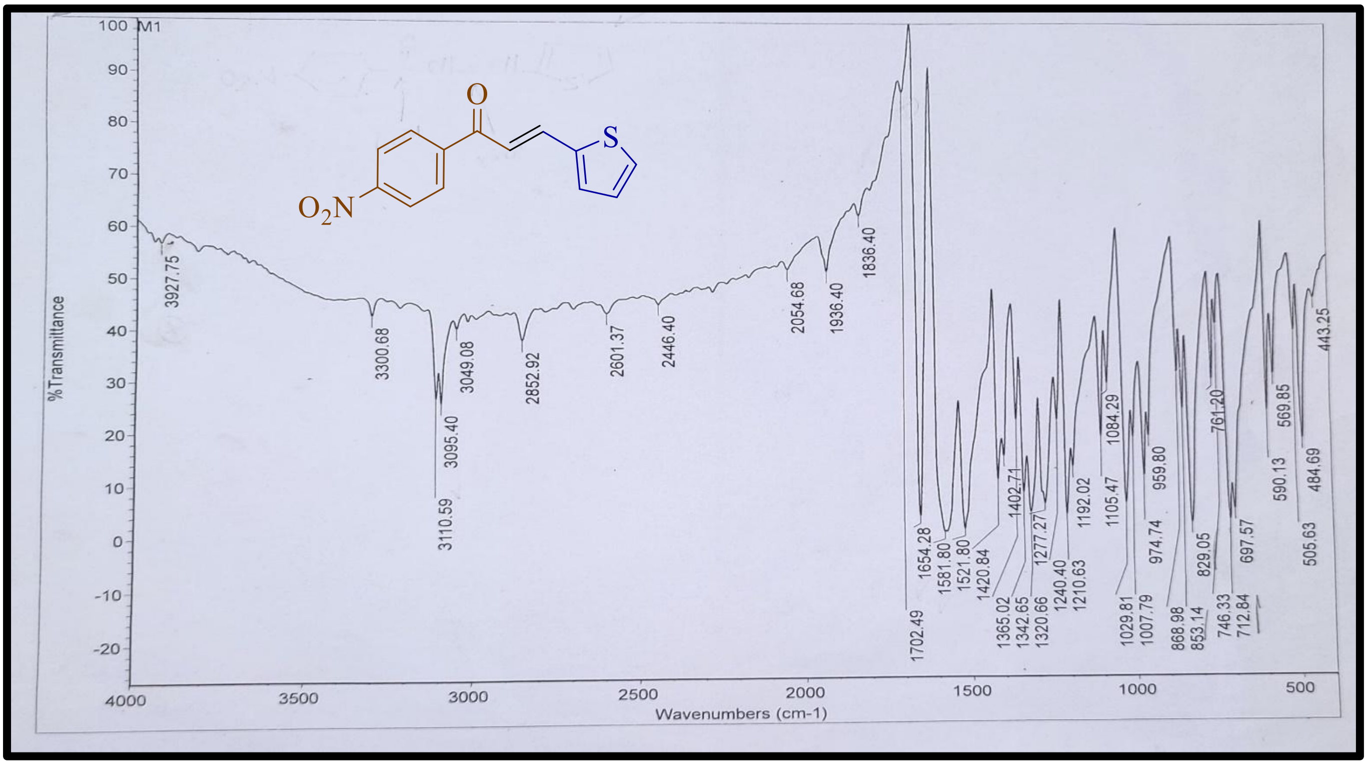

Figure S1. FTIR spectrum of compound **1**

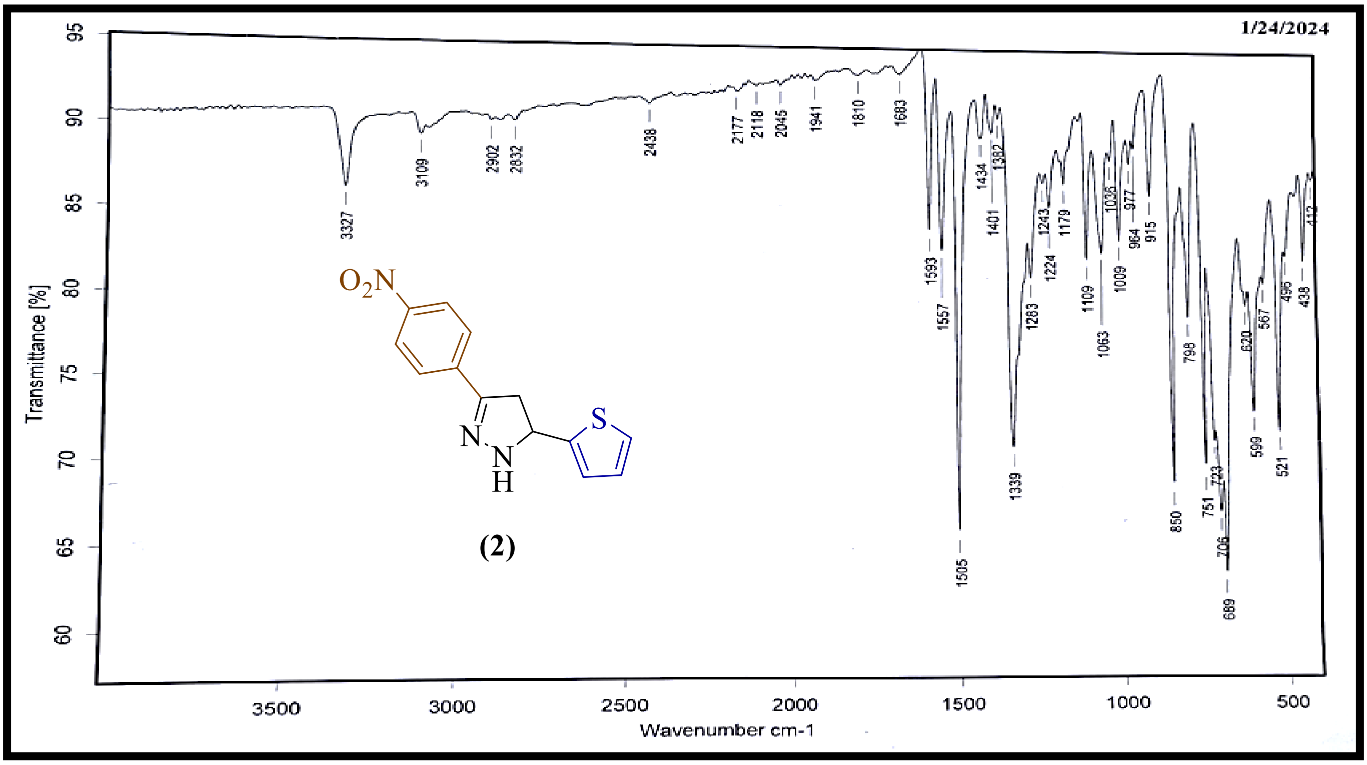

Figure S2. FTIR spectrum of compound **2**

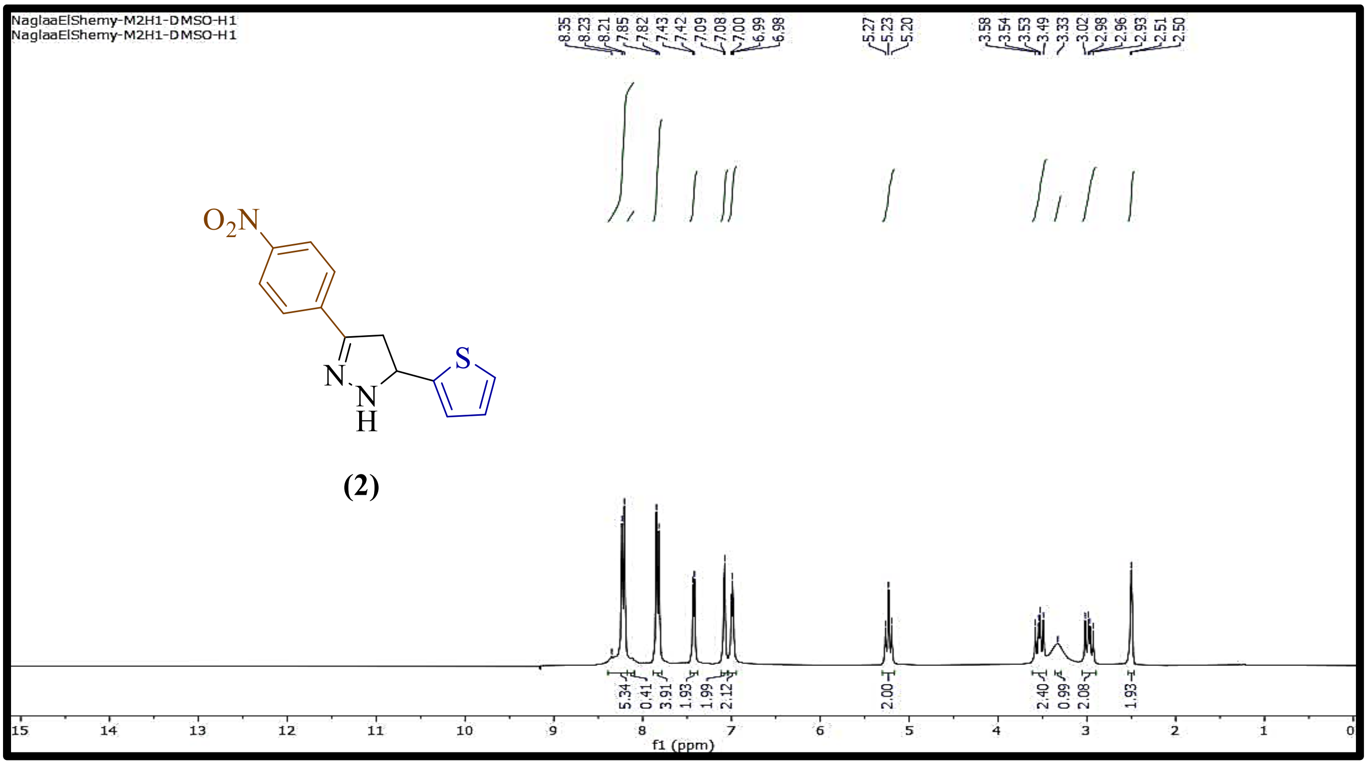

Figure S3. ^{1}H NMR (400 MHz, DMSO-d_6) spectrum of compound **2**

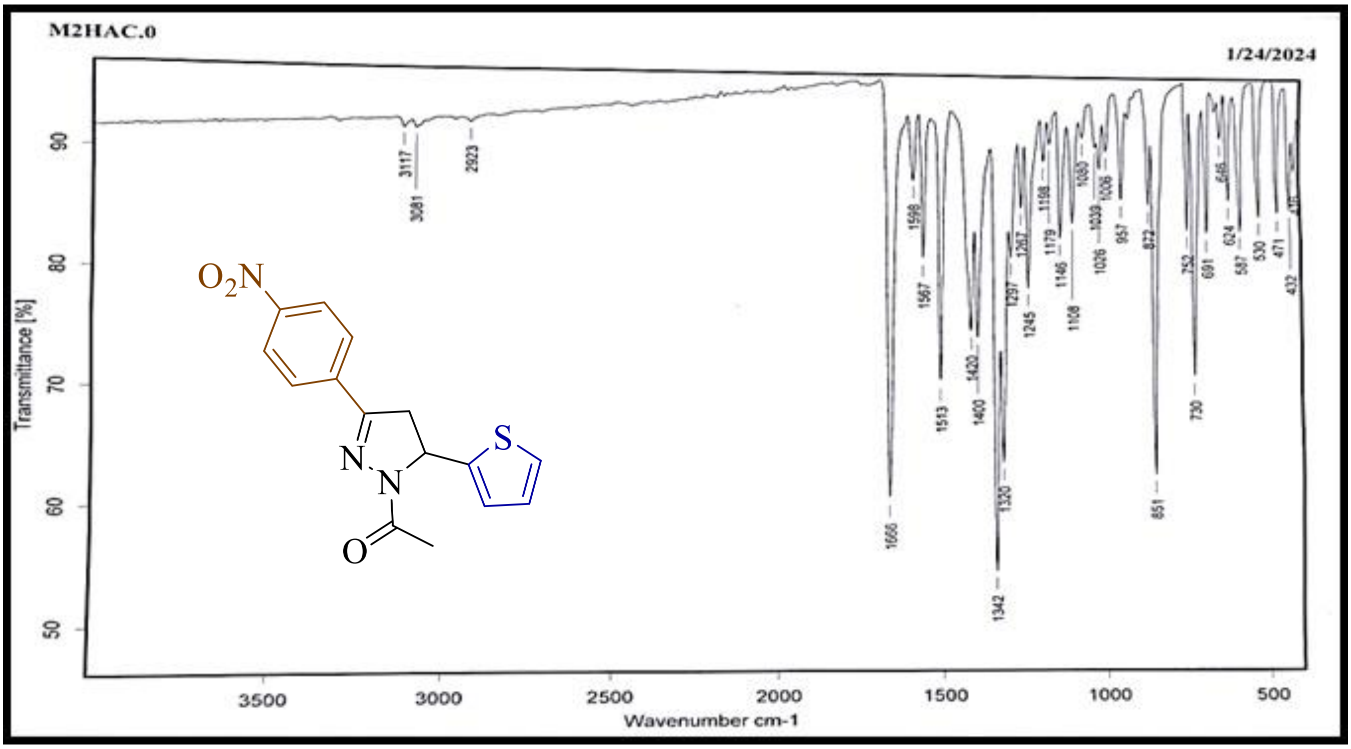

Figure S4. FTIR spectrum of compound **3**

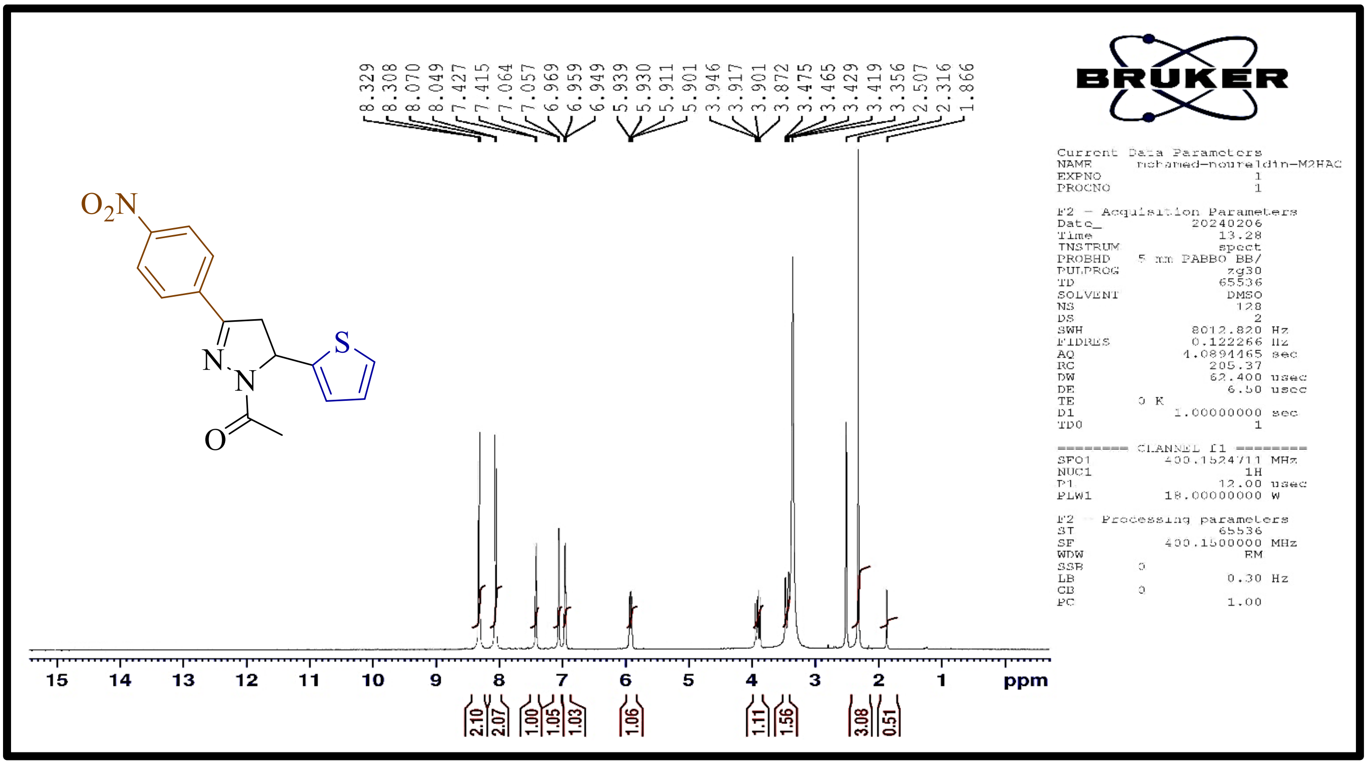

Figure S5. ^{1}H NMR (400 MHz, DMSO-d_6) spectrum of compound **3**

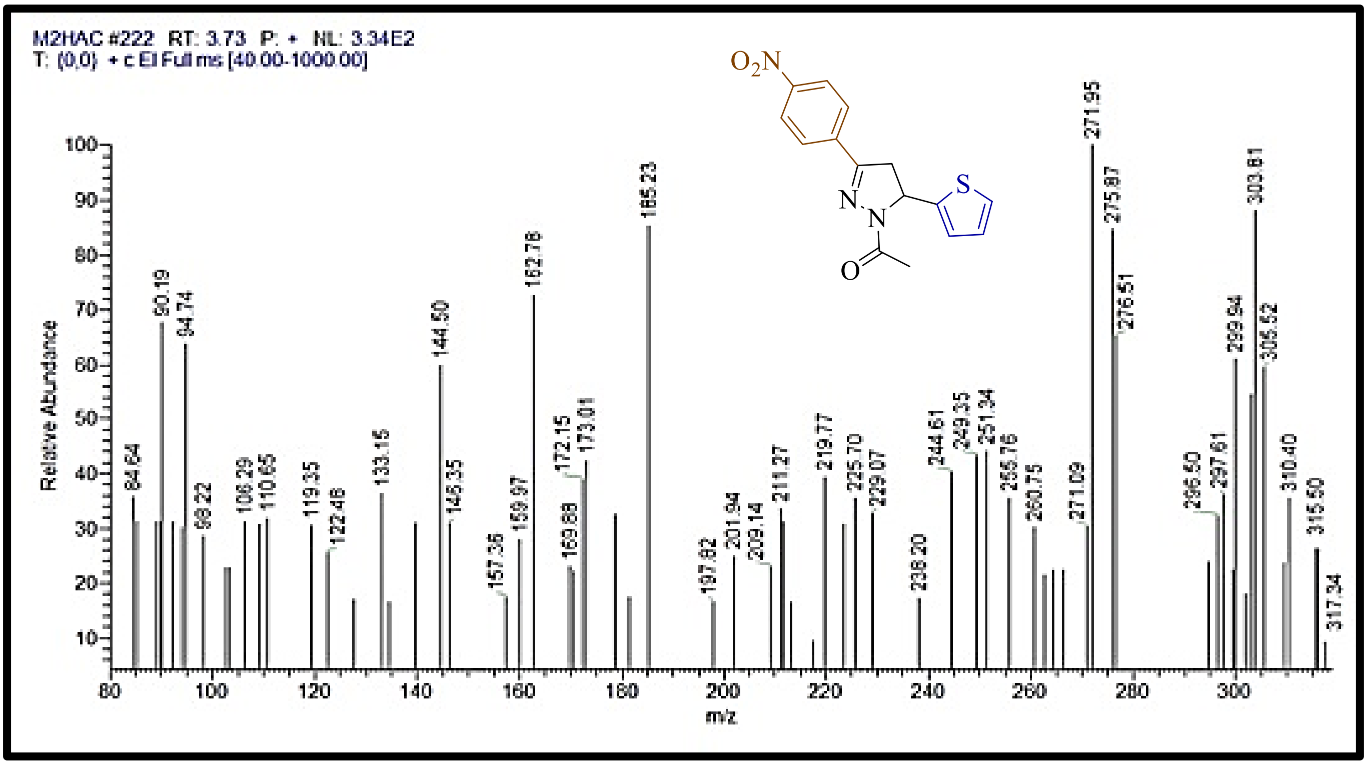

Figure S6. Mass spectrum of compound **3**

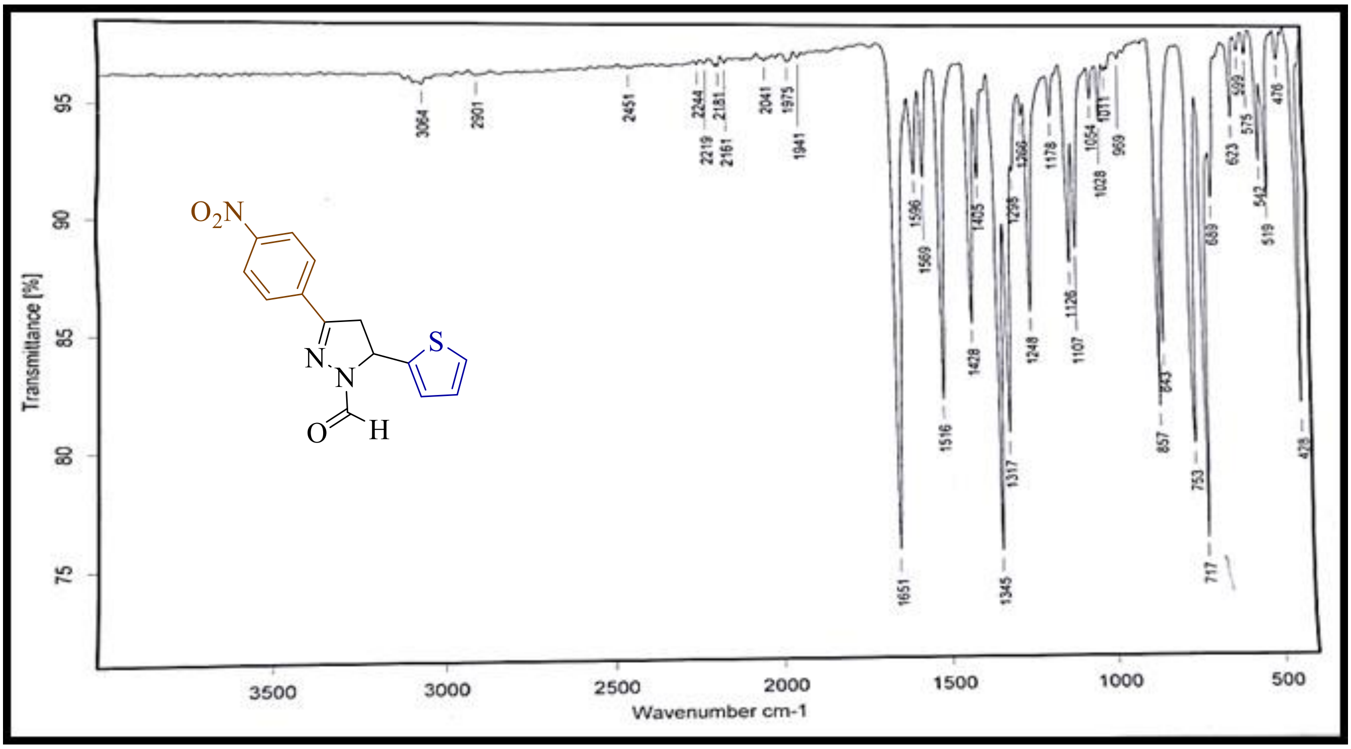

Figure S7. FTIR spectrum of compound **4**

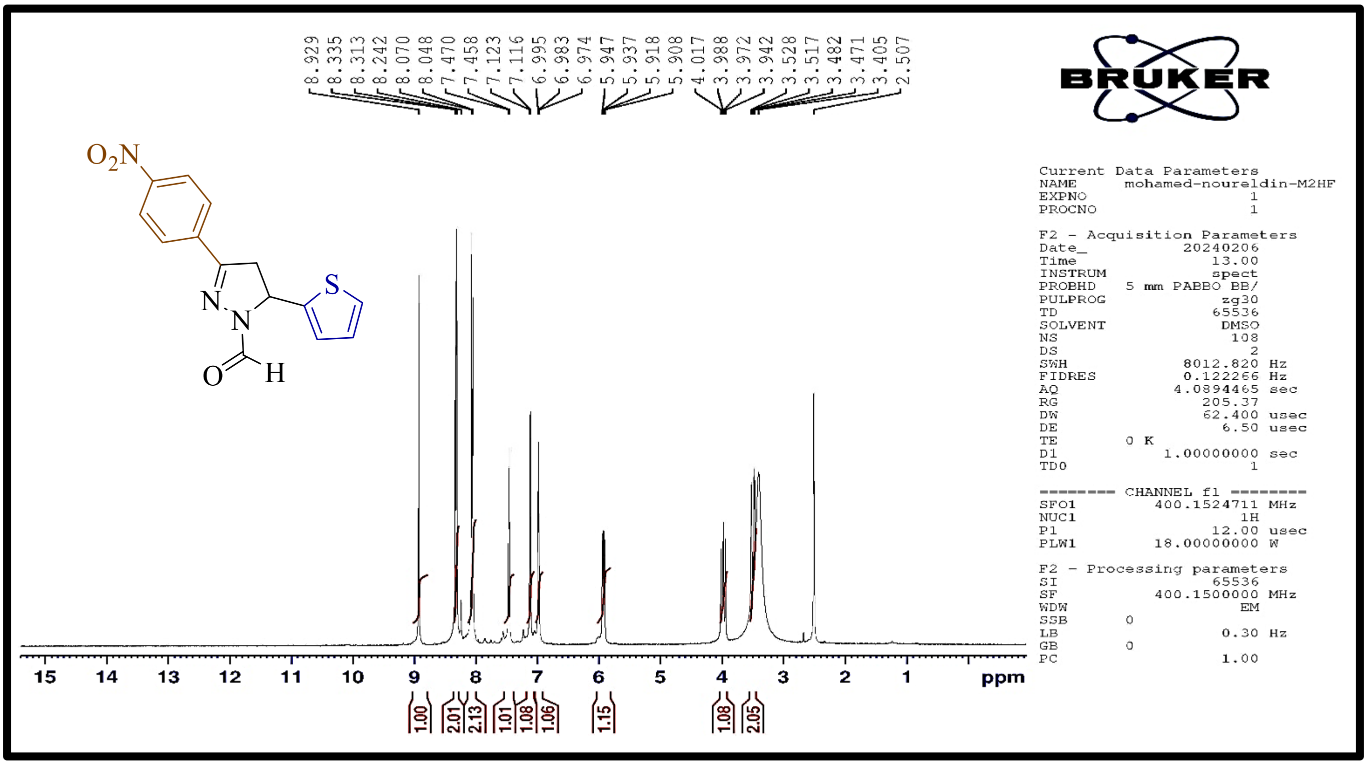

Figure S8. ^{1}H NMR (400 MHz, DMSO-d_6) spectrum of compound **4**

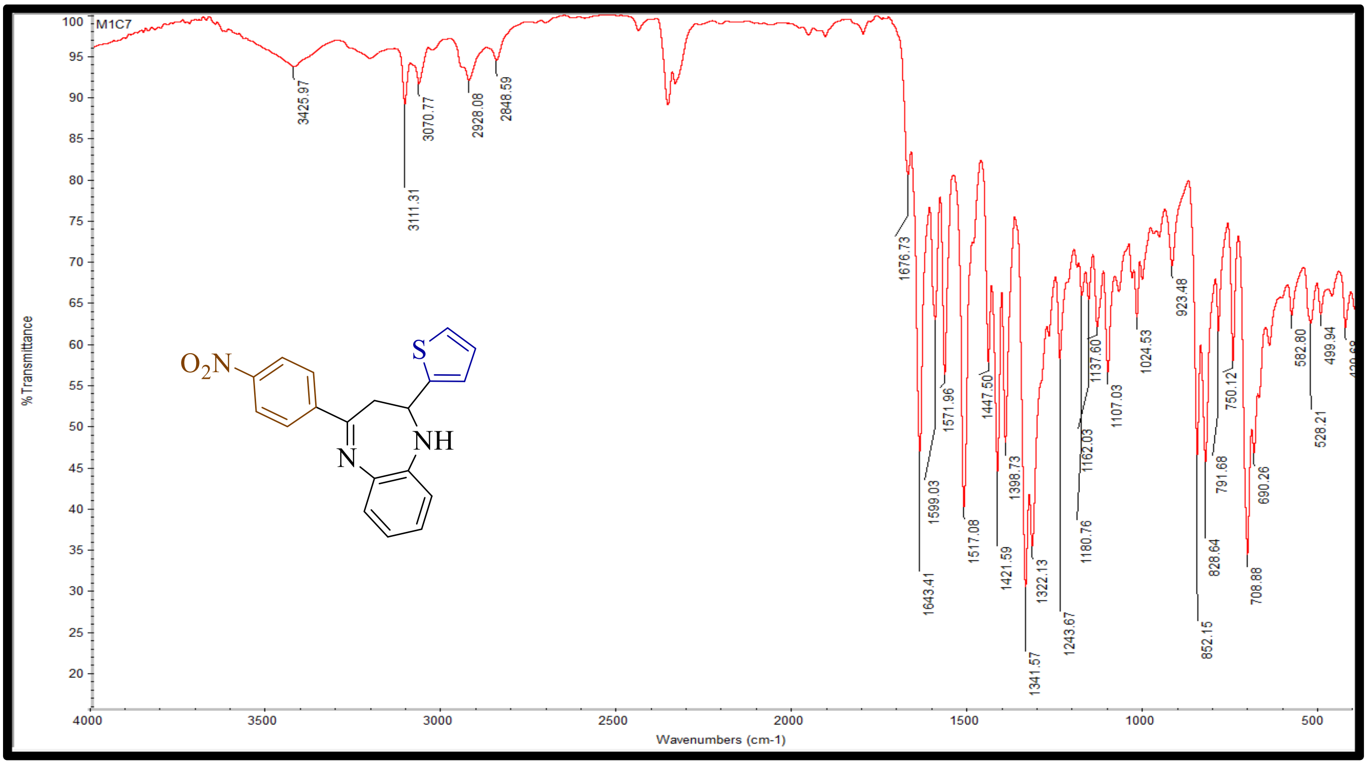

Figure S9. FTIR spectrum of compound **5**

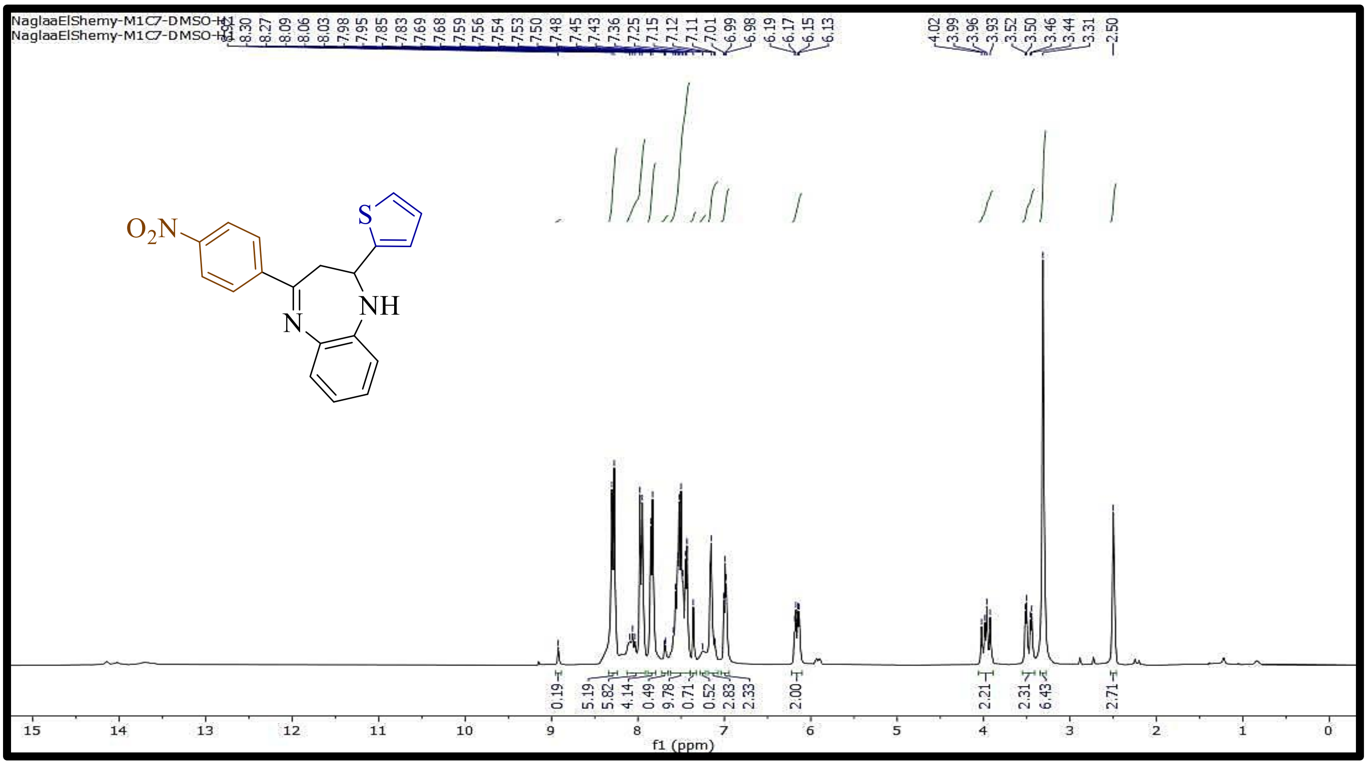

Figure S10. ^{1}H NMR (400 MHz, DMSO-d_6) spectrum of compound **5**

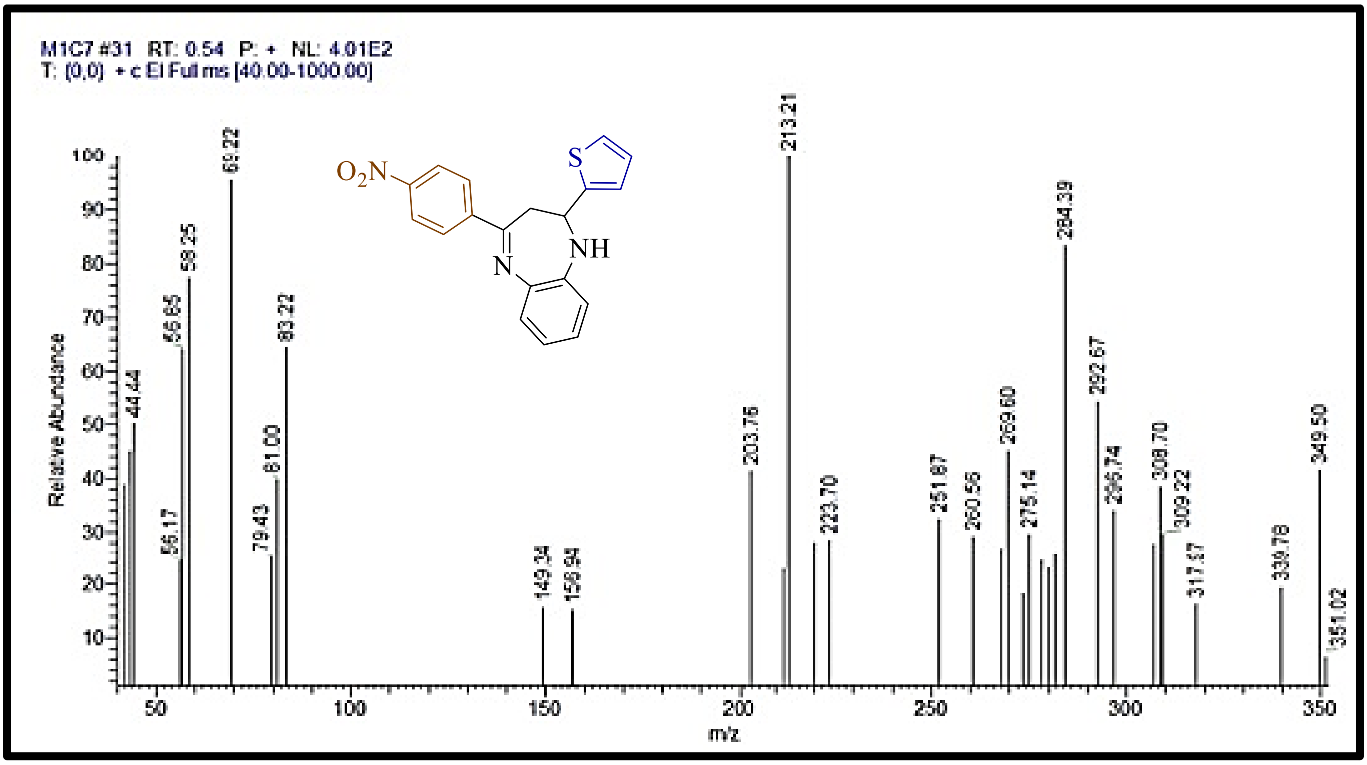

Figure S11. Mass spectrum of compound **5**

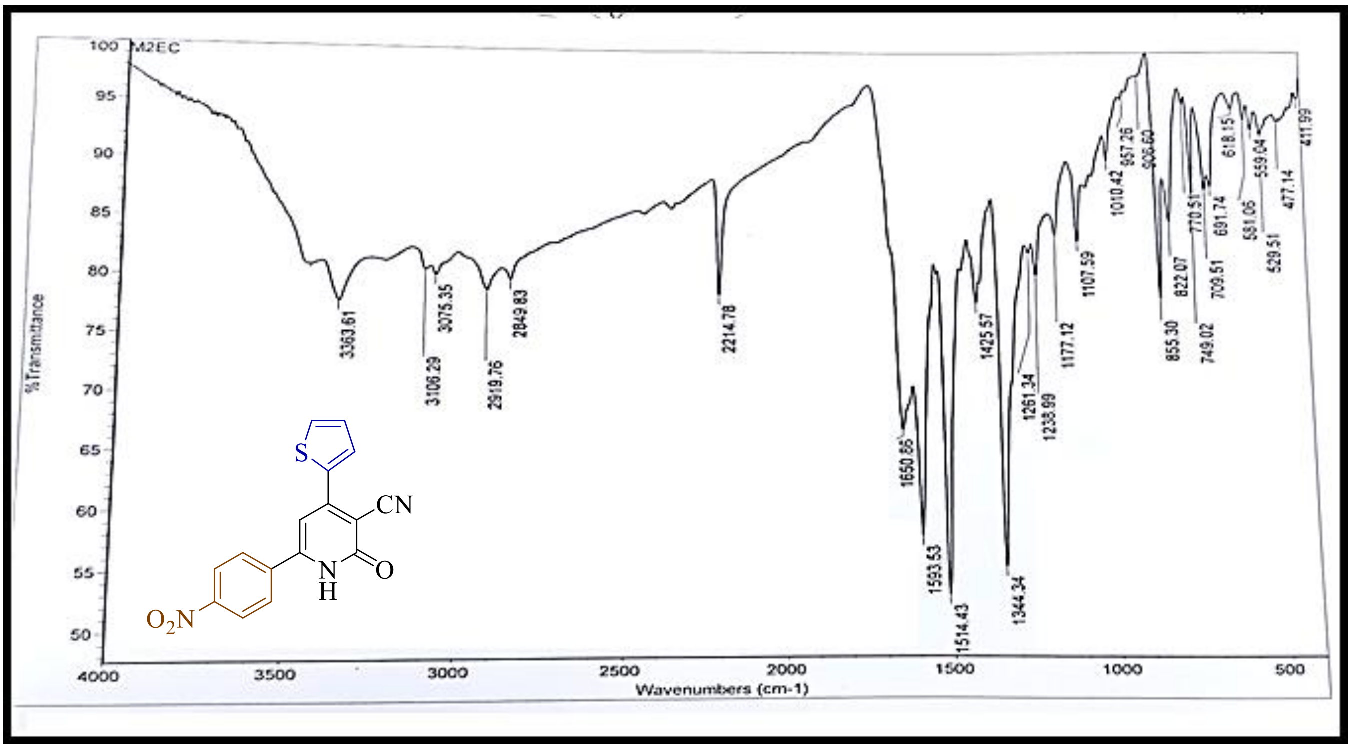

Figure S12. FTIR spectrum of compound **6**

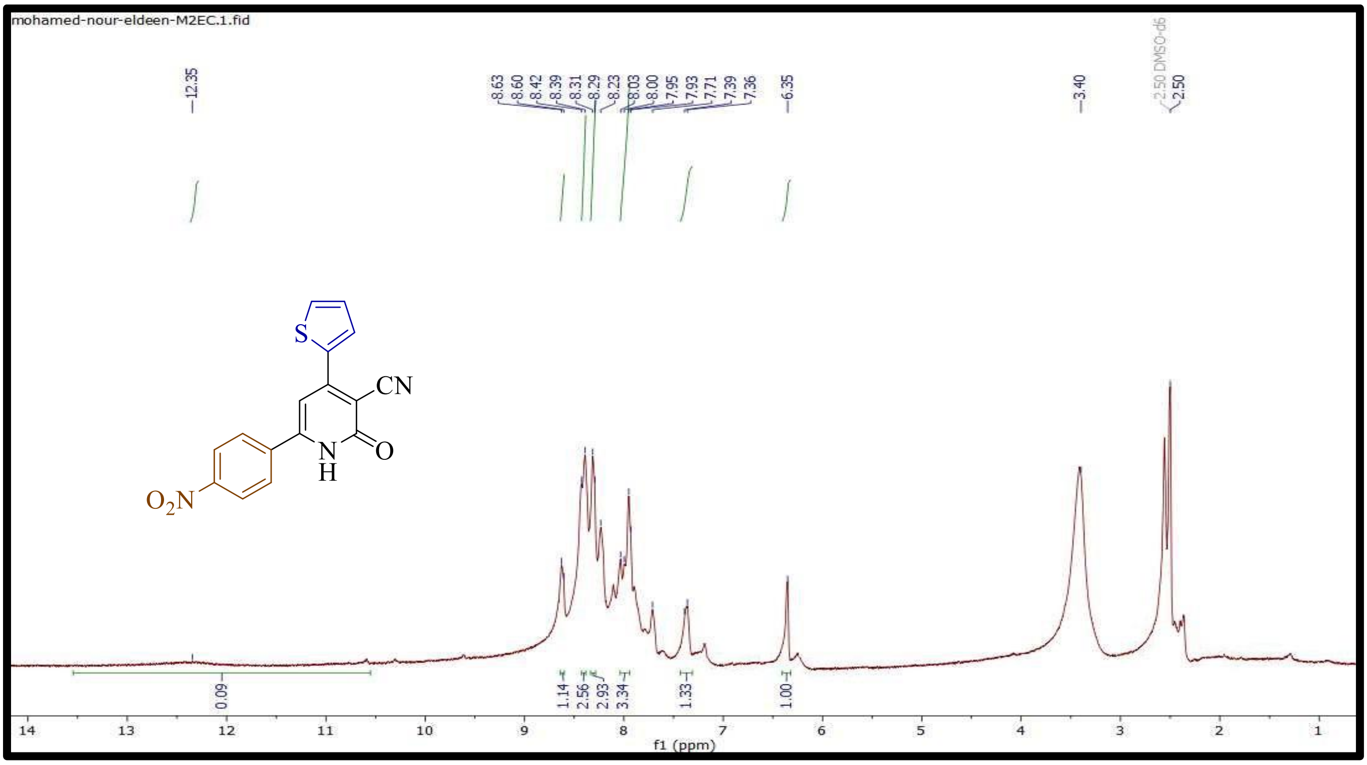

Figure S13. ^{1}H NMR (400 MHz, DMSO-d_6) spectrum of compound **6**

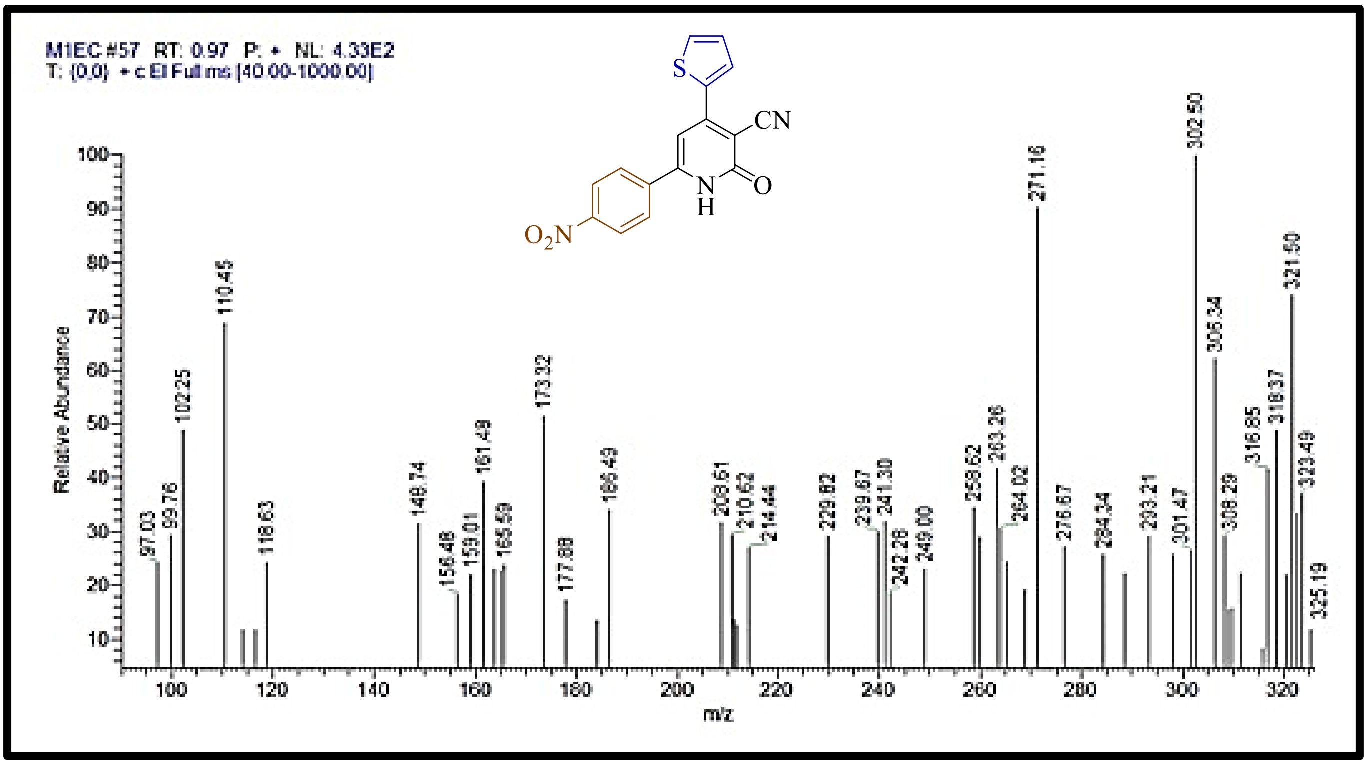

Figure S14. Mass spectrum of compound **6**

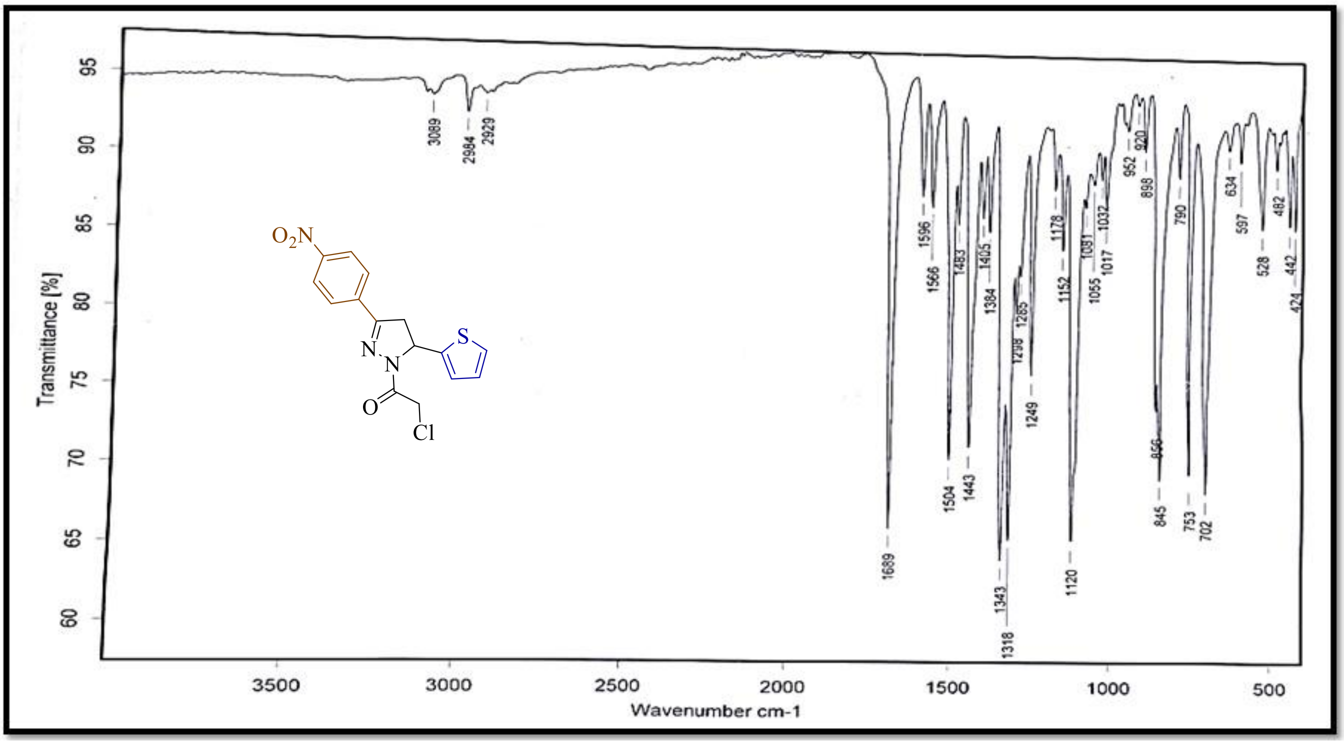

Figure S15. FTIR spectrum of compound **7**

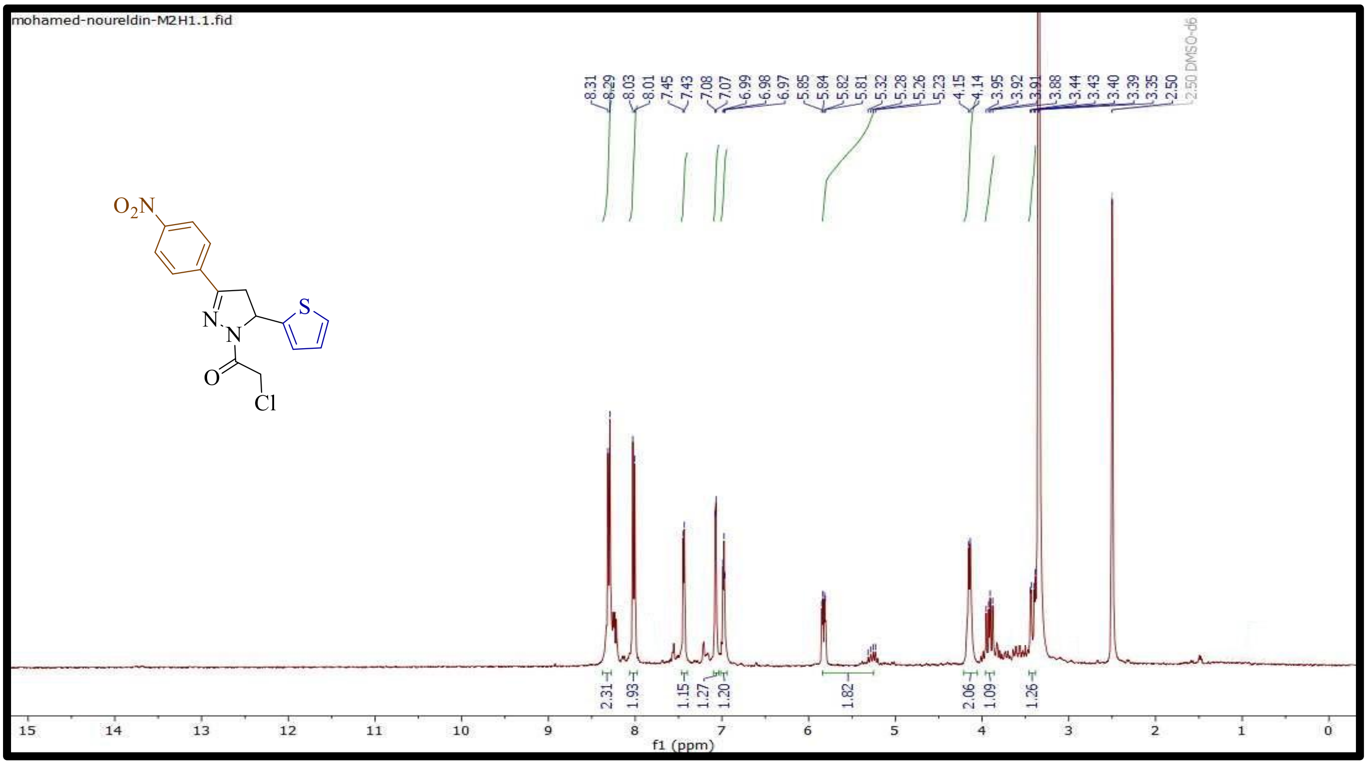

Figure S16. ^{1}H NMR (400 MHz, DMSO-d_6) spectrum of compound **7**

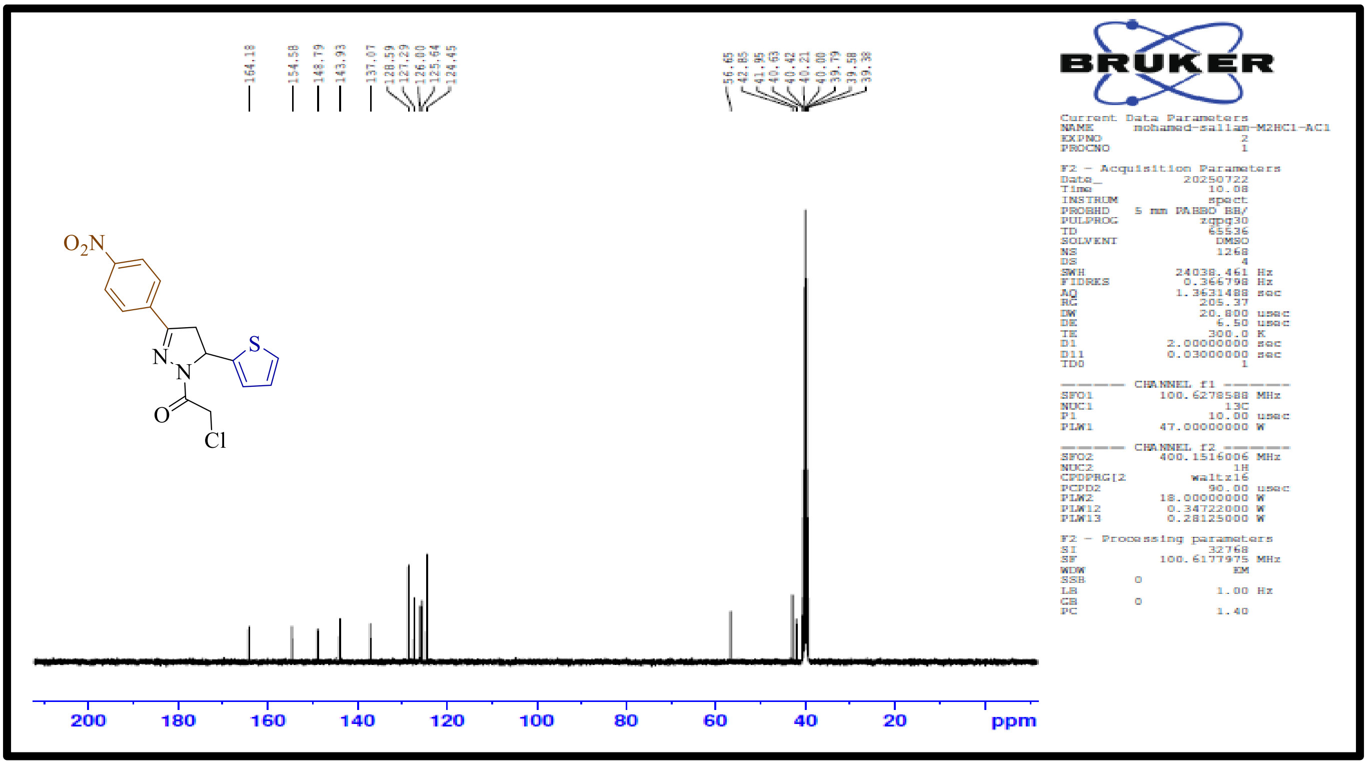

Figure S17. ^{13}C-NMR (100 MHz, DMSO-d_6) spectrum of compound **7**

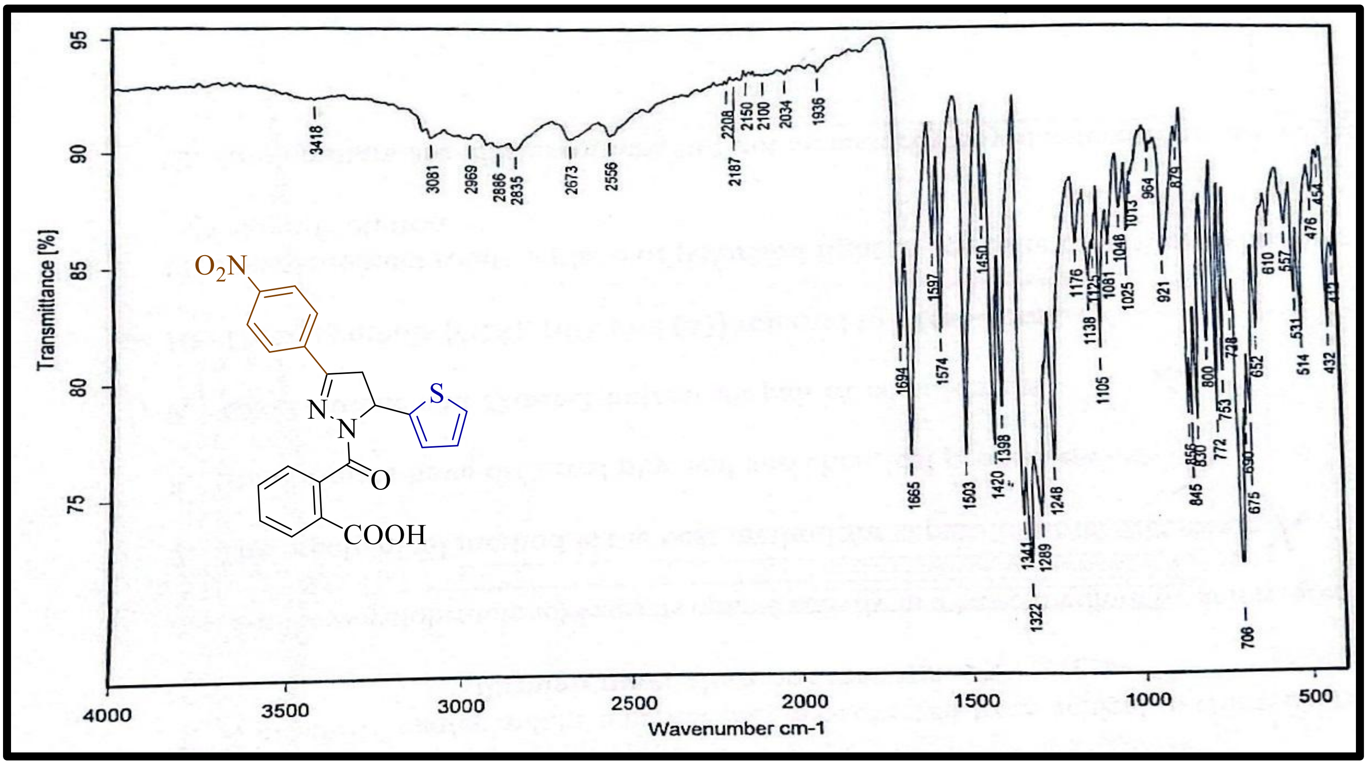

Figure S18. FTIR spectrum of compound **8**

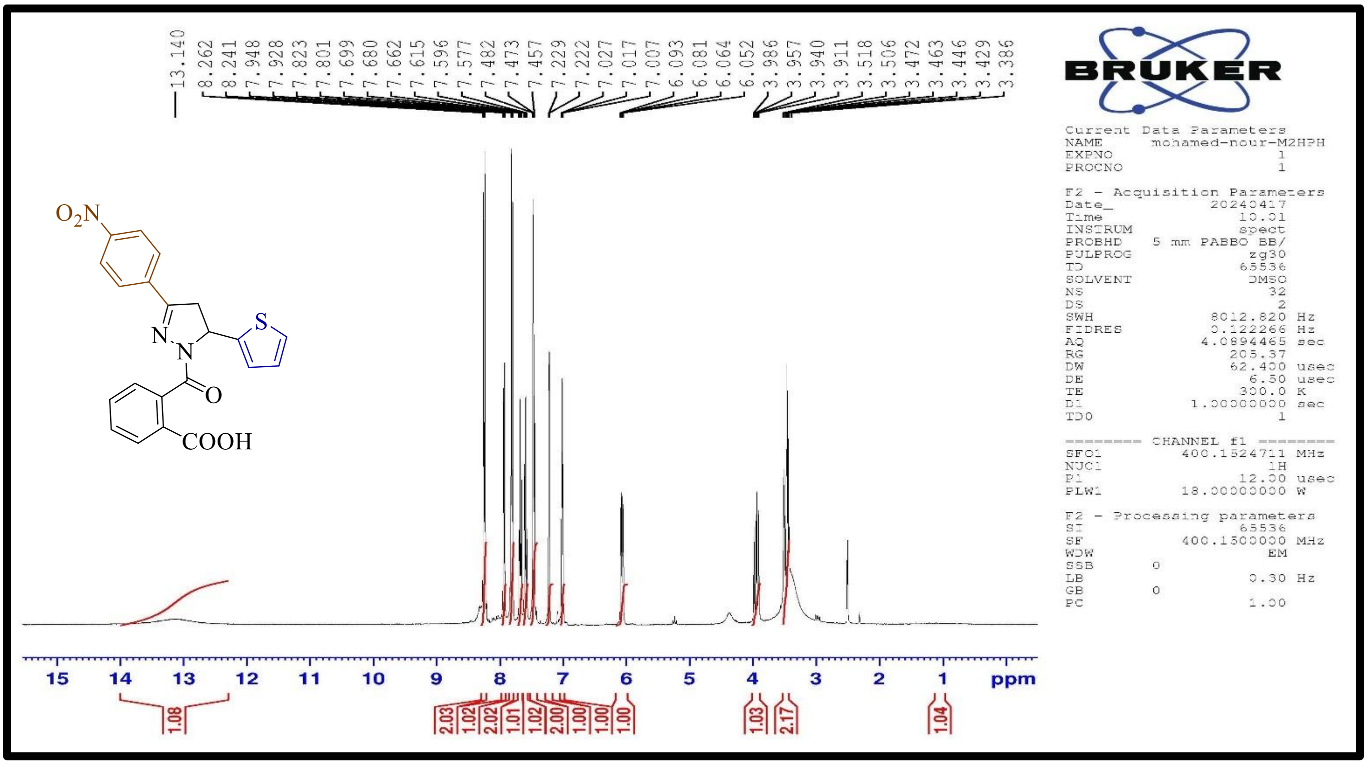

Figure S19. ^{1}H NMR (400 MHz, DMSO-d_6) spectrum of compound **8**

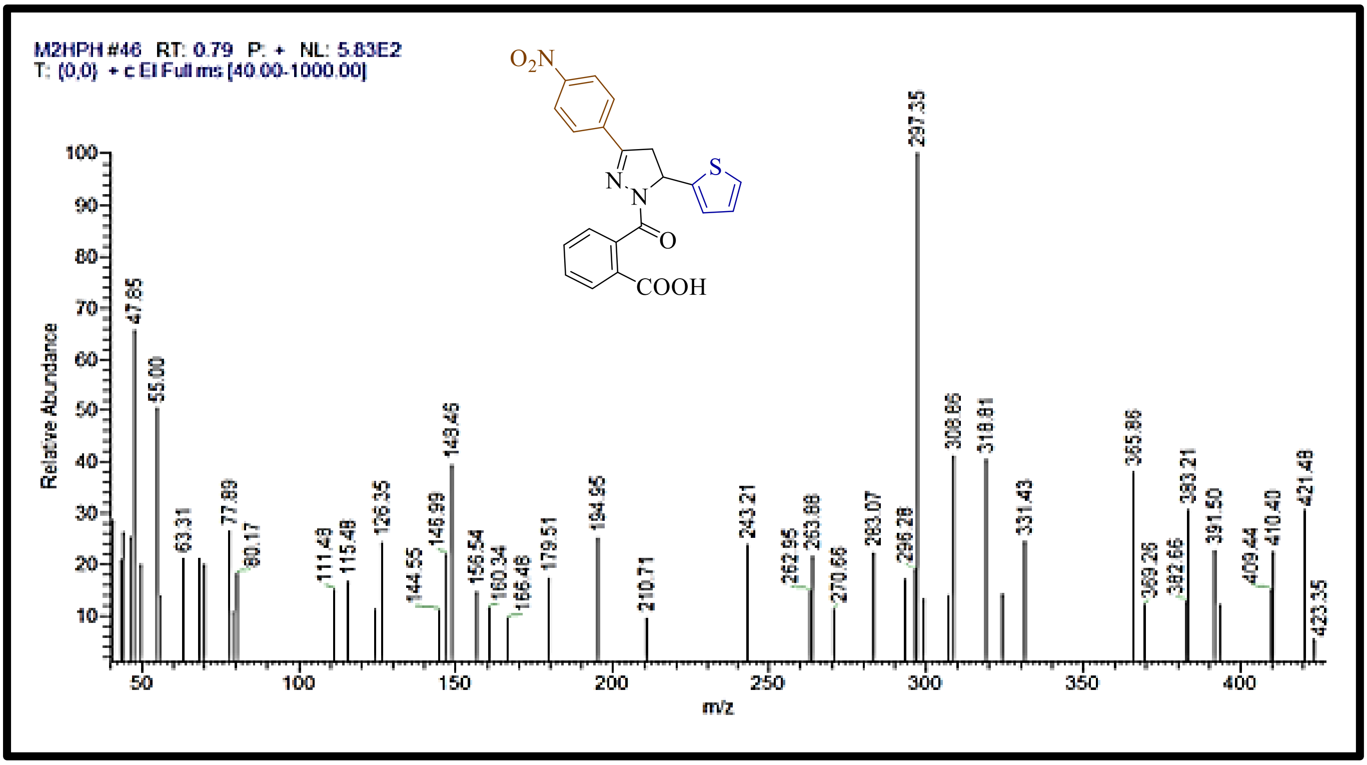

Figure S20. Mass spectrum of compound **8**

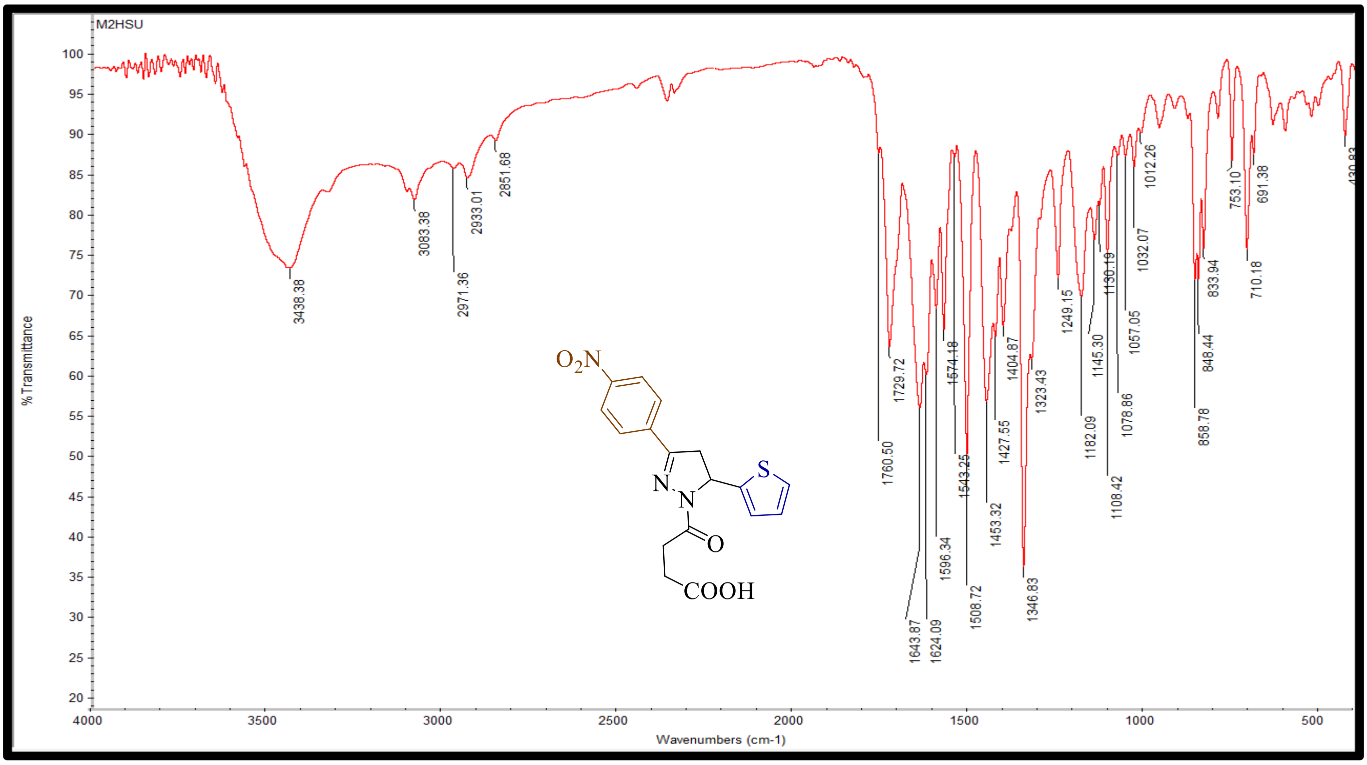

Figure S21. FTIR spectrum of compound **9**

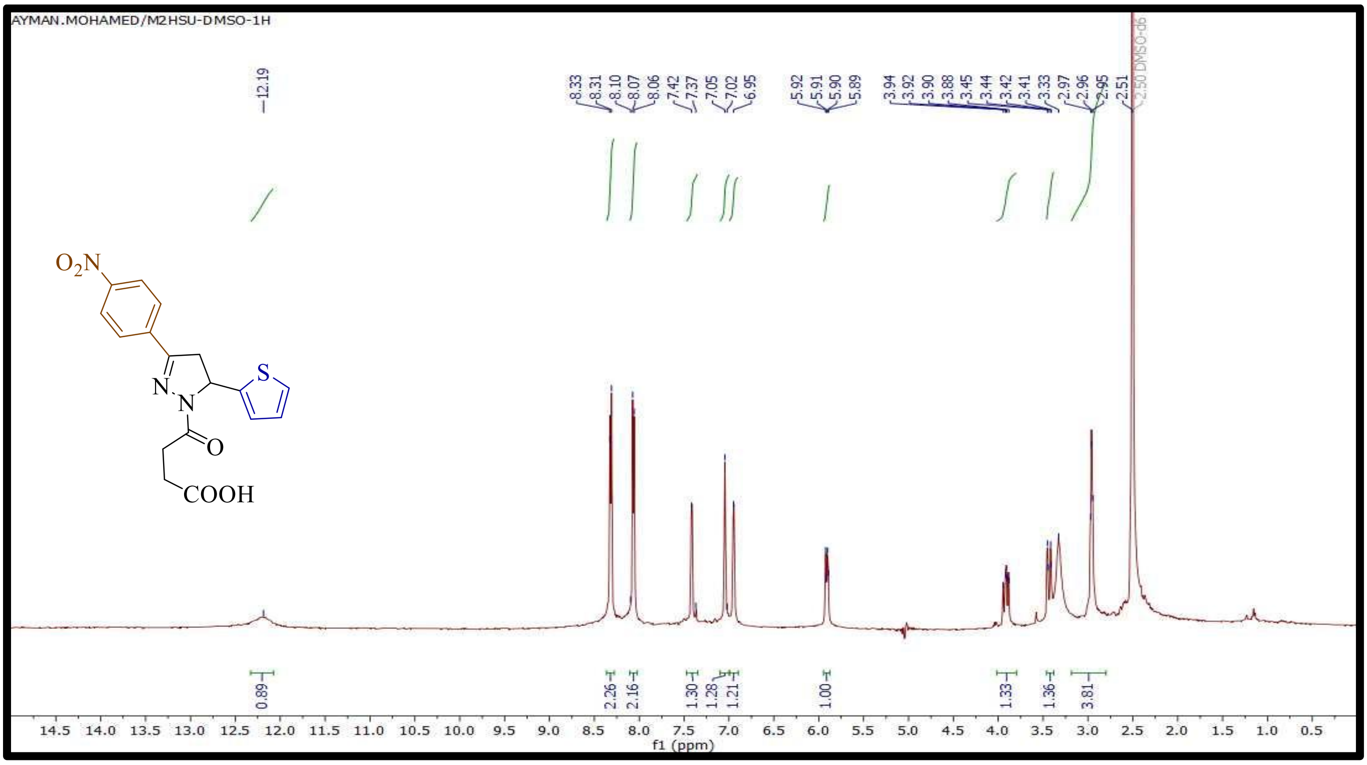

Figure S22. ^{1}H NMR (400 MHz, DMSO-d_6) spectrum of compound **9**

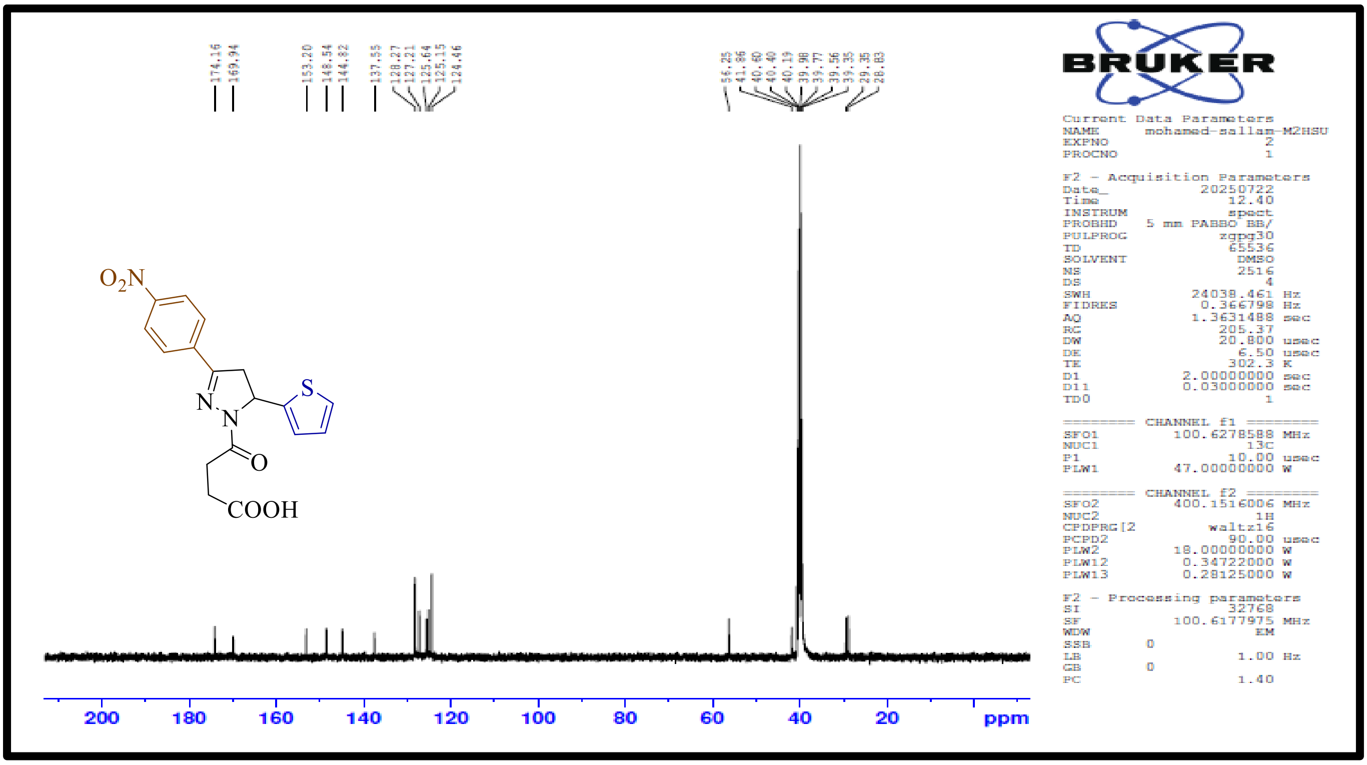

Figure S23. ^{13}C NMR (100 MHz, DMSO-d_6) spectrum of compound **9**

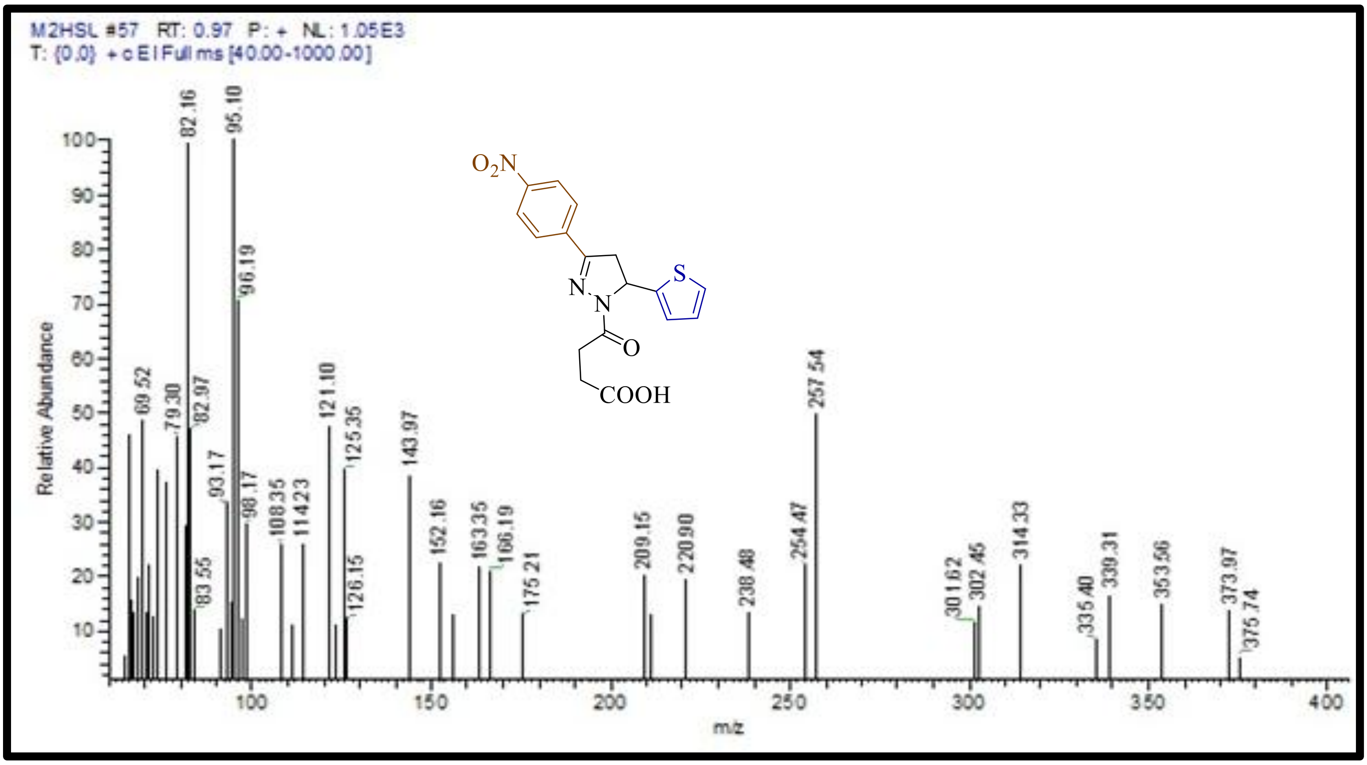

Figure S24. Mass spectrum of compound **9**

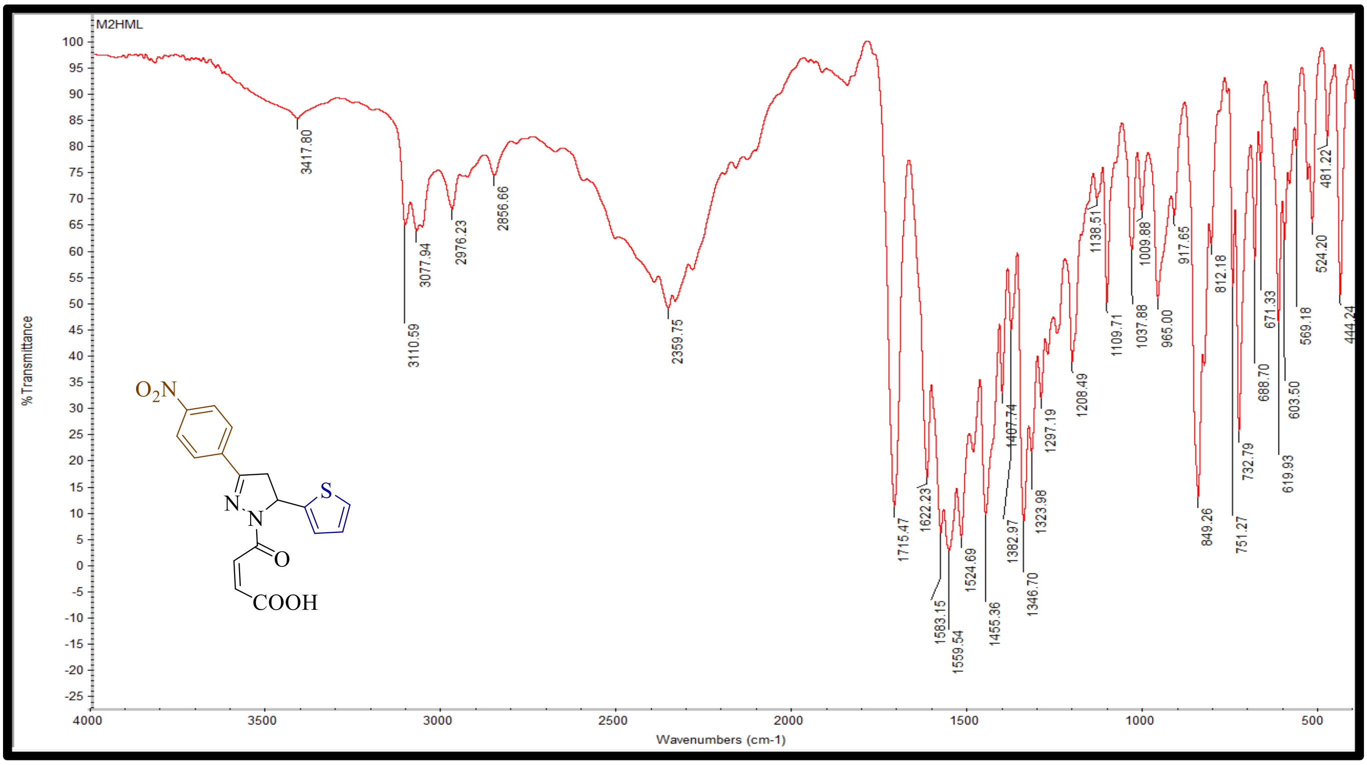

Figure S25. FTIR spectrum of compound **10**

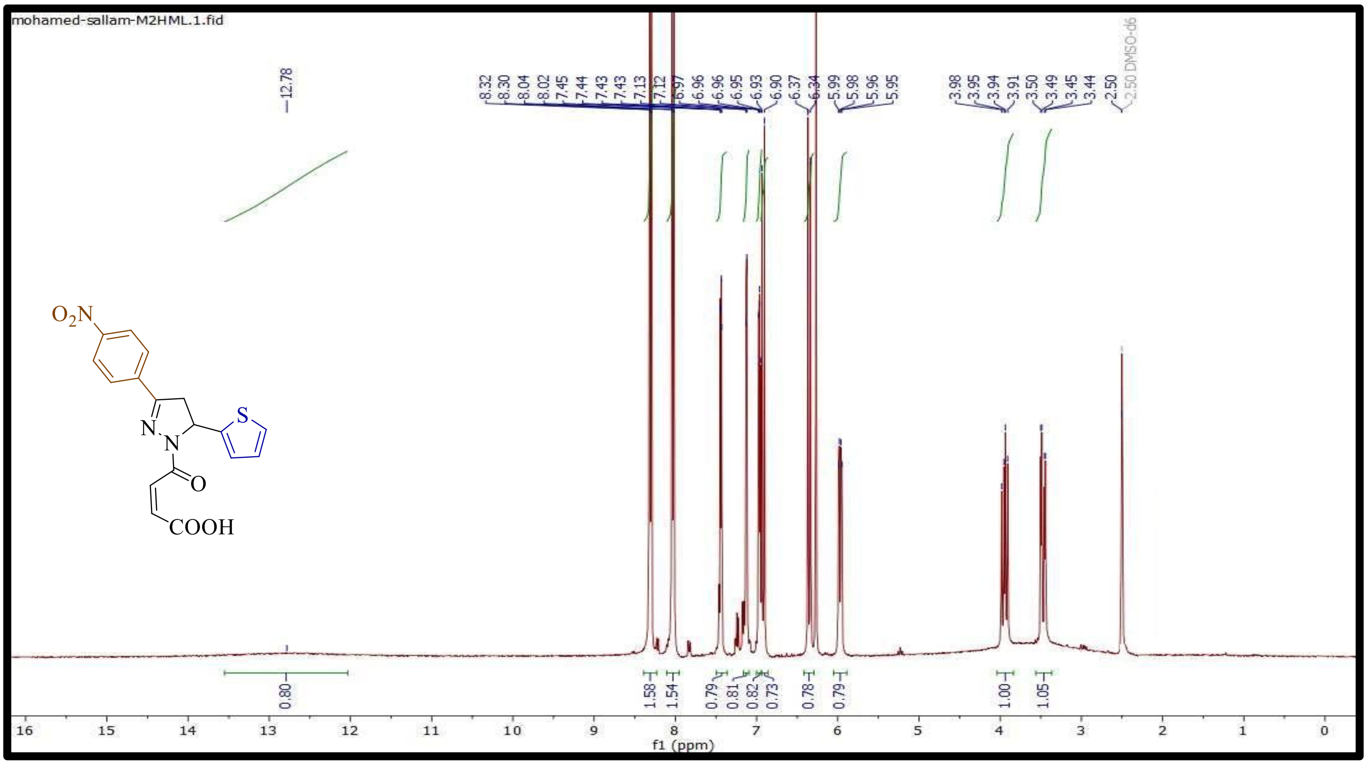

Figure S26. ^{1}H NMR (400 MHz, DMSO-d_6) spectrum of compound **10**

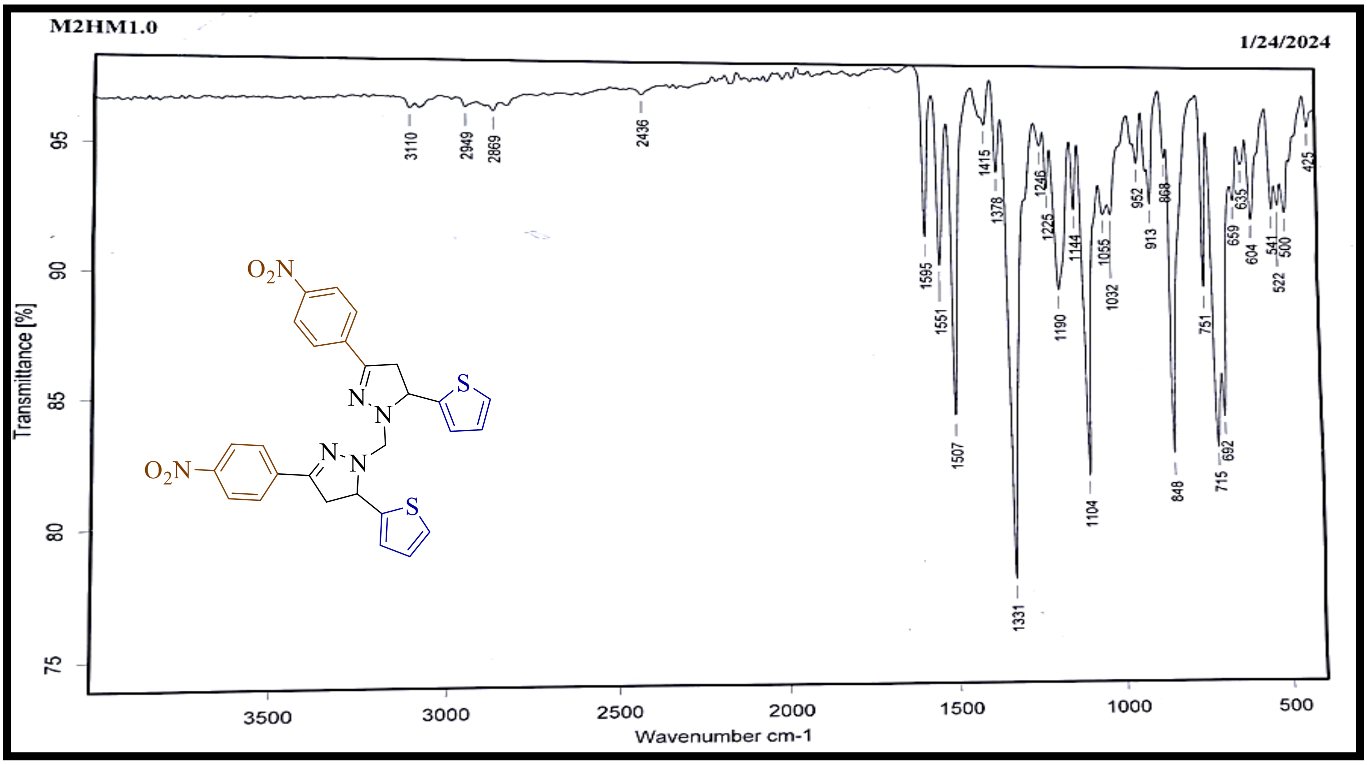

Figure S27. FTIR spectrum of compound **11**

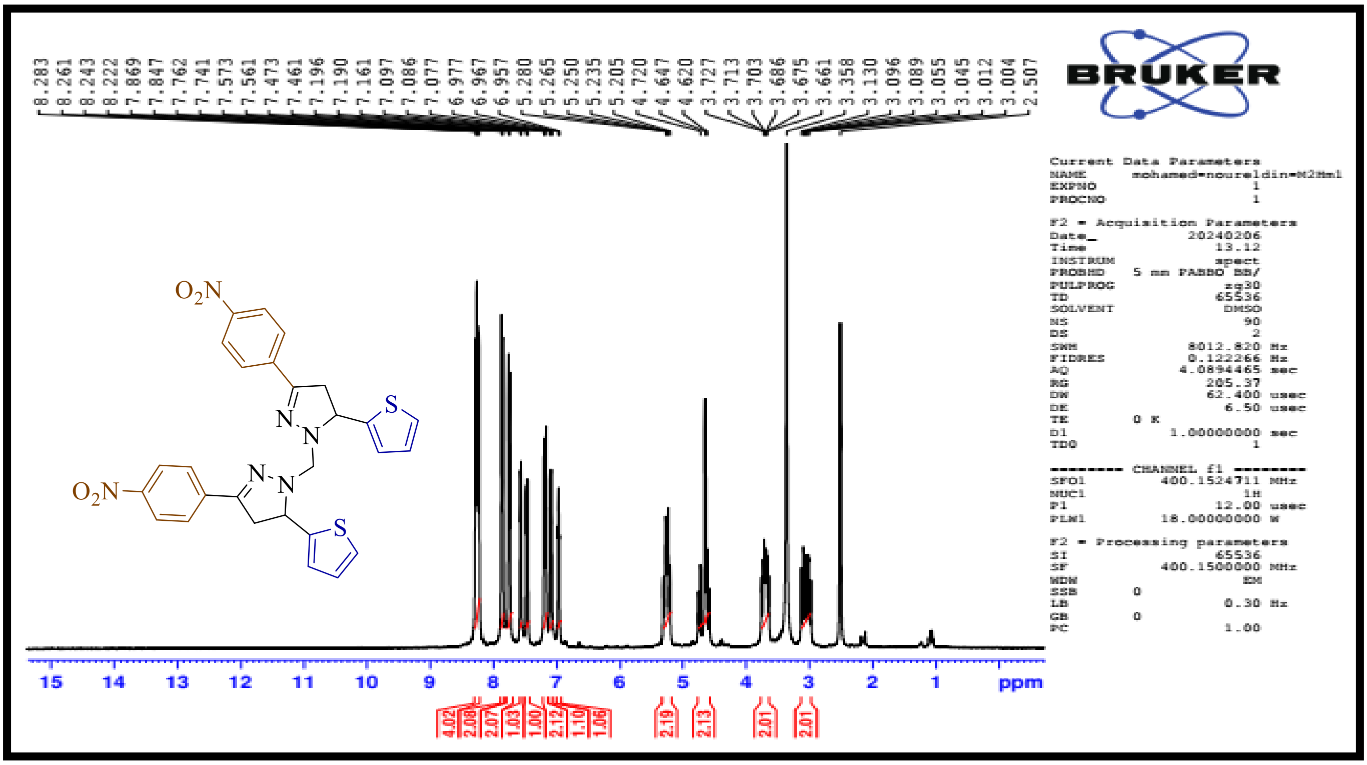

Figure S28. ^{1}H NMR (400 MHz, DMSO-d_6) spectrum of compound **11**

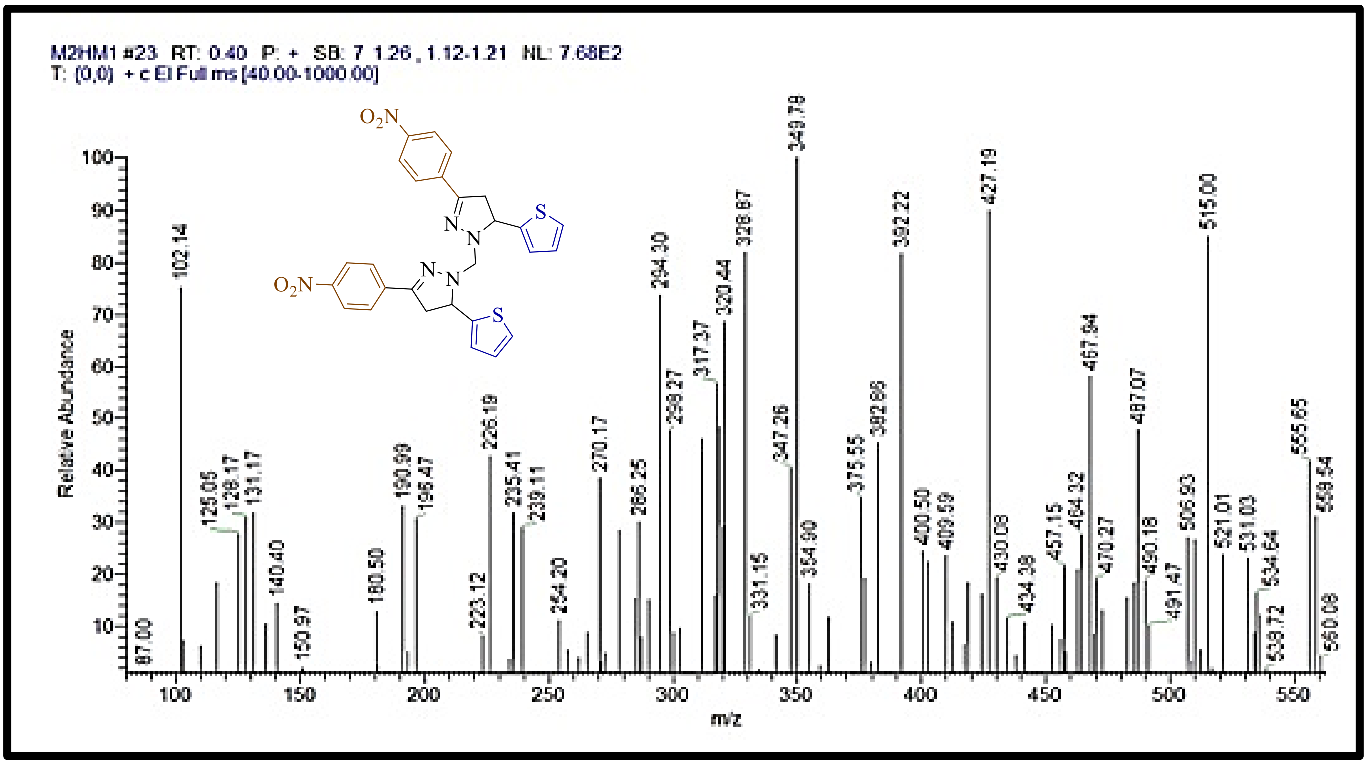

Figure S29. Mass spectrum of compound **11**

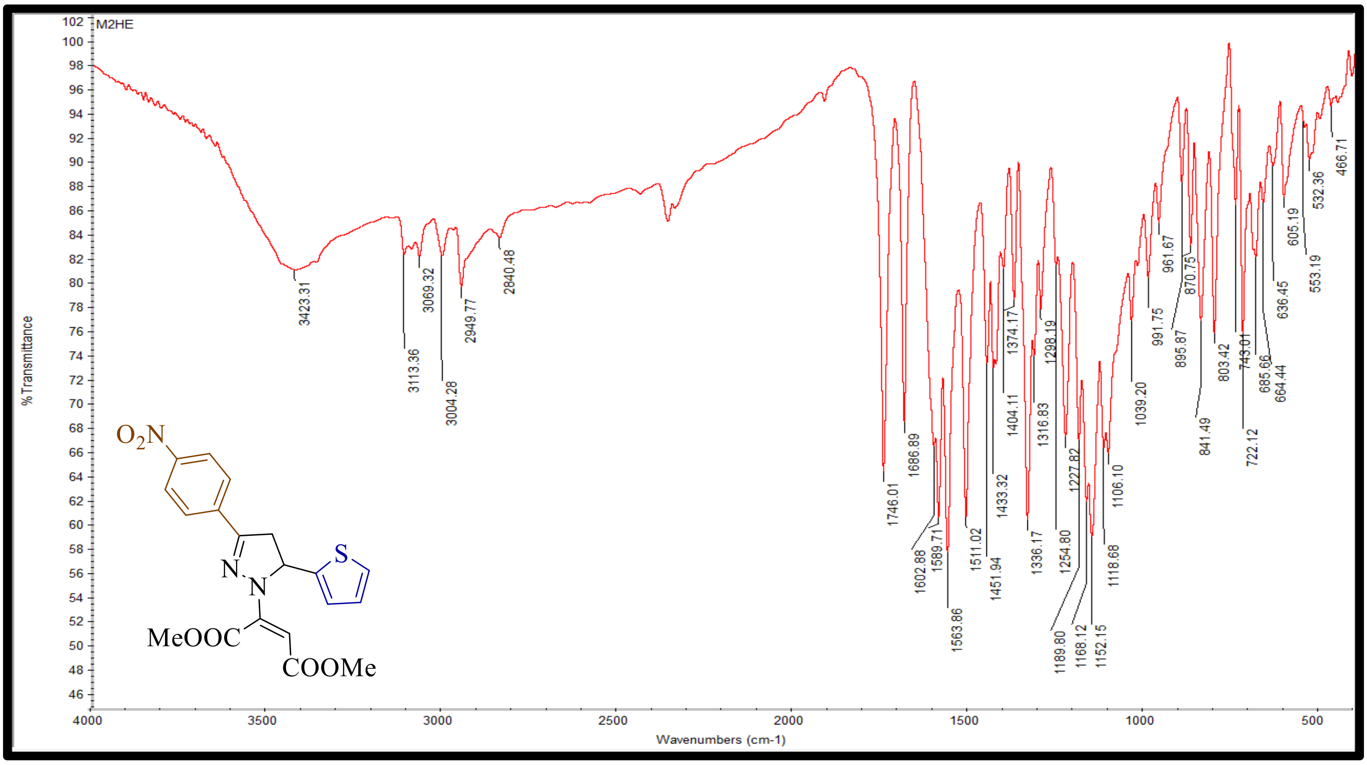

Figure S30. FTIR spectrum of compound **12**

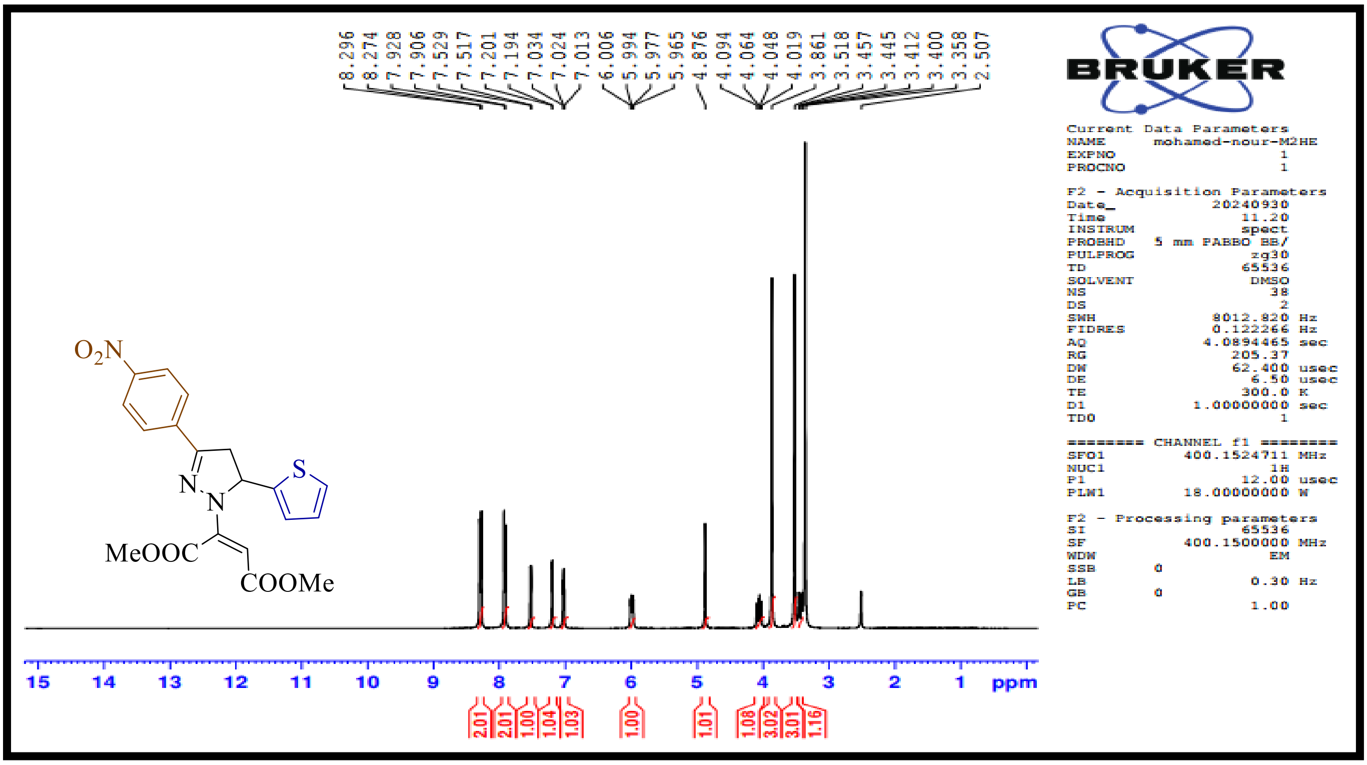

Figure S31. ^{1}H NMR (400 MHz, DMSO-d_6) spectrum of compound **12**

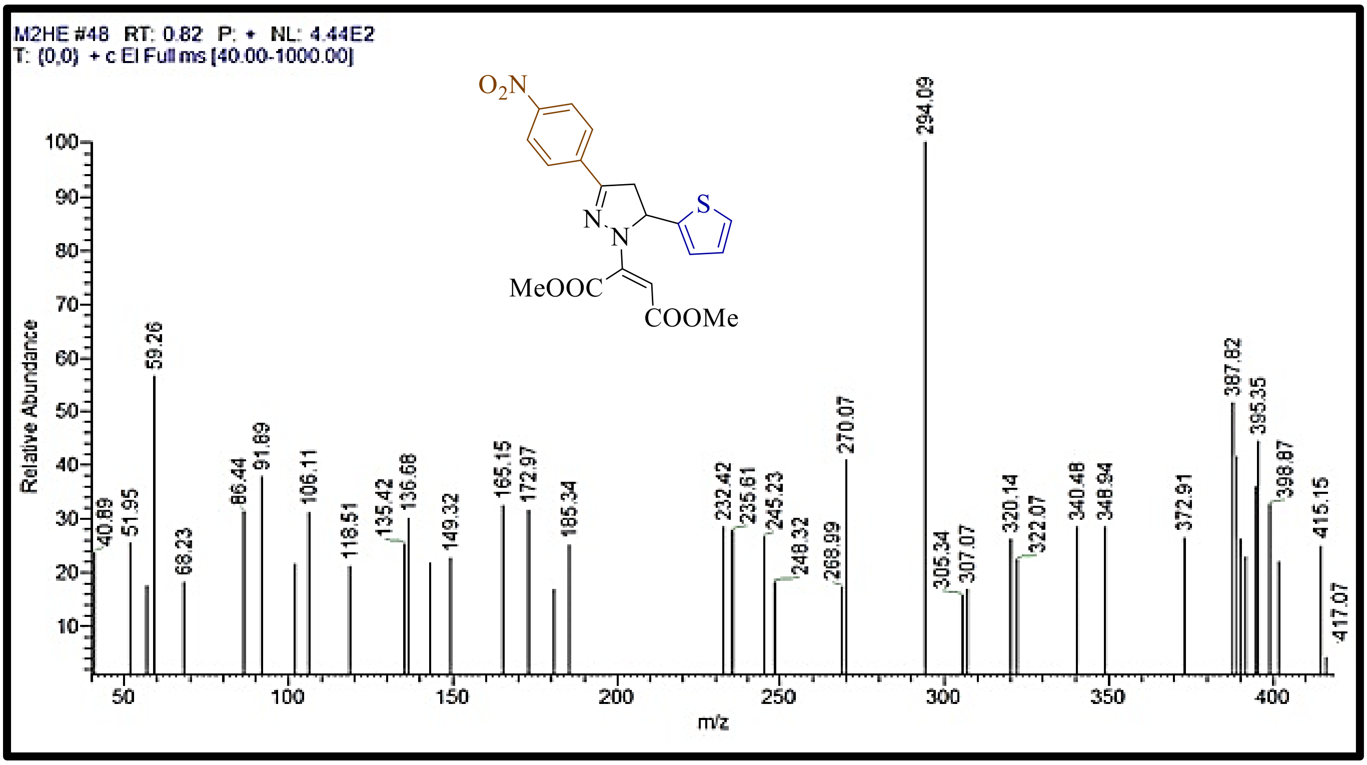

Figure S32. Mass spectrum of compound **12**

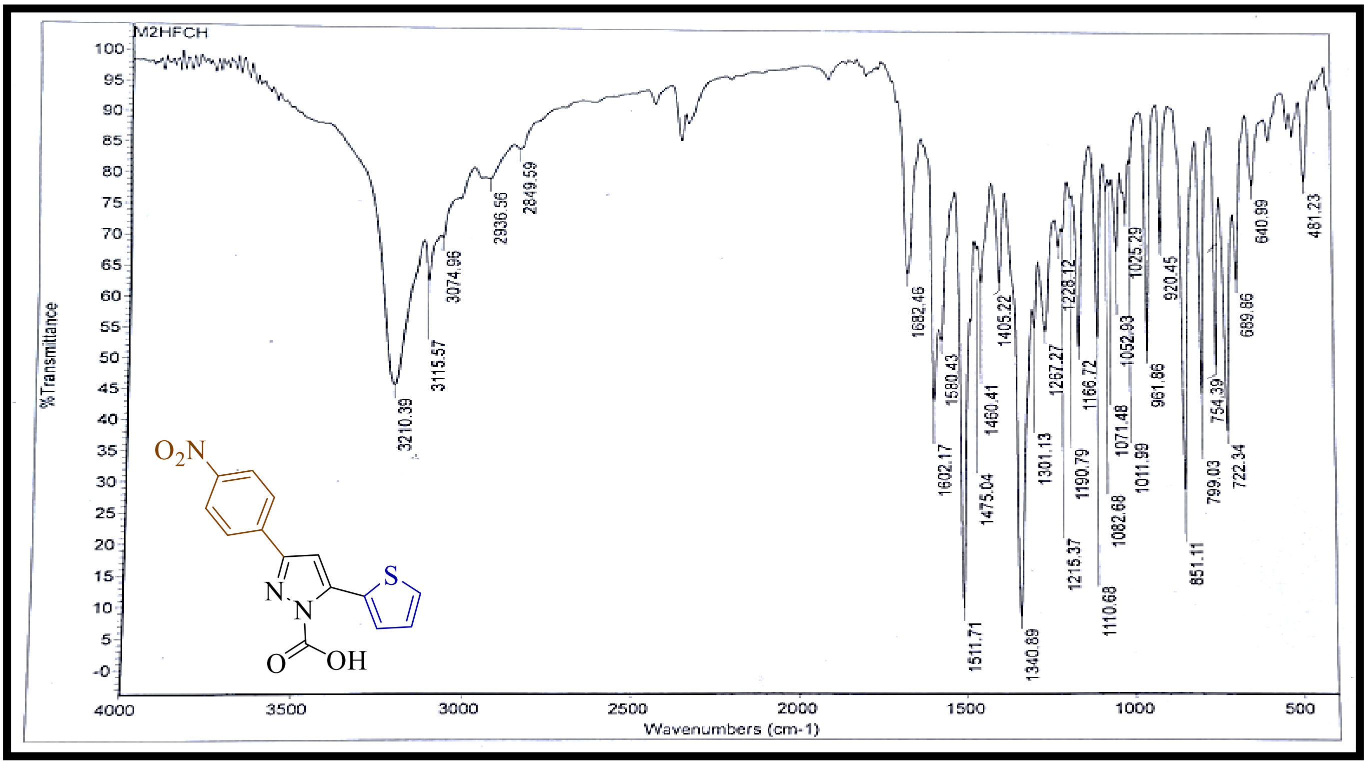

Figure S33. FTIR spectrum of compound **13**

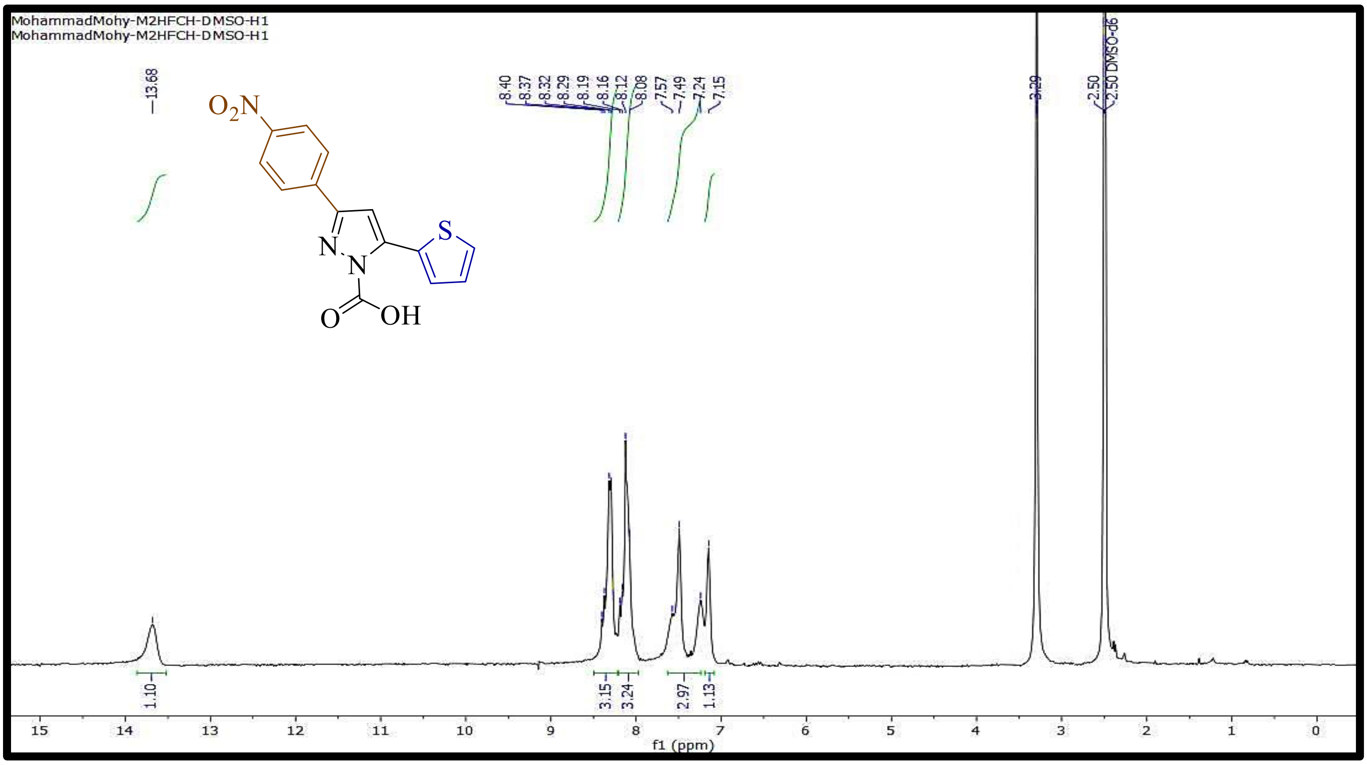

Figure S34. ^{1}H NMR (400 MHz, DMSO-d_6) spectrum of compound **13**

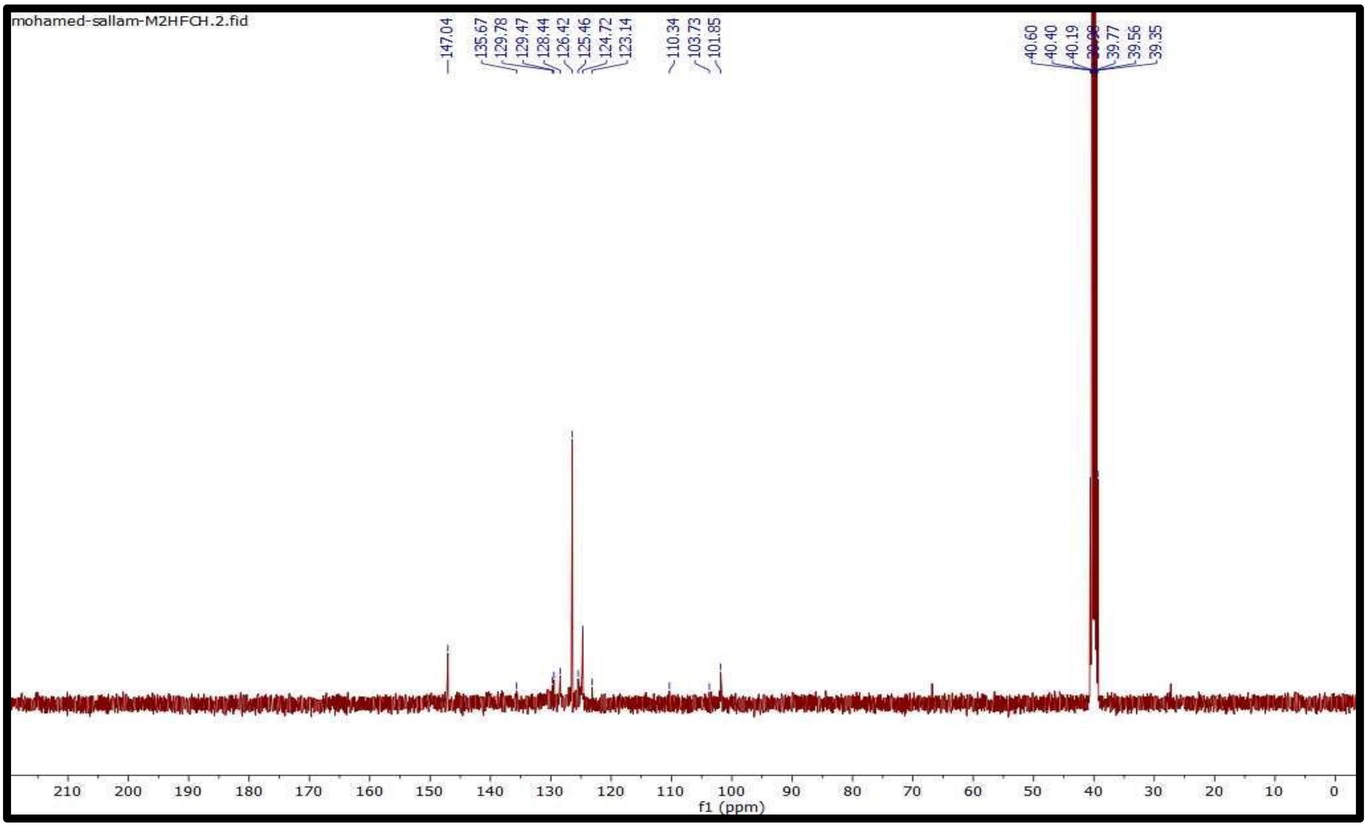

Figure S35. ^{13}C NMR (100 MHz, DMSO-d_6) spectrum of compound **13**

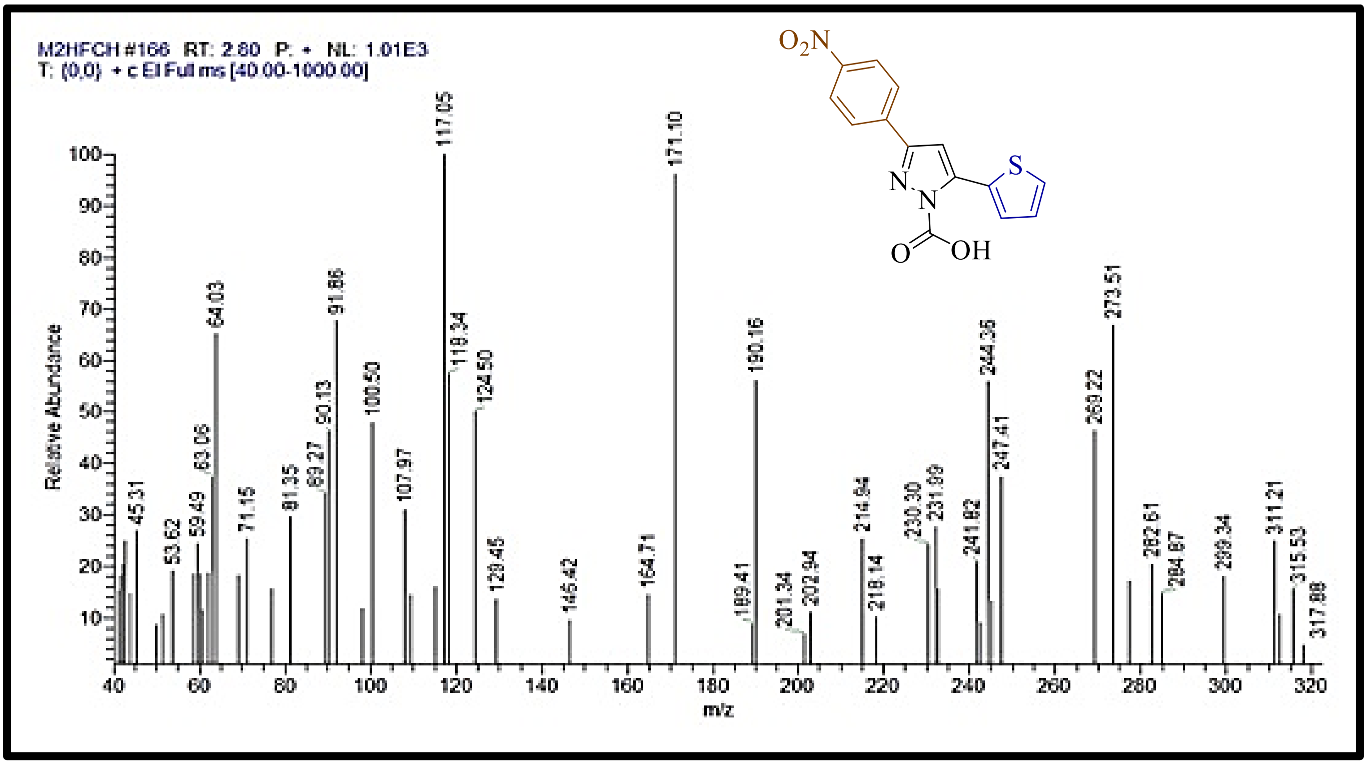

Figure S36. Mass spectrum of compound **13**

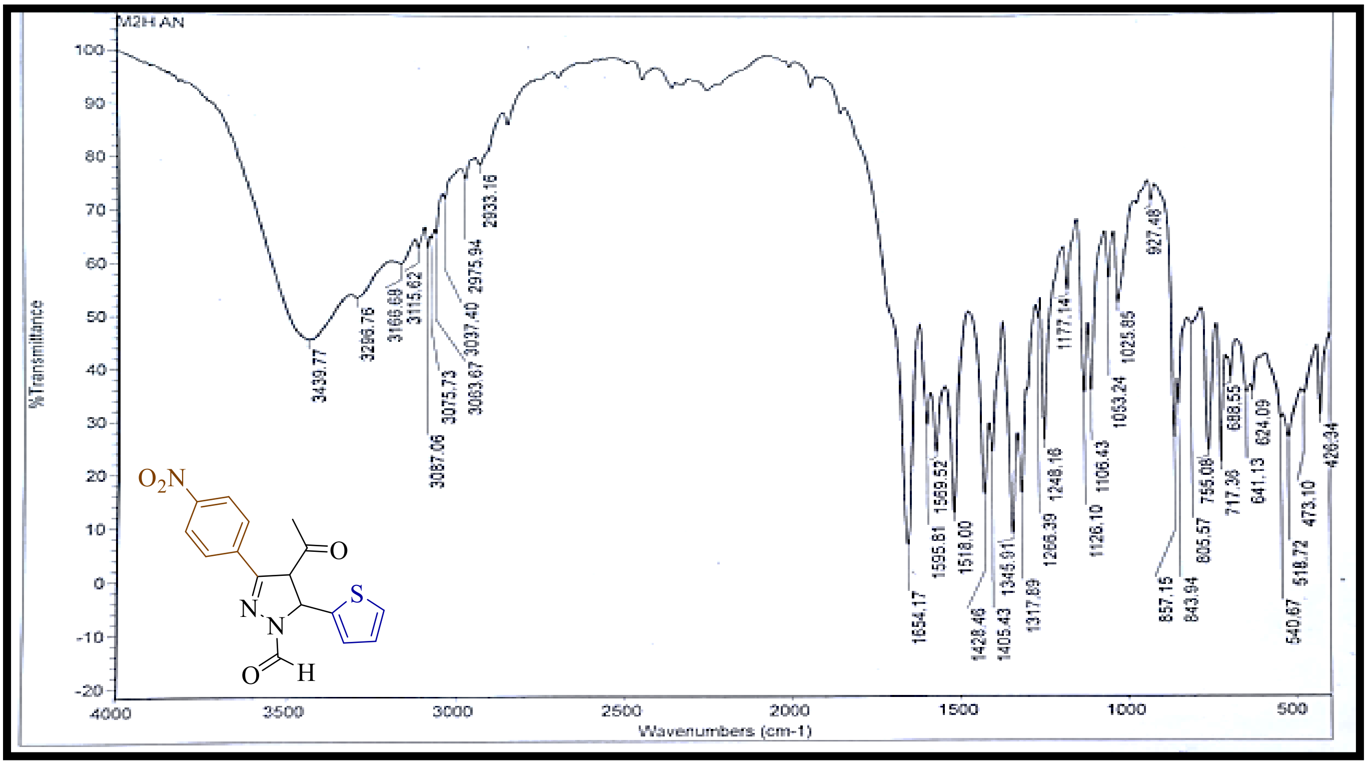

Figure S37. FTIR spectrum of compound **14**

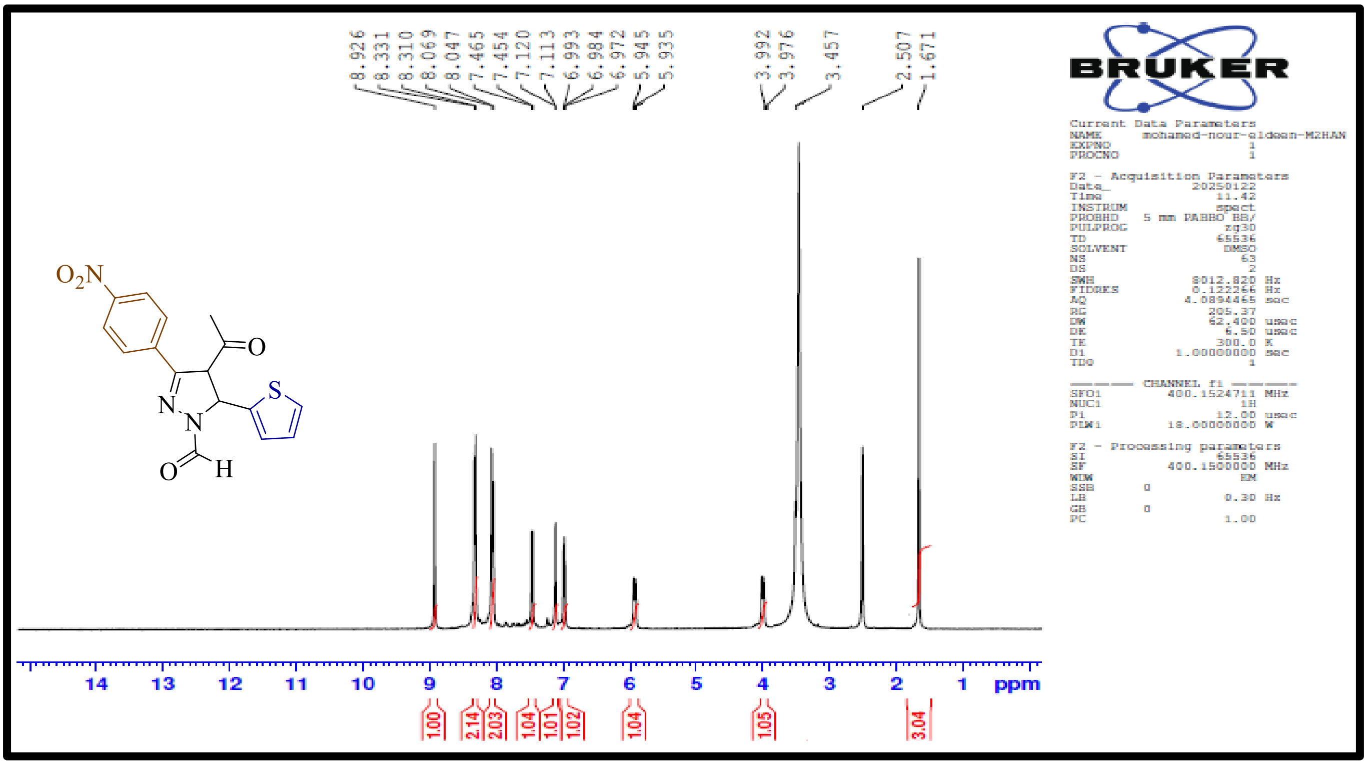

Figure S38. ^{1}H NMR (400 MHz, DMSO-d_6) spectrum of compound **14**

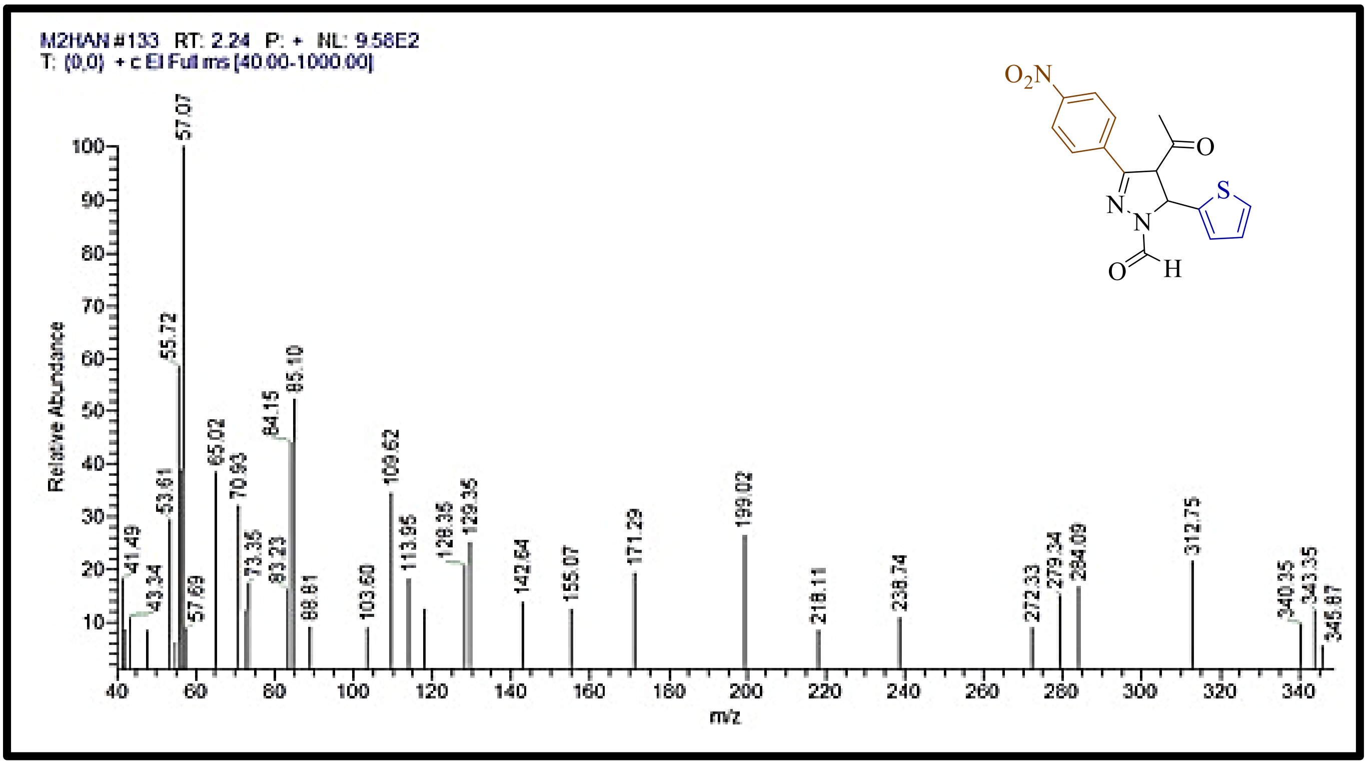

Figure S39. Mass spectrum of compound **14**

HPLC of the most potent candidates 2, 8, and 14

The chromatographic analysis was conducted using a Shimadzu HPLC system with column Kromasil C 18 micron of 150x4.6 mm (I.D.); flow phase comprising ACN-H_2O-MEOH (30/65/5) (v/v/v) as the mobile phase, and detection was operated by UV absorption at a wavelength of 250 nm.

SHIMADZU
LabSolutions

Liquid Chromatograph Report

Sample Name	SAMPLE 2	Injection No	
Operator		Analysis Time	
Sender		Send Time	
Sample Type	Unknown	Method Name	Use the method of last sample Fixed

Host Model — Liquid Chromatograph

Injection Method	Manual	Current Detector	UV-Detector	Flow	1.00 ml/min
Inject Volume	25 ul	Wavelength	250 nm	Column Info	C_{18}

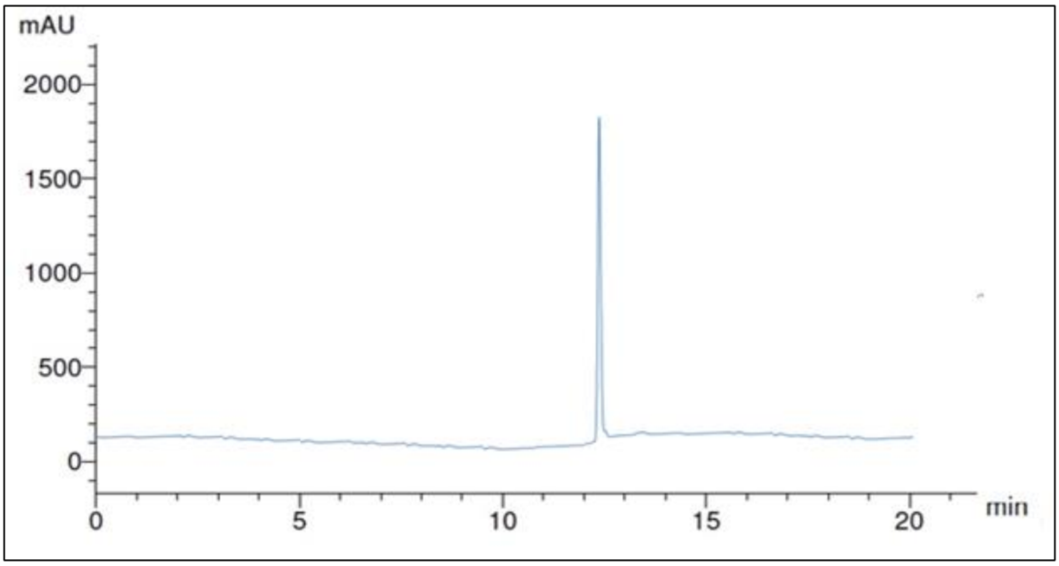

Integration Results Table

No	RT time	Width (min)	Height (mAU)	Area (mAU.s)	Area %
1	12.3	0.540	1900.05	972.25	99.14

Figure S40. HPLC of compound **2**

SHIMADZU
LabSolutions

Liquid Chromatograph Report

Sample Name	SAMPLE 8	Injection No	
Operator		Analysis Time	
Sender		Send Time	
Sample Type	Unknown	Method Name	Use the method of last sample<br>Fixed

Host Model	Liquid Chromatograph

Injection Method	Manual	Current Detector	UV-Detector	Flow	1.00 ml/min
Inject Volume	25 ul	Wavelength	250 nm	Column Info	C_{18}

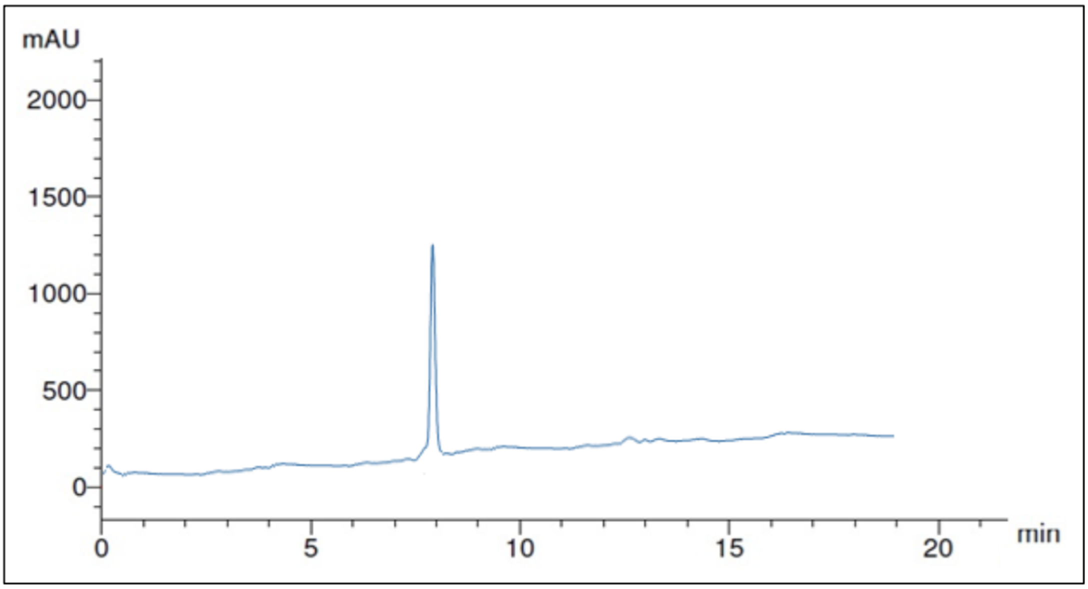

Integration Results Table

No	RT time	Width (min)	Height (mAU)	Area (mAU.s)	Area %
1	8.0	0.398	1221.01	905.12	98.16

Figure S41. HPLC of compound **8**

SHIMADZU LabSolutions

Liquid Chromatograph Report

Sample Name	SAMPLE 14	Injection No	
Operator		Analysis Time	
Sender		Send Time	
Sample Type	Unknown	Method Name	Use the method of last sample<br>Fixed

Host Model — Liquid Chromatograph

Injection Method	Manual	Current Detector	UV-Detector	Flow	1.00 ml/min
Inject Volume	25 ul	Wavelength	250 nm	Column Info	C_{18}

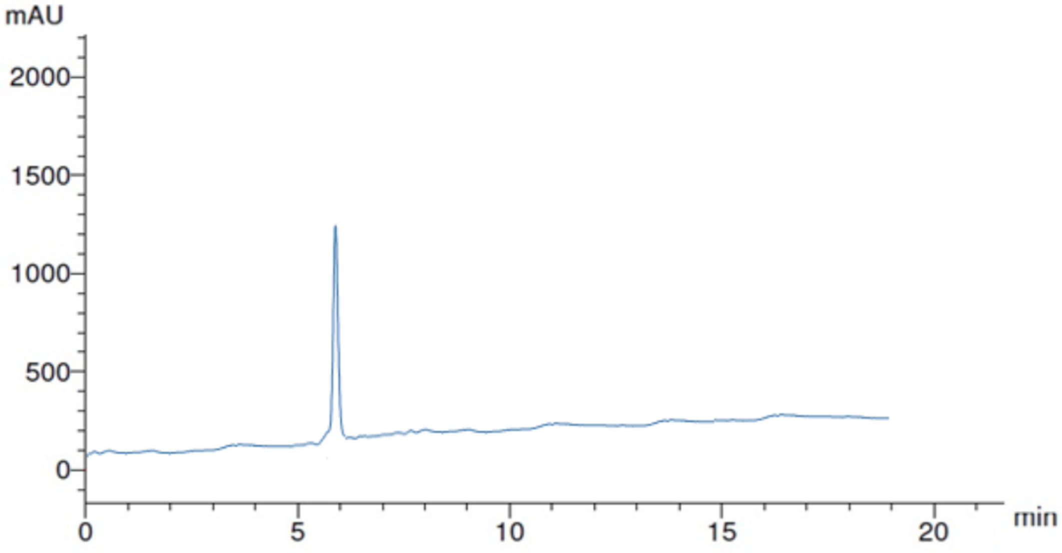

Integration Results Table

No	RT time	Width (min)	Height (mAU)	Area (mAU.s)	Area %
1	6.0	0.475	1289.10	944.14	99.91

Figure S42. HPLC of compound **14**

Supplementary Tables

Table S1. *In vitro* anticancer assay (IC_{50}, µM).

Compound no.	*In vitro* Cytotoxicity (IC_{50}, µM)	
	MCF-7	HepG2
**1**	>100	90.48 ± 4.5
**2**	**6.57 ± 0.4**	**8.86 ± 0.6**
**3**	43.60 ± 2.6	53.18 ± 3.0
**4**	92.12 ± 4.7	84.04 ± 4.2
**5**	75.54 ± 3.8	62.63 ± 3.5
**6**	23.29 ± 1.6	28.87 ± 1.9
**7**	75.01 ± 2.3	78.08 ± 3.9
**8**	**8.08 ± 0.6**	**13.51 ± 1.1**
**9**	**17.80 ± 1.3**	**22.62 ± 1.6**
**10**	32.65 ± 2.1	36.44 ± 2.2
**11**	87.36 ± 4.3	74.18 ± 3.7
**12**	51.74 ± 2.9	59.31 ± 3.3
**13**	37.10 ± 2.3	41.42 ± 2.5
**14**	**12.94 ± 1.1**	**19.59 ± 1.4**
**Doxorubicin**	4.17 ± 0.2	4.50 ± 0.2
**Erlotinib** [1]	8.20 ± 0.34	7.73 ± 0.67
**Sorafenib**	7.26 ± 1.3	9.18 ± 0.6

Table S2. Enzyme assay (IC_{50}, µg/mL)

Compound no.	EGFR (Wild-Type)	EGFR (Mutant (T790M))	VEGFR-2
**2**	**16.25**	**17.8**	242.94
**8**	28.06	50.9	**35.85**
**14**	**16.33**	**16.6**	112.36
**Erlotinib**	9.69	4.13	-
**Sorafenib**	-	-	5.86

Table S3. Results of cell cycle analysis in MCF-7 expressed by (%).

Code	%G0-G1	%S	%G2/M	Comment
**Compound 14**	**74.16**	19.22	6.62	Cell cycle arrest at G1phase
**Cont. MCF-7**	**55.31**	27.95	16.74	---

Table S4. Results of apoptosis & necrosis expressed by (%).

Code	Apoptosis			Necrosis
	Total	Early	Late	
**Compound 14**	26.32	**15.02**	**7.1**	**4.2**
**Cont. MCF-7**	3.19	**0.69**	**0.14**	**2.36**

Biological Assessments

S1. Analysis of Cytotoxic Inhibitory Concentration 50 (IC_{50}) with respect to HepG2 and MCF-7

The cytotoxic activity of the synthesized compounds was evaluated against MCF-7 and HepG2 cell lines using the 3-(4,5-dimethylthiazol-2-yl)-2,5-diphenyltetrazolium bromide (MTT) assay [2-5]. Cells were cultured in RPMI-1640 medium supplemented with 10% FBS, 100 U/mL penicillin, and 100 µg/mL streptomycin, then seeded in 96-well plates (1×10^4 cells/well) and incubated at 37 °C with 5% CO_2 for 48 h. Test compounds, prepared by serial dilution, were added for 24 h, after which 20 µL of MTT solution (5 mg/mL) was introduced and incubated for 4 h. Formazan crystals were dissolved in 100 µL DMSO, and absorbance was recorded at 570 nm using a microplate reader (EXL 800, USA). Cell viability was calculated relative to untreated controls, and IC_{50} values were obtained from dose–response curves by nonlinear regression. All assays were run in triplicate, and data are expressed as mean IC_{50} values (µM). Doxorubicin, erlotinib, and sorafenib served as reference drugs.

S2. EGFR Enzyme Inhibition Assay (Wild and Mutant (T790M) types) and VEGFR-2 Enzyme Inhibition Assay

The EGFR inhibitory activity of the test compounds was assessed against both wild-type and T790M mutant enzymes using commercial assay kits (Cat. #40321 and #40323). Experiments were performed in 96-well plates containing purified recombinant EGFR. Each well received 25 µL of master reaction mix, followed by 5 µL of either test inhibitor solution or inhibitor buffer (positive control). Blank wells additionally contained 20 µL of 1× kinase assay buffer. The reaction was initiated with 20 µL of diluted EGFR enzyme and incubated at 30 °C for 40 min. After incubation, 50 µL of Kinase-Glo Max reagent was added, and the plates were kept at room temperature in the dark for 15 min. Luminescence was then recorded using a microplate ELISA reader. Erlotinib served as the reference inhibitor [6].

VEGFR-2 inhibition was tested using the HTScan® Tyrosine Kinase Assay (Cat. #7788, Cell Signaling Technology). A 4× kinase buffer [240 mM HEPES (4-(2-hydroxyethyl)-1-piperazineethanesulfonic acid), pH 7.5; 20 mM $MgCl_2$; 20 mM $MnCl_2$; 12 µM Na_3VO_4] was supplemented with dithiothreitol (DTT, 10 µL of 1.25 M per 2.5 mL buffer), and 0.6 mL was used per enzyme tube. Each reaction well contained 12.5 µL of this mixture plus 12.5 µL test compound, incubated for 5 min at room temperature. Reactions were initiated with 25 µL of a 2× ATP/substrate cocktail, incubated for 30 min, and stopped with 50 µL of ethylenediaminetetraacetic acid (EDTA) (50 mM, pH 8.0). To detect activity, 25 µL of the stopped reaction was transferred to streptavidin-coated plates and processed with antibody–HRP/TMB (3,3′,5,5′-tetramethylbenzidine) colorimetric

detection. Absorbance was read at 450 nm, and IC_{50} values were determined from dose–response curves. Sorafenib served as the reference VEGFR-2 inhibitor [7].

S3. Assessment of the Impact of Compound 14 on Cell Cycle Progression and Apoptosis in MCF-7 Cells

MCF-7 breast cancer cells were seeded under standard culture conditions and treated with 10 µM of compound **14** for 48 h. After treatment, cells were harvested by trypsinization, washed with PBS, and fixed in ice-cold 66% ethanol at 4 °C for at least 2 h. Prior to analysis, cells were washed again with PBS and stained using the Propidium Iodide Flow Cytometry Kit (ab139418, Abcam, UK) according to the manufacturer's instructions. Staining was carried out with PI and RNase at 37 °C in the dark for 30 min. Cell cycle distribution (G0/G1, S, and G2/M phases) was then determined by measuring PI fluorescence intensity in the FL2 channel [8].

The pro-apoptotic activity of compound **14** was assessed using the Annexin V-FITC Apoptosis Detection Kit (BioVision, USA; Cat. No. K101-25) according to the manufacturer's instructions. MCF-7 cells (1–5 × 10^5) were seeded and treated with 10 µM of 14 for 48 h. After treatment, both floating and adherent cells were collected by gentle trypsinization, washed with serum-containing medium, and resuspended in 500 µL of 1× Binding Buffer. Each sample was stained with 5 µL Annexin V-FITC and 5 µL propidium iodide (50 µg/mL) for 5 min at room temperature in the dark, then analyzed by flow cytometry. Annexin V-FITC fluorescence was detected in FL1 (Ex = 488 nm, Em = 530 nm) and PI in FL2 to distinguish viable, early apoptotic, late apoptotic, and necrotic populations [9].

References

1. N. A. Aziz, R. F. George, K. El-Adl and W. R. J. R. a. Mahmoud, 2022, **12**, 12913-12931.
2. T. J. J. o. i. m. Mosmann, 1983, **65**, 55-63.
3. F. Denizot and R. Lang, *Journal of immunological methods*, 1986, **89**, 271-277.
4. N. El-Gohary, S. Hawas, M. Gabr, M. Shaaban and M. J. B. C. El-Ashmawy, 2019, **92**, 103109.
5. S. S. Hawas, N. S. El-Gohary, M. T. Gabr, M. I. Shaaban and M. B. J. S. C. El-Ashmawy, 2019, **49**, 2466-2487.
6. M. A. A. Fathi, A. A. Abd El-Hafeez, D. Abdelhamid, S. H. Abbas, M. M. Montano and M. J. B. c. Abdel-Aziz, 2019, **84**, 150-163.
7. M. A. Abdelgawad, A. M. Hayallah, S. N. A. Bukhari, A. Musa, M. Elmowafy, H. M. Abdel-Rahman and M. K. J. P. Abd El-Gaber, 2022, **15**, 1416.
8. J. Wang and M. J. J. J. o. C. S. Lenardo, 2000, **113**, 753-757.
9. K. K.-W. Lo, T. K.-M. Lee, J. S.-Y. Lau, W.-L. Poon and S.-H. J. I. c. Cheng, 2008, **47**, 200-208.
